# High-coverage whole-genome sequencing of a Jakun individual from the “*Orang Asli*” Proto-Malay subtribe from Peninsular Malaysia

**DOI:** 10.1038/s41439-024-00308-6

**Published:** 2025-01-08

**Authors:** Wai-Sum Yap, Alvin Cengnata, Woei-Yuh Saw, Thuhairah Abdul Rahman, Yik-Ying Teo, Renee Lay-Hong Lim, Boon-Peng Hoh

**Affiliations:** 1 Department of Biotechnology, Faculty of Applied Sciences, UCSI University, Federal Territory of Kuala Lumpur, Kuala Lumpur Malaysia; 2 Saw Swee Hock School of Public Health National University of Singapore, Singapore, Singapore; 3 Life Sciences Institute, National University of Singapore, Singapore, Singapore; 4 Clinical Pathology Diagnostic Centre Research Laboratory, Faculty of Medicine, Universiti Teknologi MARA, Sungai Buloh Campus, Sungai Buloh, Selangor Malaysia; 5 Department of Statistics and Applied Probability, Faculty of Science, National University of Singapore, Singapore, Singapore; 6 NUS Graduate School for Integrative Science and Engineering National University of Singapore, Singapore, Singapore; 7 Genome Institute of Singapore Agency for Science, Technology and Research, Singapore, Singapore; 8 Faculty of Medicine and Health Sciences, UCSI University, Negeri Sembilan Federal Territory of Kuala Lumpur, Malaysia; 9 Present Address: Division of Applied Biomedical Sciences and Biotechnology, School of Health Sciences, IMU University, Bukit Jalil, Kuala Lumpur Federal Territory of Kuala Lumpur, Malaysia

**Keywords:** Genomics, Genetic variation

## Abstract

Jakun, a Proto-Malay subtribe from Peninsular Malaysia, is believed to have inhabited the Malay Archipelago during the period of agricultural expansion approximately 4 thousand years ago (kya). However, their genetic structure and population history remain inconclusive. In this study, we report the genome structure of a Jakun female, based on whole-genome sequencing, which yielded an average coverage of 35.97-fold. We identified approximately 3.6 million single-nucleotide variations (SNVs) and 517,784 small insertions/deletions (indels). Of these, 39,916 SNVs were novel (referencing dbSNP151), and 10,167 were nonsynonymous (nsSNVs), spanning 5674 genes. Principal Component Analysis (PCA) revealed that the Jakun genome sequence closely clustered with the genomes of the Cambodians (CAM) and the Metropolitan Malays from Singapore (SG_MAS). The ADMIXTURE analysis further revealed potential admixture from the EA and North Borneo populations, as corroborated by the results from the F3, F4, and TreeMix analyses. Mitochondrial DNA analysis revealed that the Jakun genome carried the N21a haplogroup (estimated to have occurred ~19 kya), which is commonly found among Malays from Malaysia and Indonesia. From the whole-genome sequence data, we identified 825 damaging and deleterious nonsynonymous single-nucleotide polymorphisms (nsSNVs) affecting 720 genes. Some of these variants are associated with age-related macular degeneration, atrial fibrillation, and HDL cholesterol level. Additionally, we located a total of 3310 variants on 32 core adsorption, distribution, metabolism, and elimination (ADME) genes. Of these, 193 variants are listed in PharmGKB, and 21 are nsSNVs. In summary, the genetic structure identified in the Jakun individual could enhance the mapping of genetic variants for disease-based population studies and further our understanding of the human migration history in Southeast Asia.

## Genetic study of Jakun sheds light on population history

The Orang Asli are the indigenous people of Peninsular Malaysia, with the Jakun being one of the largest sub-tribes. Researchers conducted a study to explore the genome of a Jakun female. They used whole-genome sequencing to analyze her DNA, which involves reading the entire genetic code to identify variations. This study aimed to understand the genetic diversity and ancestry of the Jakun people. Researchers collected a blood sample from an 18-year-old Jakun woman and sequenced her genome. They compared her genetic data with other Southeast Asian populations using various statistical methods like Principal Component Analysis, ADMIXTURE, and F-Statistics, which help identify genetic similarities and differences. The study found that the Jakun genome shares close genetic ties with neighboring Southeast Asian populations, in particular, a genetic interaction with North Borneo natives was observed. This summary was initially drafted using artificial intelligence, then revised and fact-checked by the author.

## Introduction

**Fig. 1:**
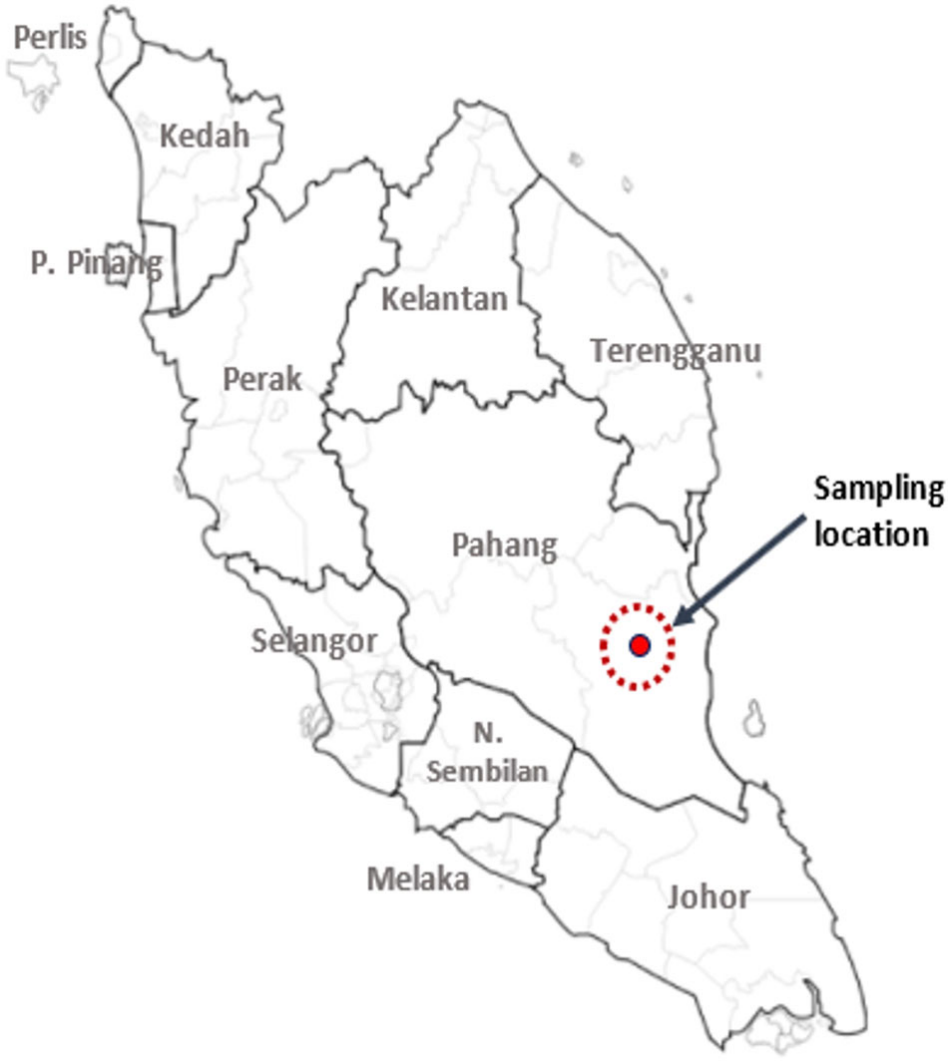
Geographical map of Peninsular Malaysia. The sampling location for the Jakun individual used in this study is highlighted in solid red.

“Orang Asli” is a Malay term that means “original people” or “first people”. It is broadly applied to the indigenous people of Peninsular Malaysia. There are three main ethno-linguistic groups of Orang Asli: Negrito, Senoi, and Proto-Malay. The Proto-Malays, believed to have inhabited Peninsular Malaysia approximately four thousand years ago (4 kya)^1^, are mostly found in the central and southern regions of the peninsula (Fig. 1).

There are six subtribes that fall under the Proto-Malay classification: the Jakun, Orang Kanaq, Orang Kuala, Orang Seletar, Semelai, and Temuan. The Jakun are believed to be the earliest Proto-Malay settlers around Chini Lake, located in the central part of Peninsular Malaysia. They traditionally practiced primitive agriculture, hunting and gathering, and fishing^2^. Today, they reside primarily in the central and southern parts of Peninsular Malaysia. Over the past few decades, the Jakun people have been exposed to the country’s economic development, adopting permanent agriculture and managing their own plantations of rubber, oil palm, and cocoa^3^. Despite being the largest Proto-Malay subtribe on record, with a population of approximately 34,722, the genetic architecture of this subtribe is not fully understood^4^.

Comprehensive genetic study of the Orang Asli population provides unprecedented insights into human migration in the Malay Archipelago, which is located in Southeast Asia (SEA)^1^. Previous studies on isolated populations, such as Finns, Icelanders, and Greeks, have demonstrated significant potential in the identification and/or enrichment of rare and low-frequency single-nucleotide variants, as well as novel loci with medically relevant traits^5^. Although there are an increasing number of recent publications on the population genetics of Orang Asli, most of these studies have focused on (i) genotyping or SNP array data^4,6,7^, (ii) genetic polymorphisms of selected genes^8^, or (iii) mtDNA analysis^9^. As sequencing costs become more affordable and knowledge becomes more accessible, whole-genome sequencing (WGS) is recognized as a powerful tool for identifying key variants in diverse populations. An enriched genetic map with population-unique (or rare) variants allows the development of pharmacogenomic (PGx) markers that might be undetected using conventional genetic screening methods^10^. Although several WGS-based studies are available^11,12^, no such studies have been reported in Jakun.

Recently, the whole-genome sequences of 12 Orang Asli and natives from North Borneo have been reported^12^, and their genomic structure was determined using trio analysis^13^. However, the genomic structure of the Proto-Malay remains unresolved. Because they are descendants of the Austronesian language family, similar to the majority of the Southeast Asia (SEA) population, dissecting their genomic structure will provide a better understanding of population ancestries. This can shed light on precision medicine implications for SEA populations, which have been underinvestigated until recently^14^.

In this study, we analyzed the genome architecture of an individual Jakun female and confirmed, based on principal component analysis (PCA), ADMIXTURE, tree topology analysis, and F statistics, that her genome was closely related to those of neighboring Southeast Asian (SEA) populations. We subsequently investigated the genetic variation in the absorption, distribution, metabolism, and excretion (ADME) genes of the Jakun genome, revealing numerous novel nonsynonymous variants (nsSNVs) within the core ADME genes.

## Materials and methods

### Sample recruitment, collection, and extraction

This study was approved by the Research and Ethics Committee of Universiti Teknologi MARA [Ref no: 600-RMI (5/1/6)] and the Department of *Orang Asli* Development (Jabatan Kemajuan Orang Asli Malaysia, JAKOA) [JHEOA.PP.30.052. Jld 5(17)]. Prior to sample collection, customary visits to the tribe headman and the community members were conducted to thoroughly explain the study and to obtain their approval to conduct this research in their settlement. A Jakun female subject (hereafter abbreviated as Jakun_Seq) residing at Chini Lake (3.4336° N, 102.9189° E) was selected randomly for this study. During sampling, informed consent was obtained from this subject, whereby the entire interview process (e.g., to obtain family history, pedigree, and self-reported ethnicity) was conducted in the Malay language and witnessed by an accompanying JAKOA officer.

### Sample phenotype

The Jakun female subject was 18 years old and had a normal blood pressure (114/78.5 mmHg) and glucose level (HbA1c = 5) but a slightly lower body mass index (BMI of 17.5). Her high-density lipoprotein cholesterol (HDL-c) concentration was 1.18 mmol/L, and she had a low level of low-density lipoprotein cholesterol (LDL-c of 2.38 mmol/L) and slightly higher triglyceride levels (1.72 mmol/L). This subject was found to have hyperalbuminemia (51.6 g/L) and had an insulin level above average (30.8 units/ml). A history of previous infections was not taken, and no other medical history (including hypersensitivity) was reported by the subject or her family during the interview.

### Generation of sequencing data

Genomic DNA was extracted from 8 mL of the subject’s blood sample using a commercially available kit (QIAamp DNA Minikit, Hilden, Germany), and whole-genome sequencing was performed using the HiSeq 2000 platform (Illumina Inc., San Diego, CA) according to the manufacturer’s instructions. DNA quantification was first performed using a Nanodrop (Thermo Fisher Scientific), followed by Picogreen® and a Bioanalyzer (Agilent Tech Inc.), whereby the fluorescence intensity was measured at 480 nm/520 nm to ensure that the DNA yield and integrity met the requirements for whole-genome sequencing. The DNA concentration was set at approximately 50 ng/μL, with the OD 260/280 reading ranging from 1.8–2.2. Upon completion of DNA sequencing, approximately 36× sequencing coverage was generated from 100 bp paired-end reads, with insert sizes of 300–400 bp.

The genome sequence data of the samples has been submitted to the European Genome–Phenome Archive (EGA) (https://ega-archive.org/) with the assigned accession number EGAD50000001024.

### Data source

The reference genome from the UCSC Genome Browser (hg19, February 2009; www.genome.ucsc.edu) and dbSNP Build 151 database were used as references for SNV genome annotations (ftp://ftp.ncbi.nlm.nih.gov). The genomes used for comparison via principal component analysis (PCA), ADMIXTURE, F3 and F4 statistics, and TreeMix were obtained from (i) the Hapmap3 panel database (ftp://ftp.ncbi.nlm.nih.gov/hapmap), with 60 randomly selected individuals belonging to seven different populations (CEU, CHD, CHB, JPT, GIH, YRI); (ii) the Singapore Genome Variation Project (SGVP) panel database (http://phg.nus.edu.sg/StatGen/public_html/SGVP/download.html), with 30 randomly selected individuals (10 samples each for SG_CHS, SG_INS and SG_MAS); (iii) the HGDP-CEPH database (ftp://ftp.cephb.fr/hgdp_supp3/), with 10 randomly selected CAM individuals; and (iv) an in-house database from previous studies^4,15^. The in-house database included 50 individuals from North Borneo (or better known as Sabah)^15^ (10 samples each for Dusun, Lingkabau, Murut-Paluan, Rungus, and Sonsogon), and a panel of 30 Orang Asli individuals^4^ (10 samples each for Negrito Bateq, Senoi CheWong, and Proto-Malay Jakun). Although we employed all the SNP array data that we obtained for this analysis, specific combinations of these data were also utilized for population admixture and phylogenetic analysis. The Jakun SNP array data and Jakun WGS data are denoted as “Jakun_Geno” and “Jakun_Seq”, respectively. A summary of the datasets utilized is presented in Table S1.

### Mapping and alignment to the reference genome

The genome sequences were aligned with the human reference genome (hg19) using the Burrows–Wheeler Aligner (BWA; Version: 0.7.5a-r405)^16^ with *bwa mem -M -t 7 -p* and SAMtools 1.3.1^17^ with the default options. The alignment files were then merged into a single BAM file, which was marked for duplicates using Picard tools-1.119 (http://picard.sourceforge.net), and the base quality scores were recalibrated using the Genome Analysis Toolkit (GATK v4.0.12)^18^.

### SNV and indel identification

SNVs and short indels (defined as insertions/deletions with sizes ranging from 1–50 bp)^19^ were identified using the Genome Analysis Toolkit (GATK v4.0.12) in accordance with GATK best practices. After the base quality score recalibration, Haplotypecaller with *--genotyping_mode DISCOVERY*, *-stand_emit_conf 10 and -stand_call_conf 30* was used, followed by VariantAnnotator using dbSNP Build 151, VariantRecalibrator for *-mode SNP* and *INDEL*, respectively. Finally, ApplyRecalibration was carried out with *--ts_filter_level 99.9* to obtain the recalibrated vcf file. The effects of variants on genes (such as nonsynonymous variants) were further predicted using the snpEFF version 4.3i^20^ prediction tool. All nonsynonymous variants identified using snpEFF were then annotated with SIFT Human SNVs^21^, PolyPhen2^22^, and combined annotation-dependent depletion (CADD)^23^ results were compared, whereby common damaging and deleterious variants were retrieved to avoid conflicting inference.

To characterize the genetic variation of ADME genes, nonsynonymous SNVs (nsSNVs) with functional abnormalities from the Jakun genome were annotated against the publicly available PharmGKB dataset to investigate their possible associations with drug transport, metabolism and drug targets^24^.

### Population admixture and phylogenetic analysis

The extent of genetic heterogeneity between the Jakun and global reference populations was evaluated using (i) PCA, (ii) ADMIXTURE, (iii) F3 and F4 statistics, and (iv) TreeMix analyses. These analyses were performed across various selected populations (details in the following paragraph).

All the data obtained from different resources were merged using PLINK v1.90b3.46 64-bit^25^. The detailed flow of the data merging process is shown in Fig. S1. First, overlapping SNVs among all populations were extracted, and SNVs absent in any of the datasets were excluded. Subsequently, SNVs with duplication and missingness >0.1, individuals with first-degree kinship, and Hardy–Weinberg Equilibrium (HWE) with *P* < 1 × 10^−5^ were removed. Finally, the data were thinned (pruned) using *--indep-pairwise 200 25 0.4* (200 base-pair sliding windows, advancing in steps of 25, removing any SNV with R^2^ greater than 0.4 with any other SNV within the same window).

After pruning, the following number of overlapping SNVs remained: (a) global population: 136,972 SNVs overlapped among 18 populations; (b) East Asian (EA) and Southeast Asian (SEA) populations: 124,521 SNVs overlapped across 14 populations; and (c) SEA populations: 121,684 SNVs overlapped across 10 populations. In our population count, we considered Jakun_Seq and Jakun_Geno as a single population. The SNVs mentioned above were utilized for the following analyses: (i) PCA using the smartPCA program from the EIGENSOFT 6.1.4 package^26^ and (ii) ADMIXTURE version 1.3.0^27^ to identify the presence of diverse ancestral relationships between the Jakun genome and other populations. In ADMIXTURE, values of K (ranging from 2–9) that yielded the lowest cross-validation error were selected.

We subsequently employed TreeMix (version 1.13)^28^ for TreeMix analysis and Admixtools (version 2.0.8)^29,30^ for F3 and F4 analyses to infer the evolutionary relationships between the Jakun population and other groups, pinpointing admixture patterns and measuring gene flows. Briefly, we constructed a maximum-likelihood drift tree (TreeMix) for selected populations and performed 100 independent replications for each population^31–33^, allowing 1–10 migration events, using the YRI genome as the root. The F3 statistic specifically identifies historical admixture events, whereas the F4 statistic allows the identification of intricate admixture patterns and possible directional flow of genes between populations.

### Gene annotation and enrichment analysis

The consensus sequences of damaging and deleterious nsSNVs, along with their corresponding genes obtained from SIFT, PolyPhen2, and CADD, were annotated using the Database for Annotation, Visualization, and Integrated Discovery (DAVID) (http://david.abcc.ncifcrf.gov/)^34^. The results obtained under the default conditions in DAVID (i.e., a minimum of 2 genes per term, EASE score 0.10) were downloaded. The categories for annotations were as follows: (i) UP_KEYWORDS for Functional_Categories; (ii) GOTERM_BP_DIRECT, GOTERM_CC_DIRECT, and GOTERM_MF_DIRECT within Gene_Ontology; and (iii) KEGG_PATHWAY for Pathways. The *P*-values for the significance of enrichment or overrepresentation of terms for each annotation were adjusted using the Benjamini‒Hochberg FDR. The SNVs (rsID#) corresponding to the 720 genes identified were cross-referenced using db151, and the rsID# values were searched for significant reported traits in the GWAS Catalog (https://www.ebi.ac.uk/gwas/search?query=rs749296565)^35^.

## Results

### Genome sequencing and variant identification

A total of 3,135,673,168 clean reads were obtained from the Jakun subject’s genome after the removal of poor or biased sequences, ambiguous bases, and read duplicates. These reads were aligned to the human reference genome (excluding Ns, resulting in 2,895,822,366 mapped reads), covering 92.35% of the reference genome with an average sequencing depth of 35.97x (Fig. S1). A total of 3,595,557 SNVs were called, 3,555,641 of which (98.89%) had been reported in dbSNP Build 151, whereas the remaining 39,916 were novel (Table 1). The distribution of these novel SNVs across each autosome is shown in Fig. S2. On average, 1.11% of novel SNVs were detected in the whole-genome, with chromosome 17 having the highest percentage at 2.11%. We identified 1,597,422 homozygous and 1,998,135 heterozygous SNVs (approximate ratio of 1:1.251). Among these, 68,335 SNVs (1.90%) were located in coding DNA sequence (CDS) regions, 59,984 (1.67%) in 3′ untranslated regions (UTRs), and 11,482 (0.32%) in 5′ UTRs. Additionally, 10,167 nsSNVs distributed across 5674 genes were identified (Fig. S3).

**Table 1:** Summary of SNVs found in the Jakun_Seq genome mapped to dbSNP151.

Total SNVs	Homozygous SNVs	Heterozygous SNVs	SNVs mapped to dbSNP (Build 151)	% SNVs mapped to dbSNP	Novel SNVs	% Novel SNVs
3,595,557	1,597,422	1,998,135	3,555,641	98.89%	39,916	1.11%

Among the 517,784 short indels identified, 254,202 were found in intergenic regions, 653 in exonic regions, and 210,170 in intronic regions. Notably, among these indels, the *PLBD1* (phospholipase B-like 1) gene stands out because it contains a nonsynonymous variant (rs1219166053). This gene encodes a protein that plays a key role in lipid catabolism. An insertion of a T nucleotide at chr12:14,720,554 in this gene results in the substitution of proline with glutamine (P26Q), which is situated in the N-terminal signal peptide (aa positions 1–38) based on UniProtKB. Moreover, the insertion of the T nucleotide causes a frameshift in the *PLBD1* gene or represents a 2 kb upstream variant of the *PLBD1-AS1* gene (https://www.ncbi.nlm.nih.gov/snp/rs1219166053#submissions; accessed on 10 Sep 2024). The clinical significance of this variant, however, remains to be assessed.

### Admixture and phylogenetic analyses

**Fig. 2:**
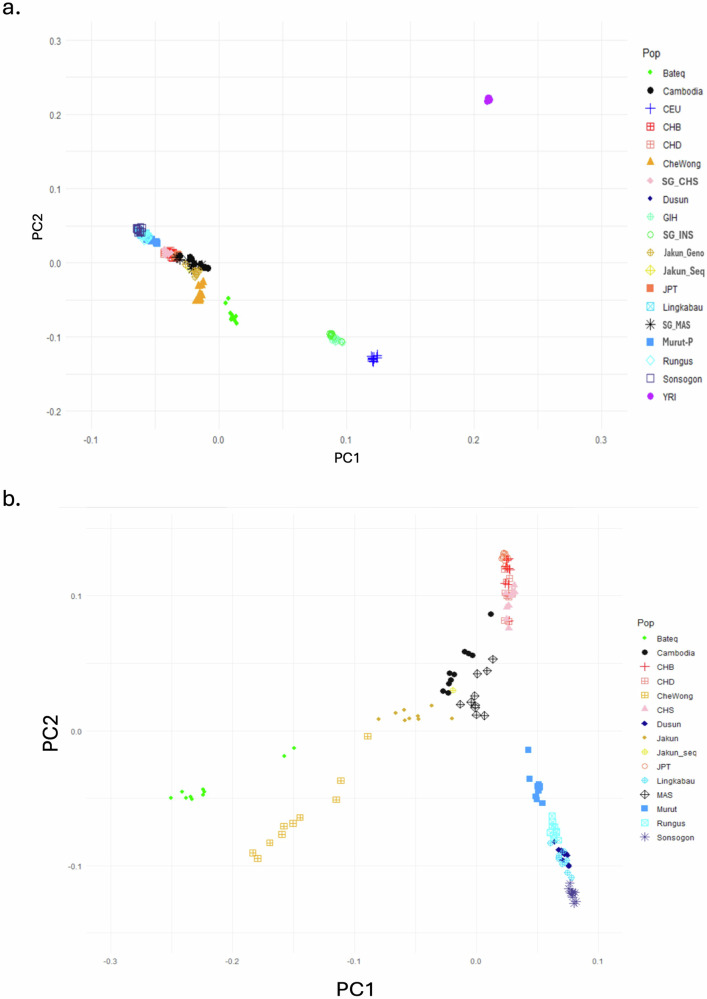
Principal component analysis (PCA) of the Jakun_Seq individual. **a** Global populations; (**b**) East Asian and Southeast Asian populations; and (**c**) Southeast Asian populations. The red arrow indicates the Jakun_Seq in this study.

To determine the representation of the genome of the Jakun tribe, the population structure of this sequenced genome was assessed on a global scale using PCA, ADMIXTURE, and TreeMix analyses. The PCA results revealed that this Jakun_Seq genome clusters closely with SNP array data from (i) other Jakun samples, (ii) SEA samples (including Cambodia, SG_MAS, Dusun, Lingkabau, Murut-P, Rungus, and Sonsogon), and (iii) East Asian samples (such as CHB, CHD, SG_CHS, and JPT). However, this population is distinctly separated (Fig. 2a) from South Asian (GIH and SG_INS), European (CEU), and African (YRI) samples, which is consistent with previous reports. In a detailed analysis of the genetic relationships within EA and SEA (Fig. 2b), Jakun_Seq appeared distant from the two subtribe groups of Bateq and CheWong, as well as from the North Borneo populations. However, it displayed a closer affinity to the Cambodia and SG_MAS clusters. These findings suggest that Jakun_Seq has a stronger genetic link to populations from the SEA mainland than to the Orang Asli subtribes of Bateq and CheWong, which are known to belong to the Austroasiatic language family.

**Fig. 3:**
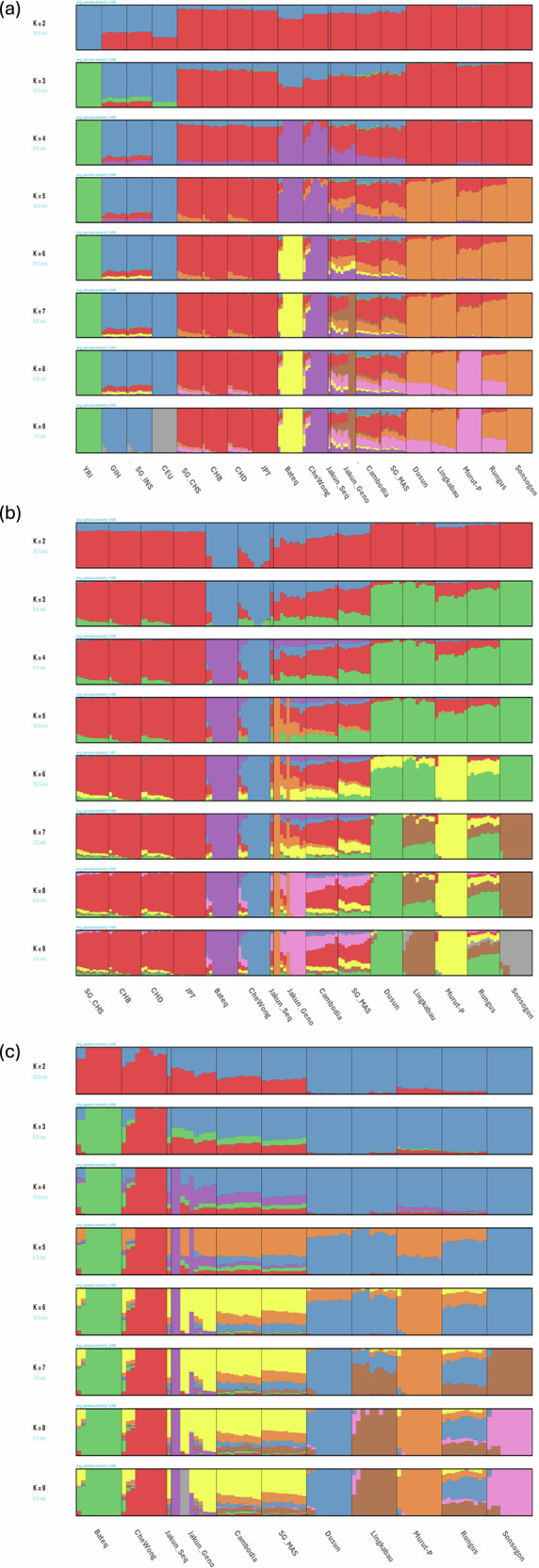
Admixture results of the Jakun individual. **a** Global populations; (**b**) East Asian and Southeast Asian populations; and (**c**) Southeast Asian populations. Each population is represented by a vertical line divided into colored segments that represent membership coefficients in the subgroups.

The ADMIXTURE analysis, when applied to global populations, estimated the cross-validation error (Fig. S4), suggesting an optimal division into five to seven ancestral components (*K* = 5–7). The admixture of global populations (Fig. 3a) revealed that the ancestral components of the Jakun_Seq genome align closely with those of other Jakun_Geno samples, underscoring the similarity between this single individual and the broader population. At *K* = 5, the Jakun_Seq genome exhibited an admixture of the East Asia ancestral component (represented by the red component). Interestingly, it also carried a unique ancestral component from the Orang Asli tribes “Bateq” and “CheWong” (highlighted in purple). This component was shared, albeit to a lesser extent, with the Cambodia and SG_MAS populations. This is presumably the “Austroasiatic Orang Asli” component (Fig. 3a). At *K* = 6, the Jakun_Seq genome is composed of 48.4% of an EA component, 22.2% of a North Borneo component (orange), approximately 12.4% of a CheWong component (purple, presumably the Austroasiatic-related ancestral component), 7.9% of European and South Asian components (blue), and another 8.2% of a Bateq component (yellow) (Table S2). At *K* = 7, a distinct ancestral component that is plausibly specific to Jakun emerges (represented in brown). This is in line with the regional ADMIXTURE analysis, as depicted in Fig. 3b, c.

**Fig. 4:**
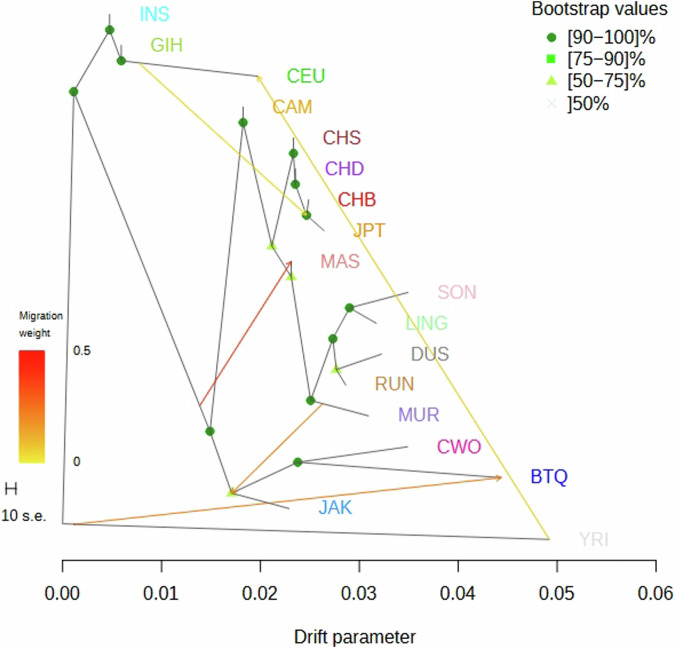
Maximum-likelihood tree generated using TreeMix with 100 bootstraps for Jakun at K = 5. A migration event was observed from the root of the common ancestor of North Borneo populations to the Jakun, implying a plausible shared common ancestor between the two populations.

We subsequently explored whether there was any gene flow between individual Jakun subjects and other contemporary human populations (Fig. S5), and with a migration setting of 0, Jakun (consisting of both Jakun_Seq and Jakun_Geno) was directly linked to a branch that subsequently split into nodes representing the Cambodian population, inferring close genetic relatedness between the two populations. At a count of five migration events (Fig. 4), a migration edge was observed on Jakun, originating from the ancestral branch of the North Borneo populations directed to Jakun, implying a plausible gene flow from the common ancestors of North Borneo populations to the common ancestor of Jakun or a genetic interaction between the ancestors of Jakun and North Borneo natives. This finding is in line with the observations from the ADMIXTURE analysis. Such interactions could arise from events such as intermarriage, migration, or other forms of population interaction^7,36^. However, owing to the lack of interactions between these populations, we suspect that the observed gene flow could have occurred prehistorically, but further investigations are needed to confirm this speculation.

The TreeMix results were further confirmed using the F3 and F4 statistics. According to the F3 statistical analysis of Jakun (Jakun_Seq+Jakun_Geno), populations from mainland SEA, North Borneo, and EA presented evidence of admixture or shared ancestry among these groups (Table S3a). In the F4 statistics, model f4 (Jakun, Bateq/Chewong; North Borneo/SEA/EA, Yoruba) (highlighted in blue font) suggested that Jakun appeared to be genetically more distant from the Bateq and CheWong than from populations in SEA, North Borneo and EA (Table S3b; results highlighted in blue font). Intriguingly, f4 (Jakun, North Borneo/SEA/EA; North Borneo/SEA/EA, Yoruba) revealed that North Borneo, SEA, and EA included in this study seemed to have a closer genetic connection than Jakun did (Table S3b; highlighted in red font).

### mtDNA analysis

The mitochondrial genome analysis, performed using the Haplogrep tool (http://haplogrep.uibk.ac.at/blog/rcrs-vs-rsrs-vs-hg19/#more-30)^37^, identified a total of 34 SNVs in the Jakun_Seq mtDNA. When these variants were annotated with the revised Cambridge reference sequence (rCRS), 32 out of the 34 SNVs placed Jakun_Seq in the subclade N21a haplogroup, a haplogroup commonly found among Malay populations from Malaysia and Indonesia^38^. This haplogroup has also been observed in other populations close to SEA, including the populations from Yunnan^39^, as well as in the Cham individuals of Bin Thuan, Vietnam, and it is believed to have occurred ~19 kya^40^.

### Functional annotation of nsSNVs

Annotation with snpEFF revealed 10,167 nsSNVs in the Jak_Seq genome. Among these 10,167 SNVs, a total of 1683, 1313, and 3727 were identified as either damaging or deleterious by the SIFT, Polyphen-2, and CADD (Phred score ≥ 15) computational tools, respectively. The consensus sequences from all three prediction tools classified 825 SNVs as functionally damaging and deleterious. These SNVs affected 720 genes (Table S4), which were further annotated using DAVID: Functional Annotation Tool (DAVID Knowledgebase v2024q2 released—5 July 2024) (Table 2).

**Table 2:** Enrichment analysis using DAVID utilizing 720 genes obtained by consensus of SIFT, PolyPhen2, and CADD (Phred score ≥ 15).

Term	GOTerm Accession No.	Fold-Enrichment	*P*-value	FDR
(i) GOTERM biological process				
Detection of chemical stimulus involved in sensory of smell	GO:0050911	4.5	6.5 × 10^−22^	1.3 × 10^−18^
G-protein coupled receptor signaling pathway	GO:0007186	2.5	7.4 × 10^−12^	7.6 × 10^−9^
(ii) GOTERM cellular component				
Basement membrane	GO:0005604	5.4	2.5 × 10^−7^	1.2 × 10^−4^
Plasma membrane	GO:0005886	1.3	2.8 × 10^−5^	6.7 × 10^−3^
Keratin filament	GO:0045095	3.5	1.2 × 10^−5^	8.5 × 10^−3^
Axonemal dynein complex	GO:0005858	11.5	1.2 × 10^−4^	1.9 × 10^−2^
Collagen-containing extracellular matrix	GO:0062023	2.2	1.6 × 10^−4^	1.9 × 10^−2^
(iii) GOTERM molecular function				
Olfactory receptor activity	GO:0004984	4.2	8.9 × 10^−21^	6.1 × 10^−18^
G-protein-coupled receptor activity	GO:0004930	2.8	2.0 × 10^−13^	6.7 × 10^−11^
Calcium ion binding	GO:0005509	2.0	2.3 × 10^−5^	5.1 × 10^−3^
Minus-end-directed microtubule motor activity	GO:0008569	10.6	1.8 × 10^−4^	3.1 × 10^−2^
(iv) KEGG pathway				
Olfactory transduction	-	4.2	1.9 × 10^−20^	4.9 × 10^−18^
ECM-receptor interaction		4.5	2.6 × 10^−5^	3.2 × 10^−3^
Cytoskeleton in muscle		2.5	5.0 × 10^−4^	4.1 × 10^−2^

The nsSNVs of this Jakun subject were enriched in the critical “sensory/olfactory” and “keratin filament” categories of gene ontology (Table 2). Olfaction is essential for the development of locomotor activities and spatial orientation in humans and is believed to be a crucial measure for the survival and regime of the Jakun in their tropical rainforest habitat^13,41^. Likewise, when combined with collagen and elastin in the extracellular matrix of the epidermal skin, keratin enhances its strength and almost waterproof nature^42^. The keratin enrichment found in this Jakun subject might serve as protection and adaptation against rubbing and pressure due to their primitive roles as agriculturists and hunter-gatherers^2^, as evidenced by the thickening of the outer, cornified layer of the skin to form protective calluses.

To assess the impact of the 825 damaging and deleterious nsSNVs on amino acid changes in the Jakun genome, these SNVs were searched for any reported traits in the GWAS Catalog (https://www.ebi.ac.uk/gwas/search; catalog v1.0.2.1, published Oct. 2023, revised August 2024). A total of 72 damaging and deleterious nsSNVs associated with specific traits were identified through a genome-wide association study (GWAS). These nsSNVs were identified using a genome-wide significance threshold of *p* < 5.0 × 10^−8^, ensuring the robustness of the findings. These variants, known to potentially impact protein function and contribute to disease susceptibility, were linked to 574 association studies.

However, not all these reported traits are associated with a risk allele. In the Jakun subject, 41 nucleotide changes were found to correlate with risk alleles, suggesting a potentially increased susceptibility to the traits listed in Table S5. Some of these traits include an increased percentage of monocytes (rs11557154; C > T), age-related macular degeneration (rs10490924; G > T), atrial fibrillation (rs2296610; G > T), obesity class 1 (rs11042023; T > C), a decreased waist‒hip ratio adjusted for BMI (rs3764002; C > T), increased HDL cholesterol (rs676210; G > A), increased systolic (rs2286394; C > T) and diastolic (rs1933437; G > A) blood pressures, and decreased coronary artery disease (rs11556924; C > T), among others. Moreover, we identified 17 (2.1%) novel variants in this Jakun subject. These variants and their associated genes are detailed in Table S6. The functional implications of these variants on their respective genes remain to be explored.

The allelic frequencies of the 72 nsSNVs reported in the GWAS Catalog (Table S5) were determined using databases from the 1000 Genomes Project Phase 3, HapMap Project, and gnomAD genomes, all of which are available at the gnomAD browser, version v4.1.0 (https://gnomad.broadinstitute.org/variant/; accessed 8 Oct 2024). The results revealed that three nsSNVs presented relatively high minor allele frequencies (MAFs) in either East Asian populations or the CAM population specifically. The average MAF values across all populations were as follows: (i) 0.24 for rs676210 (*APOB*), peaking at 0.73 for East Asian populations; (ii) 0.04 for rs2296610 (*NEBL*), with the highest value being 0.17 for East Asian populations; and (iii) 0.10 rs1047781 (*FUT2*), with its peak value at 0.60 for the CAM population.

The *APOB* gene encodes two forms of the apolipoprotein B protein: a truncated version known as apolipoprotein B-48 and a longer form called apolipoprotein B-100. Both proteins transport fats and fat-like substances, including cholesterol, through the blood. The risk allele G of the SNV rs676210, which is also an ancestral allele, has been associated with levels of oxidized low-density lipoprotein (LDL) in European populations^43^. In our study, the Jakun subject presented a G > A substitution, suggesting a potential decrease in oxidized LDL levels. Interestingly, East Asian populations demonstrated a relatively high MAF (0.72) for the A allele, suggesting a possible incomplete sweep^44^. Other noteworthy genes include *NEBL* and *FUT2*. The *NEBL* gene encodes the nebulin protein, which is crucial for muscle function. The variant rs2296610 (G > T) within this gene is associated with atrial fibrillation—an irregular and often rapid heart rhythm—in the Japanese population^45^.

The *FUT2* (fucosyltransferase 2) gene encodes an enzyme crucial for the final step in the soluble ABO blood group antigen synthesis pathway, particularly for the synthesis of the H antigen precursor, which is essential for producing blood group A and B antigens. The variant rs1047781 (A > T) within this gene has been linked to elevated serum carcinoembryonic antigen levels, psoriasis, and vitamin B12 levels^46–48^. However, at the time of assessment, none of these traits were evident in the Jakun. This discrepancy might be because most gene variant and trait association studies are based on Caucasian and Asian populations, with limited research on the native Orang Asli populations closely related to the Jakun. For both the *NEBL* and *FUT2* variants, the risk allele corresponds to the derived allele, not the ancestral allele. The average MAF is less than 1%, indicating their rarity.

Separately, we identified 825 damaging and deleterious nsSNVs in the Jakun subject. Among these genes, 23 were located in 22 candidate genes (Table S7) and were also present in the Cambodian and SG_MAS populations. Notably, several of these genes are associated with olfactory receptor activity (*OR10H2*, *OR4L1*, and *OR52N2*), suggesting potential similarities in gene-environment interactions among populations within the SEA region. Other notable genes include (i) *NPBF3*, part of the neuroblastoma breakpoint family; (ii) *APOL5*, from the apolipoprotein L gene family; (iii) *CHIA*, which plays a role in chitin degradation; and (iv) *TTN*, which encodes a prominent protein in striated muscle.

Interestingly, two nsSNVs were identified within the *TTN* gene in the Jakun subject: rs2288569 (C > T) and rs3829747 (C > T) (Table S7). Given that *TTN* is a large gene consisting of 363 exons and that mutations within it can lead to a range of skeletal and cardiac muscle diseases, these variants were further investigated using the TITINdb website (http://fraternalilab.kcl.ac.uk/TITINdb/)^49^. However, no disease associations were identified. The implications of these genetic variants, especially as they relate to sensory and movement outcomes in the Jakun subject, remain to be explored.

From a pharmacogenetic standpoint, genetic variants significantly influence variations in drug efficacy and responses between individuals and populations. Consequently, administering incorrect drug dosages can pose risks to those whose drug metabolism deviates from the “assumed average”. Using the PharmGKB website, we retrieved variants associated with the 32 core ADME genes, which encompassed 939 “ADME variants” (https://www.pharmgkb.org/—accessed on 3 Oct 2024). These variants served as a reference for identifying similar variants in the Jakun subject (Table S8). Our analysis revealed 3310 variants distributed across the 32 core ADME genes in our sample. Among these, 193 matched those listed on the PharmGKB website, with 21 being nsSNVs. The most frequently altered gene in this Jakun individual was solute carrier family 15 member 2 (*SLC15A2*), which had three nsSNVs [rs1143671 (C > T); rs1143672 (G > A); rs2257212 (C > T)]. Typically, this gene encodes a proton-coupled peptide transporter responsible for the absorption of small peptides, beta-lactam antibiotics, and other peptide-like drugs from the kidney’s tubular filtrate.

## Discussion

In this study, a detailed examination of the genomic composition of a Proto-Malay Jakun female (JAK) was carried out by comparing the population architecture of her genome with those of other deliberately selected global counterparts. Additionally, we identified and characterized novel nsSNVs, with a primary focus on the roles of the affiliated genes. Our primary focus was on genetic variations that might influence the functionality of genes governing ADME and their correlated phenotypic traits. This genomic landscape of the Proto-Malay tribe, represented by the Jakun_Seq genome, provides rich insight into the genetic ties and evolutionary history of SEA populations. The use of a variety of analytical approaches, including PCA, ADMIXTURE, TreeMix, F3, and F4, revealed a rich mosaic of genetic relationships.

The total number of SNVs found in the Jakun genome is comparable to counts reported for other populations, such as the SEA Malay population (3.6 million SNVs)^50^, the Pathan population from northwest Pakistan (3.8 million SNVs)^51^, the Vietnamese population (3.4 million SNVs)^52^, and the Malay population from Peninsular Malaysia (3.5 million SNVs)^53^. The percentage of novel SNVs discovered in the Jakun subject (1.11%) was lower than those reported previously. This discrepancy likely arises from the use of different versions of the reference database. In this study, we utilized the updated dbSNP Build 151 (released Oct. 6, 2017) as our mapping reference. Given that the number of available dbSNP reference SNVs (rsID#) has more than doubled from 324 million to 660 million, a smaller number of novel SNVs in this Jakun genome is expected.

From a historical perspective, the Orang Asli Proto-Malay group is believed to have migrated to Peninsular Malaysia approximately 4000 years ago^1^. In our analysis, the PCA results underscore that Jakun_Seq closely aligns with its regional counterparts, corroborating the results of previous studies^4,7,35,54,55^. This further reflects the interconnected evolutionary and geographical paths of these populations. Although the Jakun_Seq genome illustrates broader genetic divergence across global populations, a deeper dive revealed subtle genetic substructures within the SEA region, particularly compared with the Bateq and CheWong subtribes^4,7,54^.

The ADMIXTURE analysis reveals a rich tapestry of ancestral components within the Jakun, hinting at a storied past replete with migrations, admixtures, and perhaps cultural interchanges^7,54^. Notably, an ancestral component predominantly linked to the Bateq and CheWong Orang Asli tribes accentuates the Jakun’s unique genetic signature, aligning with linguistic studies signifying their affiliation with the Austroasiatic language family^54,56^. The emergence of a “Jakun-specific” component at *K* = 7 suggests potential genetic idiosyncrasies or epochs of historical isolation.

Our TreeMix analysis clearly illustrates the dynamic interactions of gene flow and migration events. These findings revealed that Jakun has recently shared ancestry with the Cambodian population. Moreover, there is a pronounced genetic connection between Jakun and groups from the SEA mainland, North Borneo, and East Asian origins^33,35,55^. This complex web of connections shapes the unique genetic tapestry of the region^7,56^. The F3 and F4 statistics further affirm these relationships, whereby various influences, such as environmental factors, cultural interactions, and political dynamics, may have shaped these genetic ties^57,58^. Interestingly, the pronounced genetic links to East Asian groups suggest that the Jakun ancestors were extensively interconnected, challenging the belief of their isolated past^54,59^. Our findings, including those of PCA, ADMIXTURE, TreeMix, and F statistics, are in line with those of previous studies^4,7,36,38,54–56^. Several migration events were detected among the HapMap populations in the TreeMix analyses. These unrealistic migration events are likely artifacts reflecting shared bias among the HapMap samples, probably due to batch bias between the different SNP array platforms.

Further mtDNA analysis revealed that Jakun_Seq belongs to the N21a haplogroup, which dates to approximately 19 kya and is rooted in N21, with N as its base^60^. The deeper lineages seem to be primarily restricted to mainland SEA and have been detected in Thailand, Vietnam, and Yunnan, China^38^. This observation aligns with the study reported by Fix^55^, who suggested that the Proto-Malays migrated from Central Asia (specifically Yunnan) to Peninsular Malaysia via Peninsular Indo-China. This migration theory is postulated based on cultural, linguistic, and artifact clues found throughout the history of these populations. Subsequent archeological and linguistic studies have proposed that proto-Austronesian speakers settled in Taiwan at approximately 4000 B.C. They then migrated southward to the SEA region, passing through the Philippines to reach Borneo, Sulawesi, Central Java, and Eastern Indonesia approximately 2500 years ago^55^. However, the gene flow observed between the Jakun and North Borneo remains puzzling. It is conceivable that they shared a common ancestor before the split of the peninsula and Borneo Island ~15 kya. Further investigations, especially those involving ancient genome analysis, are warranted.

Collectively, our study aligns with Fix’s argument^55^ and further supports the possibility of Jakun migration from East Asia (China) via the mainland of SEA (Cambodia) to Peninsular Malaysia. The discovery of the mtDNA haplogroup N21a in the Jakun genome, which is also common among the Chinese in Lijiang, Yunnan^38^, and the Cham individuals of Vietnam^39^ lends additional support to this theory. However, the possibility of bifurcating migration from East Asia (China) directly to the islands of SEA, whether in one or multiple waves, remains open.

Our analysis of the Jakun genome revealed a total of 825 damaging and deleterious nsSNVs. These genes were enriched predominantly in the “sensory/olfactory” category of gene ontology, as detailed in Table 2. Among these nsSNVs, twenty-three (referenced in Table S8) were detected in the Jakun, Cambodian, and Singapore Malay populations. Three of these genes are involved in olfactory activity: *OR10H2* (rs1806931), *OR4L1* (rs2775254), and *OR52N2* (rs8181512). Although no clinical significance has been demonstrated for these three variants based on ClinVar data, higher frequencies of the alternate allele were observed for *OR10H2* (C > T) and *OR4L1* (G > A) in East Asian populations than in other global populations, as per the 1000 Genomes database. Price and his team^61^ discovered that the ability to detect certain odors can be tied to our family lineage or where our ancestors originated. These findings suggest that the Jakun, Cambodia, and Malay populations might have shared a common ancestor. Alternatively, Alonso et al.^62^ reported that differences in olfactory receptor genes among individuals can be influenced by their geographical location and cultural background. For the Jakun subject in question, the more than 4-fold enrichment in the “sensory or olfactory” category of gene ontology could be attributed to their stationary existence as an isolated group at Chini Lake. This isolation might have preserved their unique odor detection and perception abilities, which could be integral to their survival and adaptation processes.

The enrichment of nsSNVs in keratinocytes was also observed in the Jakun subject (Table 2). Gene ontology enrichments related to keratinocytes have been found to harbor a high frequency of Neanderthal alleles^63^, suggesting that Neanderthal alleles might have aided modern humans in adapting to non-African environments. Similar enrichment was reported in Neanderthal-like genomic segments among the native populations of Peninsular Malaysia and Borneo^12^.

We were specifically interested in any genetic variants in the Proto-Malay (as represented by the Jakun genome) that are responsible for drug metabolism. We managed to identify core ADME genes with the highest mutational burden and then pinpointed variants (rsID#) associated with core ADME that had clinical annotations. The *CYP2D6* gene presented the highest mutational burden (0.6727%), whereas the *GSTT1* and *UGT1A1* genes were less affected (0.000% and 0.0307%, respectively). The *CYP2D6* gene encodes a member of the cytochrome P450 superfamily of monooxygenase enzymes, which catalyze many reactions involved in drug metabolism as well as the synthesis of cholesterol, steroids, and other lipids. Its substrates include antidepressants, antipsychotics, analgesics, antitussives, beta-adrenergic blocking agents, antiarrhythmics, and antiemetics. The enzyme encoded by the *CYP2D6* gene plays a crucial role in metabolizing approximately 25% of all clinically used drugs. Numerous genetic polymorphisms in this gene can affect enzyme activity and drug response^64^. Additionally, the *CYP2D6* gene is known to be highly polymorphic across different populations^64^. In the Jakun genome alone, 29 variants were discovered, four of which [rs1080983 (T > C); rs1080985 (C > G); rs1135840 (G > C); and rs16947 (A > G)] were identified as being associated with clinical annotations related to drugs such as metoprolol, dextromethorphan, and n-desmethyltamoxifen. Only approximately 50% of the allele frequency for the *CYP2D6* gene is believed to be functional among Asians and their close descendants^65^.

Additionally, Zhou^66^ reported that there is significant interindividual variation in the activity of *CYP2D6* within a given population. To date, at least 74 different human *CYP2D6* variant alleles (*2 to *75) and a series of subvariants have been identified^66^. According to PharmVar (https://www.pharmvar.org/gene/CYP2D6), the four variants present in this Jakun genome are associated with alleles CYP2D6*4.021 (rs1080983, rs1080985, & rs1153840) and CYP2D6*6.001 (rs16947). Individuals carrying the *4 nonfunctional allele in combination with another nonfunctional allele (*3, *4, *5, *6) may experience decreased metabolism/clearance of metoprolol and a more pronounced decrease in heart rate than patients with the *1/*1 genotype, who carry two functional alleles^67^. Metoprolol, one of the most commonly used β-blockers, is primarily prescribed for the treatment of hypertension and heart failure. However, other genetic and clinical factors can also influence the metabolism of and response to metoprolol.

Another core ADME gene with a relatively high mutational burden is *CYP2E1* (0.40%). Three variants [rs2070673 (Jakun genotype = AT), rs2070676 (Jakun genotype = CG), and rs2515641 (Jakun genotype = CT)] were found to be associated with clinical annotations. The cytochrome P450 family 2 subfamily E member 1 (CYP2E1) gene encodes an enzyme involved in the metabolism of endogenous substrates (e.g., acetone and fatty acids) and exogenous compounds, including industrial chemicals, environmental toxicants, and procarcinogens (e.g., benzene, nitrosamines, and vinyl chloride)^68^. Elevated *CYP2E1* enzyme activity has been linked to the progression from hepatofibrosis to hepatocarcinogenesis^69^. The rs2070676 variant with the CG genotype is associated with the pharmacogenomics of cisplatin-based chemotherapy in patients with ovarian cancer. Patients with the CG genotype and ovarian neoplasms, when treated with cisplatin and cyclophosphamide, may face a greater risk of severe emesis than those with the CC genotype^70^. Conversely, patients with the AT genotype for variant rs2070673, as opposed to the TT genotype, demonstrated a reduced likelihood of toxic liver disease when treated with cytarabine, fludarabine, gemtuzumab ozogamicin, and idarubicin. However, a greater likelihood of toxic liver disease was observed in patients with the AT genotype than in those with the AA genotype^71^. In the same study, Iacobucci et al.^71^ also reported that patients with the CT genotype for the rs2515641 variant, compared with those with the CC genotype, may have a reduced risk of toxic liver disease but an increased risk compared with patients with the TT genotype. Nevertheless, other genetic and clinical factors influencing patient responses to these drugs cannot be overlooked.

To date, there are more than 900 variants of core ADME genes that show relevant associations between variants and drug phenotypes. These were retrieved from PharmGKB (https://www.pharmgkb.org/; accessed 9 Oct 2024). Notably, in this Jakun, 3310 variants were identified within the core ADME genes, suggesting the presence of more variants that have yet to be elucidated. Of these 3310 variants, only 193 were listed in PharmGKB, implying that some of these variants might not be damaging and, therefore, are not included in the database. Nevertheless, the high number of variants found in the Jakun genome associated with core ADME genes is believed to be related to the presence of xenobiotics, compounds that the Jakun subtribe can easily access. The Jakun people maintain an age-old tradition of using traditional herbs as medicine, adhering firmly to their ancestral beliefs as a way of life^72^.

In contrast, no variant of the *GSTT1* gene was found in the Jakun genome. The *GSTT1* gene encodes the glutathione S-transferase (*GST*) theta 1 enzyme, a member of a superfamily of proteins that catalyze the conjugation of reduced glutathione to various electrophilic and hydrophobic compounds. It is highly possible that a null genotype is present in this Jakun subject, as the frequencies of the *GSTT1* null genotype have been observed to vary^73^. The most extensively studied variant of *GSTT1* is the null variant (designated as GSTT1*0 or *GSTT1* negative), which results from the complete or partial deletion of the gene. Given that glutathione plays a role in the detoxification of many drugs, the *GSTT1* null variant has been linked to an increased risk of allergic skin reactions to various drugs, including NSAIDs and antibiotics^74^.

In summary, this study is the first to reveal the genomic structure of a Proto-Malay subtribe Jakun female from Peninsular Malaysia. Based on autosomal and mtDNA analyses, the Jakun genome shares a closer genetic affinity with neighboring SEA populations, particularly the Cambodia and Singapore Malay populations, highlighting a recent shared ancestry. We detected significant gene flow from the ancestor of the North Borneo populations to Jakun. These gene flows suggest prehistorical interactions, emphasizing Jakun’s intricate genetic tapestry shaped by diverse ancestral lineages. Notably, this is the first study to highlight the close genetic ties between the Jakun and North Borneo populations. Our enrichment analysis revealed a high enrichment score in the “sensory/olfactory” category of gene ontology, possibly inferring the significance of these variants with respect to adaptation to forest-based daily activities of the Jakun. Functional analysis revealed 825 damaging and deleterious nsSNVs in this genome, which are related to disease traits associated with obesity (rs11042023), HDL cholesterol (rs676210), atrial fibrillation (rs2296610), and age-related macular degeneration (rs10490924); decreased waist‒to-hip ratios adjusted for BMI (rs3764002); increased HDL cholesterol (rs676210); increased systolic (rs2286394) and diastolic (rs1933437) blood pressures; and decreased coronary artery disease (rs11556924), among others. This is the first comprehensive elucidation of a Jakun subtribe genome based on (i) functional analysis; (ii) identification of risk alleles associated with traits or diseases; and (iii) determination of nsSNVs associated with core ADME genes, providing foundational insights for future pharmacogenetic investigations.

## Supplementary information

Supplementary Information

Figure S1

Figure S2

Figure S3

Figure S4

Figure S5

Table S1

Table S2

Tabls S3

Table S4

Table S5

Table S6

Table S7

Table S8

Supplementary figure and table legend
