# Supplementary material for: High-coverage whole-genome sequencing of a Jakun individual from the “Orang Asli” Proto-Malay subtribe from Peninsular Malaysia
Source: Hum Genome Var. 2025 Jan 8;12:4. doi: 10.1038/s41439-024-00308-6 (PMC11707147; doi:10.1038/s41439-024-00308-6)
Supplement: Supplementary file 1 — Supplementary Information [file 41439_2024_308_MOESM1_ESM.docx]

**Supplementary Information**

**Supplementary Note:**

**Details protocol for SNV and indel identification**

- **Recalibrate Base Quality Scores = run BQSR**

1. Analyze patterns of covariation in the sequence dataset

java -Xmx4g -jar GenomeAnalysisTK.jar -T BaseRecalibrator -I JAK_sorted_realignindels.bam -R ucsc.hg19.fasta -knownSites db151­_hg19.vcf -knownSites Mills_and_1000G_gold_standard.indels.hg19.vcf -o JAK_sorted_realignindels_baserecal.data.table

1. Do a second pass to analyze covariation remaining after recalibration

java -jar GenomeAnalysisTK.jar -T BaseRecalibrator -R ucsc.hg19.fasta -I JAK_sorted_realignindels.bam -knownSites db151_hg19.vcf -knownSites Mills_and_1000G_gold_standard.indels.hg19.vcf -BQSR JAK_sorted_realignindels_baserecal.data.table -o JAK_sorted_realignindels_baserecal_POST_data.table

1. Generate before/after plots

java -jar GenomeAnalysisTK.jar -T AnalyzeCovariates -R ucsc.hg19.fasta -before JAK_sorted_realignindels_baserecal.data.table -after JAK_sorted_realignindels_baserecal_POST_data.table -plots JAK_sorted_realignindels_baserecal.data_plots.pdf

1. Apply the recalibration to your sequence data

java -Xmx4g -jar GenomeAnalysisTK.jar -T PrintReads -R ucsc.hg19.fasta -I JAK_sorted_realignindels.bam -BQSR JAK_sorted_realignindels_baserecal.data.table -o JAK_sorted_realignindels_baserecal_BQSRread.bam

- **Call variants with HaplotypeCaller**

java -jar GenomeAnalysisTK.jar -T HaplotypeCaller -R ucsc.hg19.fasta -I JAK_sorted_realignindels_baserecal_BQSRread.bam --genotyping_mode DISCOVERY -stand_emit_conf 10 -stand_call_conf 30 -o JAK_sorted_realignindels_baserecal_BQSRread_raw_variants.vcf

- **Variant Annotator – to annotate vcf file**

java -jar GenomeAnalysisTK.jar -T VariantAnnotator -R ucsc.hg19.fasta -I JAK_sorted_realignindels_baserecal_BQSRread.bam -V JAK_sorted_realignindels_baserecal_BQSRread_raw_variants.vcf -o JAK_sorted_realignindels_baserecal_BQSRread_raw_variants_annotated.vcf -A Coverage --dbsnp db151_hg19.vcf

- **VQSR – Build the SNP recalibration model**

java -jar GenomeAnalysisTK.jar -T VariantRecalibrator -R ucsc.hg19.fasta -input JAK_sorted_realignindels_baserecal_BQSRread_raw_variants_annotated.vcf -resource:hapmap,known=false,training=true,truth=true,prior=15.0 hapmap_3.3.hg19.vcf -resource:omni,known=false,training=true,truth=true,prior=12.0 1000G_omni2.5.hg19.vcf -resource:1000G,known=false,training=true,truth=false,prior=10.0 1000G_phase1.snps.high_confidence.hg19.vcf -resource:dbsnp,known=true,training=false,truth=false,prior=2.0 db151_hg19.vcf -an DP -an QD -an FS -an SOR -an MQ -an MQRankSum -an ReadPosRankSum -mode SNP -tranche 100.0 -tranche 99.9 -tranche 99.0 -tranche 90.0 -recalFile JAK_output_snp.recal -tranchesFile JAK_output_snp.tranches -rscriptFile JAK_output_snp.plots.R

- **VQSR – Apply the desired level of recalibration to the SNPs in the call set**

java -jar GenomeAnalysisTK.jar -T ApplyRecalibration -R ucsc.hg19.fasta -input JAK_sorted_realignindels_baserecal_BQSRread_raw_variants_annotated.vcf -mode SNP --ts_filter_level 99.9 -tranchesFile JAK_output_snp.tranches -recalFile JAK_output_snp.recal -o JAK_recalibrated_db151annotated_SNP.vcf

- **VQSR – Build the indel recalibration model**

java -jar GenomeAnalysisTK.jar -T VariantRecalibrator -R ucsc.hg19.fasta -input JAK_sorted_realignindels_baserecal_BQSRread_raw_variants_annotated.vcf --maxGaussians 4 -resource:mills,known=false,training=true,truth=true,prior=12.0 Mills_and_1000G_gold_standard.indels.hg19.vcf -resource:dbsnp,known=true,training=false,truth=false,prior=2.0 db151_hg19.vcf -an QD -an DP -an FS -an SOR -an MQRankSum -an ReadPosRankSum -mode INDEL -tranche 100.0 -tranche 99.9 -tranche 99.0 -tranche 90.0 -recalFile JAK_output_INDEL.recal -tranchesFile JAK_output_INDEL.tranches -rscriptFile JAK_output_INDEL.plots.R

- **VQSR – Apply the desired level of recalibration to the indels in the call set**

java -jar GenomeAnalysisTK.jar -T ApplyRecalibration -R ucsc.hg19.fasta -input JAK_sorted_realignindels_baserecal_BQSRread_raw_variants.vcf -mode INDEL --ts_filter_level 99.9 -tranchesFile JAK_output_INDEL.tranches -recalFile JAK_output_INDEL.recal -o JAK_recalibrated_INDELs.vcf
