# Supplementary figures and images for: High-coverage whole-genome sequencing of a Jakun individual from the “Orang Asli” Proto-Malay subtribe from Peninsular Malaysia
Source: Hum Genome Var. 2025 Jan 8;12:4. doi: 10.1038/s41439-024-00308-6 (PMC11707147; doi:10.1038/s41439-024-00308-6)

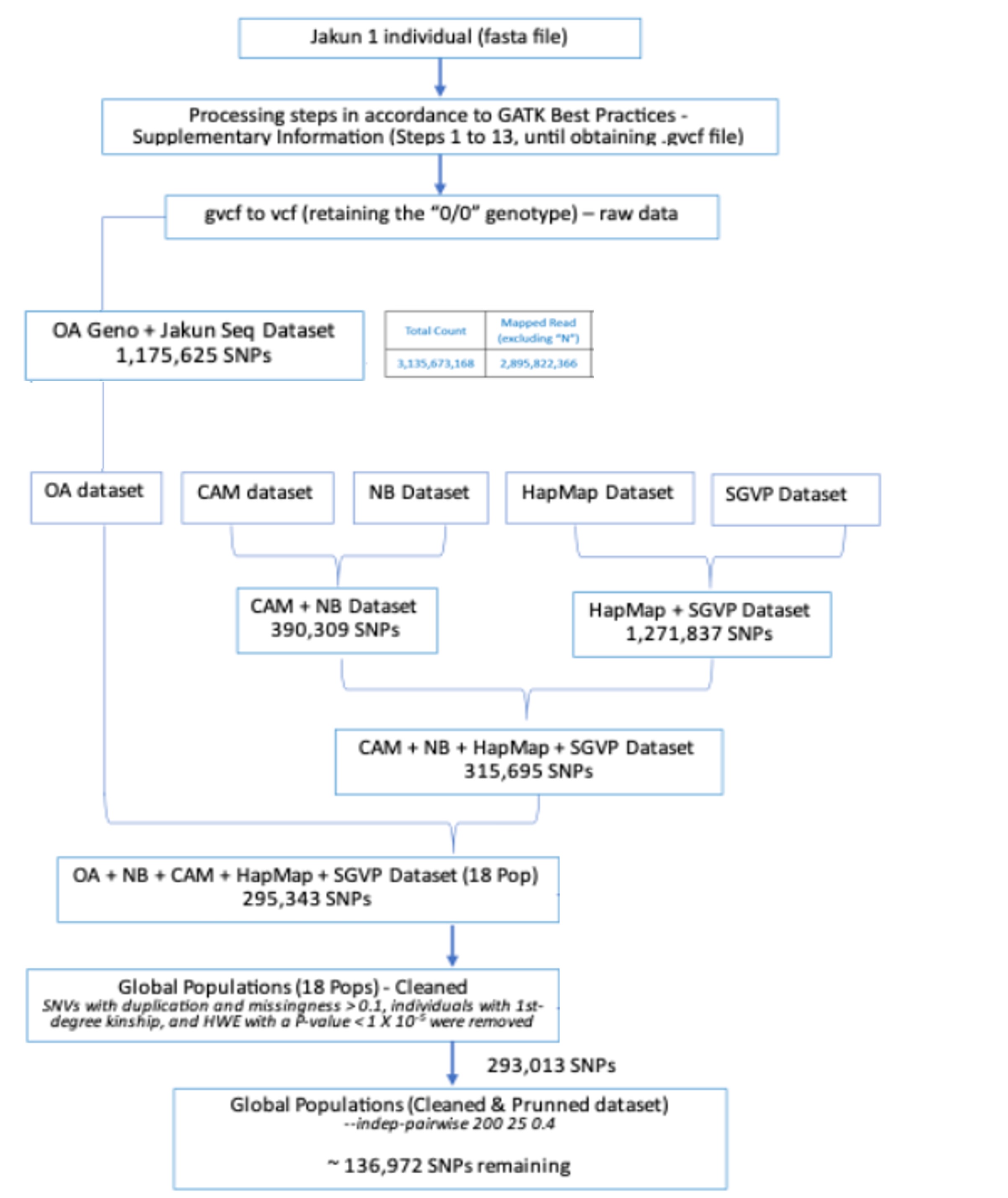

Supplement: Supplementary file 2 — Figure S1 [file 41439_2024_308_MOESM2_ESM.jpg]

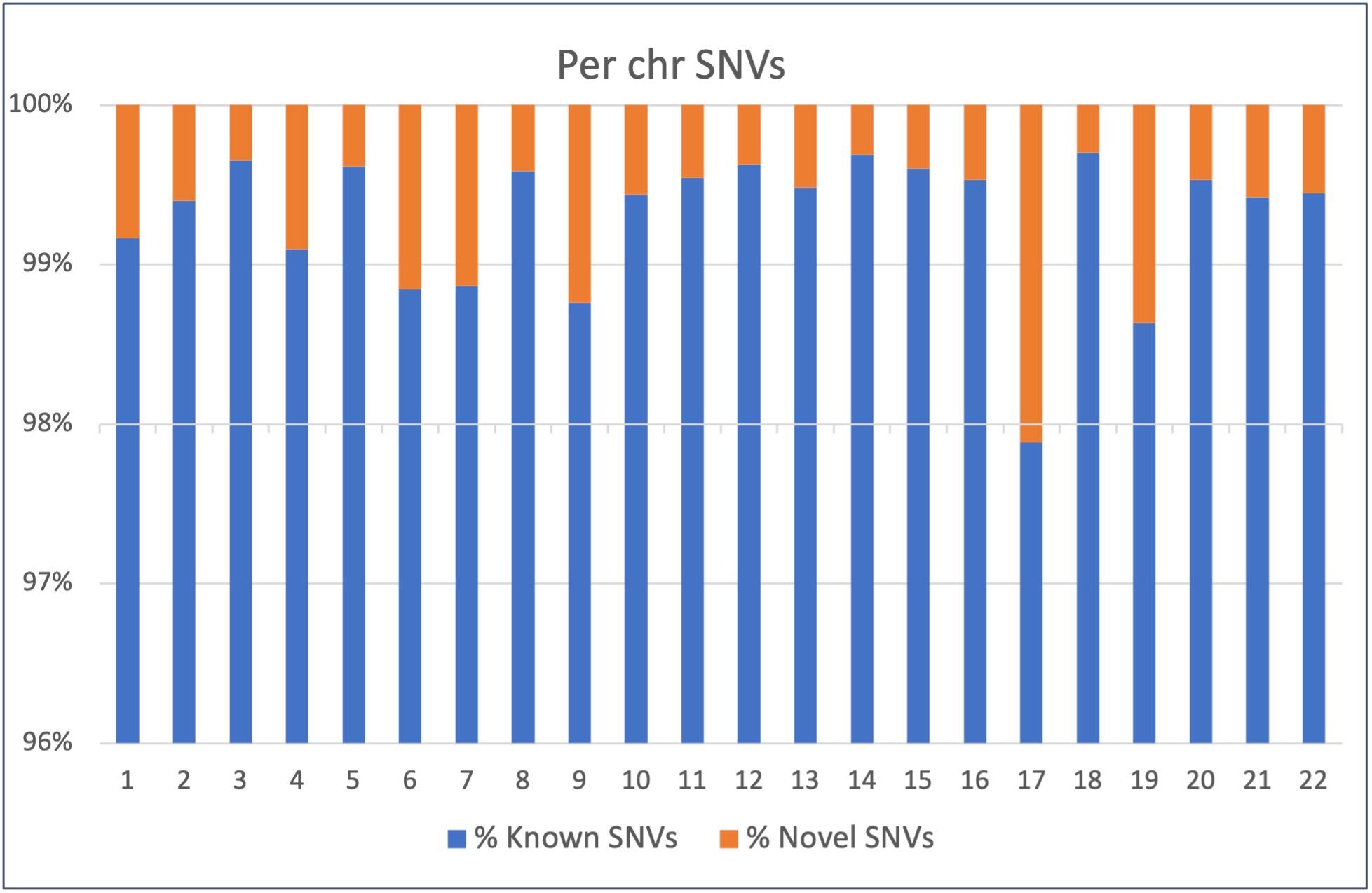

Supplement: Supplementary file 3 — Figure S2 [file 41439_2024_308_MOESM3_ESM.jpg]

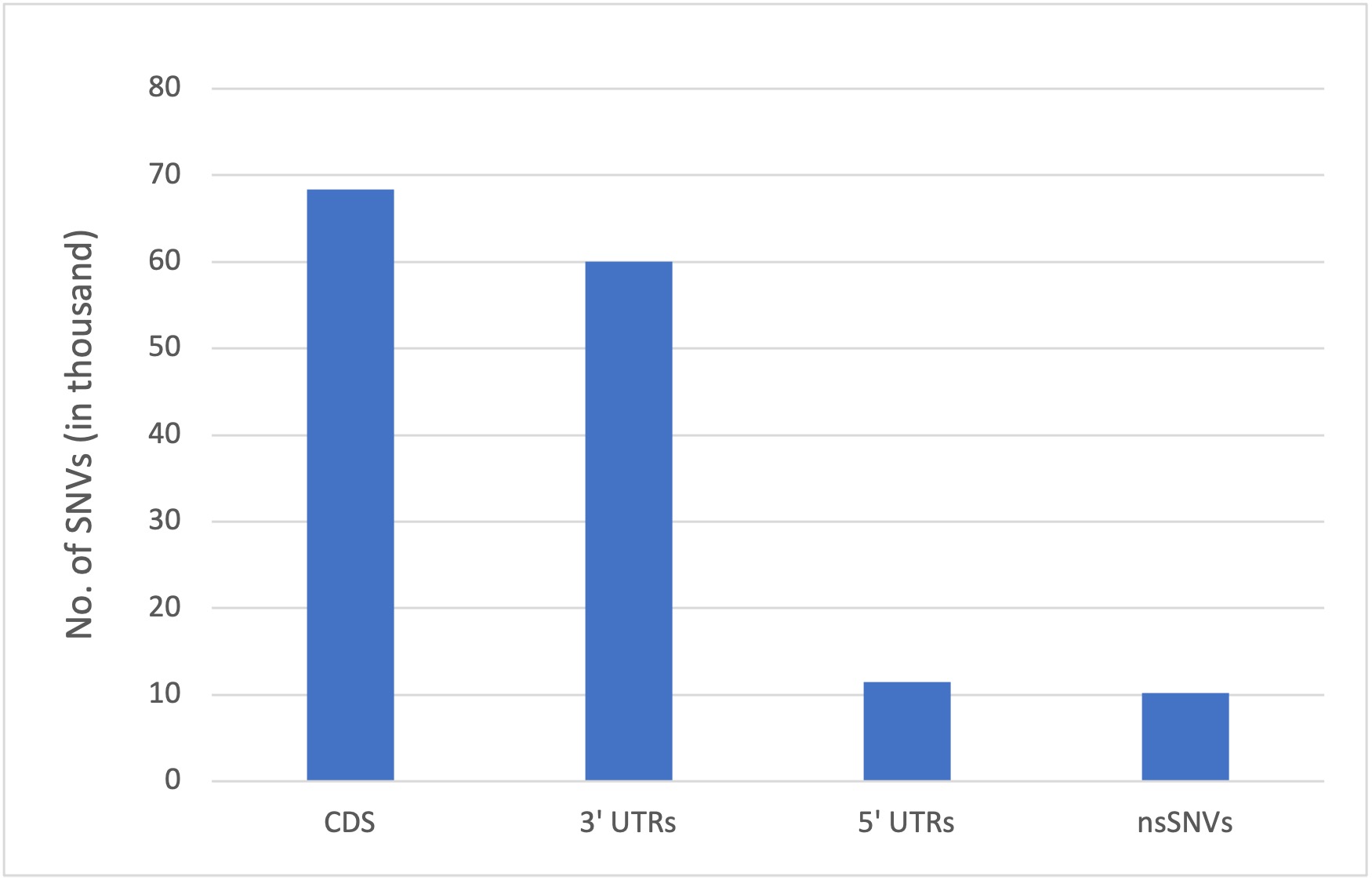

Supplement: Supplementary file 4 — Figure S3 [file 41439_2024_308_MOESM4_ESM.jpg]

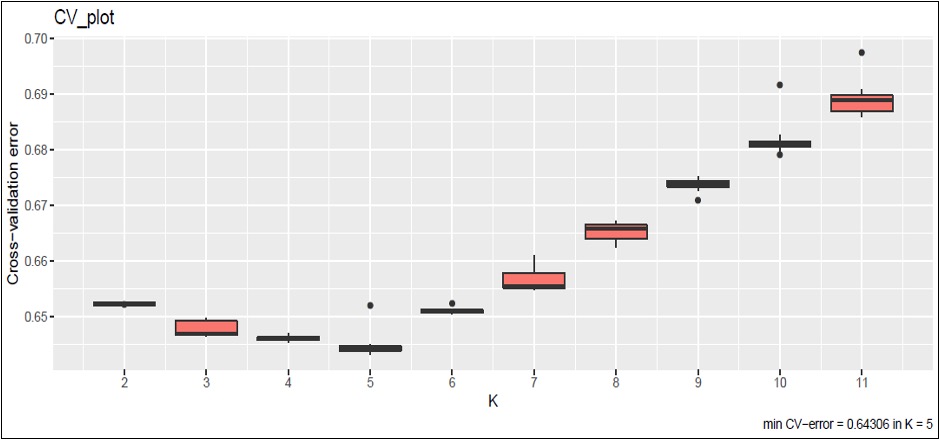

Supplement: Supplementary file 5 — Figure S4 [file 41439_2024_308_MOESM5_ESM.jpg]

K = 1

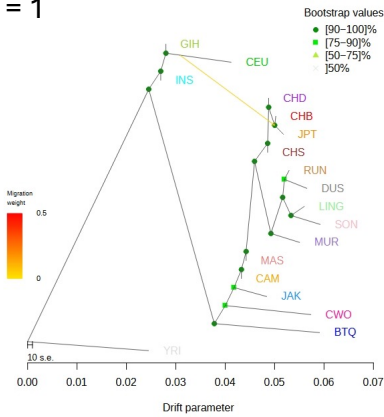

K = 2

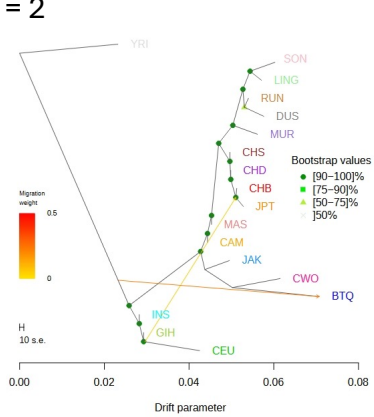

K = 3

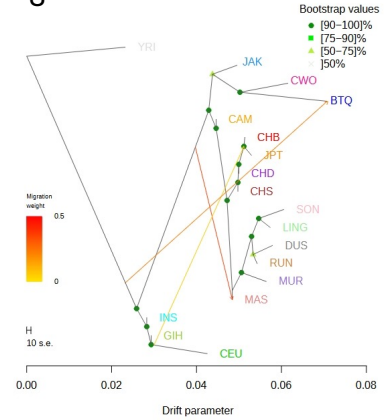

K = 4

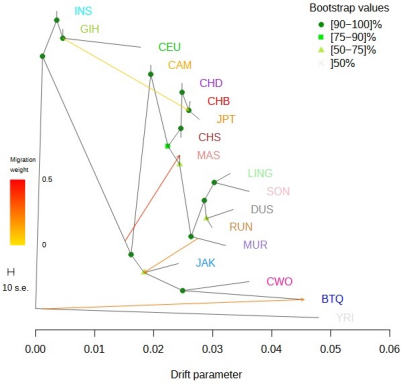

K = 5

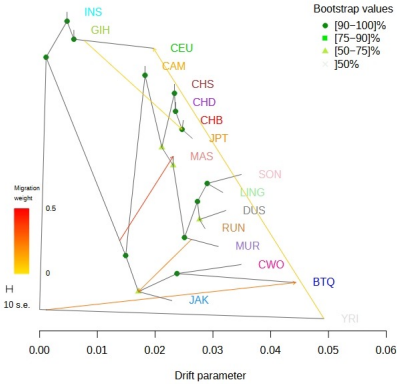

K = 6

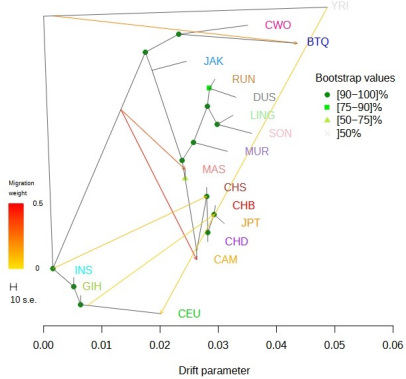

K = 7

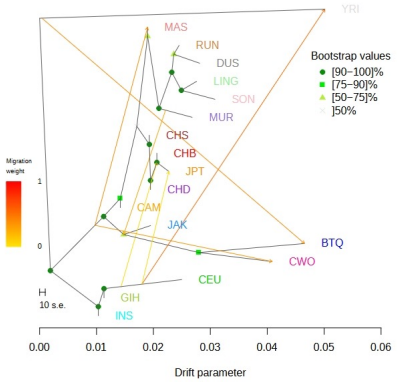

K = 8

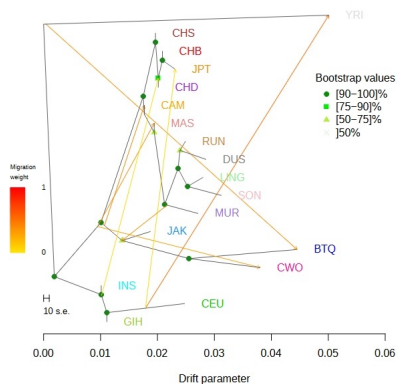

K = 9

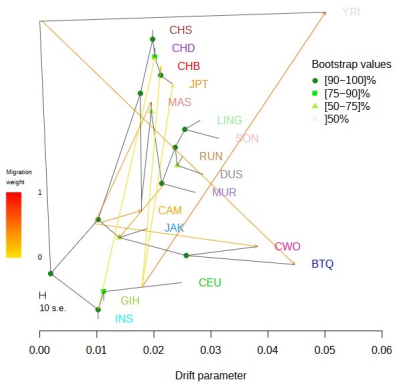

K = 10

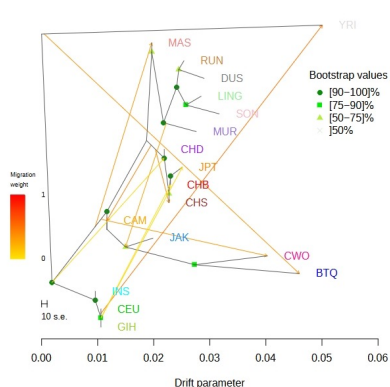

Supplement: Supplementary file 6 — Figure S5 [file 41439_2024_308_MOESM6_ESM.pdf]
