# Supplementary material for: High-coverage whole-genome sequencing of a Jakun individual from the “Orang Asli” Proto-Malay subtribe from Peninsular Malaysia
Source: Hum Genome Var. 2025 Jan 8;12:4. doi: 10.1038/s41439-024-00308-6 (PMC11707147; doi:10.1038/s41439-024-00308-6)
Supplement: Supplementary file 7 — Table S1 [file 41439_2024_308_MOESM7_ESM.docx]

**Table S1** Biological Materials (Sequencing and Genotyping Data) of Populations used in this study.

| **Population** | **No. Sample** | **Type of Data** | **Platform** | **Approx. No. of SNPs** | **Reference** |
| --- | --- | --- | --- | --- | --- |
| **In-house Server (Dell Precision Tower)** | | | | | |
| OA (Peninsular Malaysia) | | | | |  |
| - Jakun (Jakun_Seq) | 1 | Whole genome sequencing (fastq format) | Illumina HiSeq 2000 | 3,135,673,168 (.gvcf)  3,595,557 (.vcf) | Current study |
| - Bateq - CheWong - Jakun (Jakun_Geno) | 10  10  10 | *Genotyping | Illumina Human Omni 2.5 Array | 2.3 million | Aghakhanian et al., 2015 |
| North Borneo (Sabah) | | | |  |  |
| - Dusun - Lingkabau - Murut-Paluan - Rungus - Sonsogon | 10  10  10  10  10 | *Genotyping | Illumina Human Omni 2.5 Array | 2.3 million | Yew et al., 2018 |
| **Publicly Available Data** | | | | | |
| SGVP (Singapore Genome Variation Project) | | | | |  |
| - Singaporean Malay (SG_MAS) - Singaporean Chinese (SG_CHS) - Singaporean India (SG_INS) | 10  10  10 | *Genotyping | Affymetrix SNP6.0 Genotyping Chip and the Illumina 1M-single DNA Analysis BeadChip | 1.4 million | Teo et al., 2009 |
| - Cambodian (CAM) | 10 | *Genotyping | Human Origin | 660,388 | Patterson et. al., 2012 |
| HapMap 3 (Third phase of the International HapMap Project) | | | | |  |
| - CEU - CHB - CHD - JPT - GIH - YRI | 10  10  10  10  10  10 | *Genotyping | Affymetrix Human SNP Array 6.0 and the Illumina Human1M-single BeadChip | 1.4 million | International HapMap 3 Consortium, 2010 |

** File format acquired in plink binary format (.bed .bim and .fam)*
