# Supplementary material for: High-coverage whole-genome sequencing of a Jakun individual from the “Orang Asli” Proto-Malay subtribe from Peninsular Malaysia
Source: Hum Genome Var. 2025 Jan 8;12:4. doi: 10.1038/s41439-024-00308-6 (PMC11707147; doi:10.1038/s41439-024-00308-6)
Supplement: Supplementary file 8 — Table S2 [file 41439_2024_308_MOESM8_ESM.pdf]

Table S3: Percentage of Admixture Components from Global Populations (K = 5)

	K = 1	K = 2	K = 3	K = 4	K = 5
	Eur & India	East Asia	Yoruba	Austroasiatic / Negrito	North Borneo
Bateq (10)	2	3.1	0	93.2	1.3
CheWong (10)	0.9	7.7	0	80.7	10.4
Jakun_Seq (1)	7.4	48.1	0.9	20.3	23.3
Jakun_Geno (10)	4.5	38.6	2.4	33.7	20.8
Dusun (10)	0	6.3	0	0	93.5
Lingkabau (10)	0	4.9	0	0.5	94.2
Murut-P (10)	0	30.6	0	3.8	65.3
Rungus (10)	0	15.6	0	1.2	82.6
Sonsogon (10)	0	0	0	0	100
Cambodia (10)	9.2	53.3	3.3	14.6	19.7
SG_MAS (10)	9.3	47.7	1.1	11.9	30
SG_CHS (10)	0	87.7	0	1.2	11
CHB (10)	0.5	95.7	0	0	3.3
CHD (10)	0	89.8	0	1.1	8.8
Japanese (10)	1.2	98.5	0	0	0
SG_INS (10)	77.8	12.5	1	8.4	0
GIH (10)	81.1	10.4	0	7.6	0.7
CEU (10)	100	0	0	0	0
YRI (10)	0	0	100	0	0

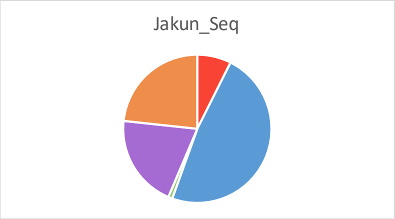

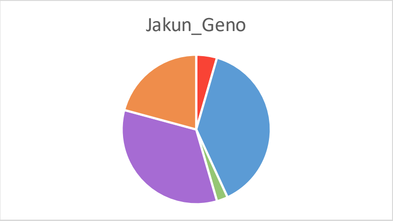

Table S3: Percentage of Admixture Components from Global Populations (K = 6)

	K = 1	K = 2	K = 3	K = 4	K = 5	K = 6
	Eur & India	East Asia	Yoruba	CheWong	North Borneo	Bateq
Bateq (10)	1.7	3.4	0	1.5	1.5	91.6
CheWong (10)	1.2	6.1	0	83.8	3.8	4.8
Jakun_Seq (1)	7.9	48.4	0.9	12.4	22.2	8.2
Jakun_Geno (10)	5.4	39.2	2.4	20.3	19.2	13.4
Dusun (10)	0	6.1	0	0	93.4	0
Lingkabau (10)	0	4.7	0	1	94	0
Murut-P (10)	0	30.6	0	3.6	64.8	0.7
Rungus (10)	0	15.4	0	1.5	82.3	0
Sonsogon (10)	0	0	0	0	100	0
Cambodia (10)	9.5	53.3	3.3	9.9	18.8	5.2
SG_MAS (10)	9.6	47.7	1.2	8.5	29.1	3.9
SG_CHS (10)	0	87.6	0	1.4	10.8	0
CHB (10)	0.5	95.8	0	0	3.3	0
CHD (10)	0	89.7	0	1.3	8.6	0
Japanese (10)	1.1	98.5	0	0	0	0
SG_INS (10)	77.8	12.8	1	2.7	0	5.5
GIH (10)	81.1	10.8	0	2	0.7	5.3
CEU (10)	0	100	0	0	0	0
YRI (10)	0	0	100	0	0	0

Table S3: Percentage of Admixture Components from Global Populations (K = 7)

	K = 1	K = 2	K = 3	K = 4	K = 5	K = 6	K = 7
	Eur & India	East Asia	Yoruba	CheWong	North Borneo	Bateq	Jakun
Bateq (10)	1.7	3.1	0	1.3	1.5	91.4	0.7
CheWong (10)	1.2	5.3	0	83.2	3.6	4.5	1.9
Jakun_Seq (1)	7.4	42.5	0	8.6	20.5	5.9	14.8
Jakun_Geno (10)	4.2	21.6	0.9	9.2	12.7	6.1	45.4
Dusun (10)	0	5.9	0	0	93.4	0	0
Lingkabau (10)	0	4.6	0	1	94	0	0
Murut-P (10)	0	29.4	0	2.9	64.6	0	2.4
Rungus (10)	0	15	0	1.3	82.2	0	0.6
Sonsogon (10)	0	0	0	0	100	0	0
Cambodia (10)	9.2	50.7	3.2	8.2	18.1	4.3	6.3
SG_MAS (10)	9.4	45.5	1.1	7	28.6	3.1	5.2
SG_CHS (10)	0	87.2	0	1.2	10.7	0	0.7
CHB (10)	0	95.7	0	0	3.2	0	0
CHD (10)	0	89.2	0	1	8.6	0	0.8
Japanese (10)	1	98.6	0	0	0	0	0
SG_INS (10)	77.7	11.9	1.1	2.1	0	5.2	1.9
GIH (10)	81	9.9	0	1.5	0.6	5	1.7
CEU (10)	100	0	0	0	0	0	0
YRI (10)	0	0	100	0	0	0	0
