# Supplementary material for: High-coverage whole-genome sequencing of a Jakun individual from the “Orang Asli” Proto-Malay subtribe from Peninsular Malaysia
Source: Hum Genome Var. 2025 Jan 8;12:4. doi: 10.1038/s41439-024-00308-6 (PMC11707147; doi:10.1038/s41439-024-00308-6)
Supplement: Supplementary file 9 — Tabls S3 [file 41439_2024_308_MOESM9_ESM.pdf]

Table S3a F3 and F4 statistics of the Jakun_Seq+Geno and its neighbouring populations

Pop (X)	Pop (W)	Pop (Y)	Pop (Z)	F4 Stat	SD	|Z-score|
Jakun_Seq+Geno	Bateq	Cambodia	YRI	0.004413822	2.42E-04	18.2540927
Jakun_Seq+Geno	Bateq	CEU	YRI	0.000330835	2.19E-04	1.510245894
Jakun_Seq+Geno	Bateq	CHB	YRI	0.004479028	2.52E-04	17.76447066
Jakun_Seq+Geno	Bateq	CHD	YRI	0.004679229	2.54E-04	18.38724202
Jakun_Seq+Geno	Bateq	CheWong	YRI	-0.000210759	2.90E-04	0.726566291
Jakun_Seq+Geno	Bateq	Dusun	YRI	0.005354943	2.68E-04	20.00121112
Jakun_Seq+Geno	Bateq	GIH	YRI	0.000332448	2.10E-04	1.582399199
Jakun_Seq+Geno	Bateq	JPT	YRI	0.004102932	2.52E-04	16.31332022
Jakun_Seq+Geno	Bateq	Lingkabau	YRI	0.005296966	2.72E-04	19.50625461
Jakun_Seq+Geno	Bateq	Murut-P	YRI	0.00525052	2.69E-04	19.5252189
Jakun_Seq+Geno	Bateq	Rungus	YRI	0.005326642	2.66E-04	20.03429573
Jakun_Seq+Geno	Bateq	SG_CHS	YRI	0.00482074	2.55E-04	18.92673741
Jakun_Seq+Geno	Bateq	SG_INS	YRI	0.000407588	2.07E-04	1.964826426
Jakun_Seq+Geno	Bateq	SG_MAS	YRI	0.004571699	2.43E-04	18.81505404
Jakun_Seq+Geno	Bateq	Sonsogon	YRI	0.005118246	2.68E-04	19.07640367
Jakun_Seq+Geno	CheWong	Dusun	YRI	0.001158438	2.49E-04	4.64381705
Jakun_Seq+Geno	CheWong	GIH	YRI	-0.000121318	1.92E-04	0.630241997
Jakun_Seq+Geno	CheWong	JPT	YRI	0.001064796	2.29E-04	4.656158779
Jakun_Seq+Geno	CheWong	Lingkabau	YRI	0.001037257	2.48E-04	4.174442015
Jakun_Seq+Geno	CheWong	Murut-P	YRI	0.001007895	2.44E-04	4.123658438
Jakun_Seq+Geno	CheWong	Rungus	YRI	0.001120705	2.45E-04	4.571343221
Jakun_Seq+Geno	CheWong	SG_INS	YRI	-0.000165714	1.94E-04	0.854613301
Jakun_Seq+Geno	CheWong	SG_MAS	YRI	0.000634661	2.24E-04	2.835911839
Jakun_Seq+Geno	CheWong	Sonsogon	YRI	0.001192373	2.46E-04	4.845359739
Jakun_Seq+Geno	Cambodia	CEU	YRI	-0.000134096	1.64E-04	0.818545502
Jakun_Seq+Geno	Cambodia	CHB	YRI	-0.001428068	1.90E-04	7.506570976
Jakun_Seq+Geno	Cambodia	CHD	YRI	-0.00134949	1.89E-04	7.142809823
Jakun_Seq+Geno	Cambodia	CheWong	YRI	0.001866225	2.02E-04	9.237505528
Jakun_Seq+Geno	Cambodia	Dusun	YRI	-0.000224991	1.97E-04	1.140797167
Jakun_Seq+Geno	Cambodia	GIH	YRI	-2.33586E-05	1.54E-04	0.152009237
Jakun_Seq+Geno	Cambodia	JPT	YRI	-0.001403542	1.87E-04	7.49458113
Jakun_Seq+Geno	Cambodia	Lingkabau	YRI	-0.000299433	2.00E-04	1.49534496
Jakun_Seq+Geno	Cambodia	Murut-P	YRI	-0.000195524	1.99E-04	0.983109517

Jakun_Seq+Geno	Cambodia	Rungus	YRI	-0.000230254	2.01E-04	1.146700585
Jakun_Seq+Geno	Cambodia	SG_CHS	YRI	-0.001403378	1.92E-04	7.29322098
Jakun_Seq+Geno	Cambodia	SG_INS	YRI	4.72997E-07	1.54E-04	0.00306471
Jakun_Seq+Geno	Cambodia	SG_MAS	YRI	-0.000373125	1.82E-04	2.053409165
Jakun_Seq+Geno	Cambodia	Sonsogon	YRI	-0.000198115	1.99E-04	0.994230773
Jakun_Seq+Geno	SG_MAS	JPT	YRI	-0.001882864	1.86E-04	10.11498202
Jakun_Seq+Geno	SG_MAS	Rungus	YRI	-0.001779961	2.00E-04	8.877923709
Jakun_Seq+Geno	SG_MAS	Lingkabau	YRI	-0.001745753	2.05E-04	8.527909528
Jakun_Seq+Geno	SG_MAS	Sonsogon	YRI	-0.001706757	2.06E-04	8.303054858
Jakun_Seq+Geno	SG_MAS	Murut-P	YRI	-0.001697569	2.01E-04	8.450469424
Jakun_Seq+Geno	Dusun	GIH	YRI	-0.000466687	1.86E-04	2.50903367
Jakun_Seq+Geno	Dusun	JPT	YRI	-0.004778145	2.25E-04	21.22484301
Jakun_Seq+Geno	Dusun	Lingkabau	YRI	-0.009740429	2.47E-04	39.50763277
Jakun_Seq+Geno	Dusun	Murut-P	YRI	-0.007453596	2.41E-04	30.96706796
Jakun_Seq+Geno	Dusun	Rungus	YRI	-0.010232752	2.44E-04	41.9389752
Jakun_Seq+Geno	Dusun	SG_INS	YRI	-0.000448357	1.85E-04	2.428209004
Jakun_Seq+Geno	Dusun	SG_MAS	YRI	-0.003784219	2.18E-04	17.34959192
Jakun_Seq+Geno	Dusun	Sonsogon	YRI	-0.01032029	2.50E-04	41.35293527
Jakun_Seq+Geno	Lingkabau	Sonsogon	YRI	-0.01148025	2.44E-04	47.10340307
Jakun_Seq+Geno	Lingkabau	Rungus	YRI	-0.009568619	2.37E-04	40.40830938
Jakun_Seq+Geno	Lingkabau	Murut-P	YRI	-0.007285578	2.38E-04	30.66773917
Jakun_Seq+Geno	Lingkabau	JPT	YRI	-0.004749686	2.21E-04	21.47791148
Jakun_Seq+Geno	Lingkabau	SG_MAS	YRI	-0.003837635	2.14E-04	17.97009505
Jakun_Seq+Geno	Murut-P	Rungus	YRI	-0.007473024	2.34E-04	31.88245953
Jakun_Seq+Geno	Murut-P	Sonsogon	YRI	-0.007338218	2.36E-04	31.08007797
Jakun_Seq+Geno	Murut-P	Lingkabau	YRI	-0.007256248	2.38E-04	30.51342246
Jakun_Seq+Geno	Murut-P	JPT	YRI	-0.004572979	2.17E-04	21.05814447
Jakun_Seq+Geno	Murut-P	SG_MAS	YRI	-0.003760121	2.11E-04	17.82683509
Jakun_Seq+Geno	Rungus	Sonsogon	YRI	-0.009823298	2.41E-04	40.77385802
Jakun_Seq+Geno	Rungus	Lingkabau	YRI	-0.009629448	2.40E-04	40.18880543
Jakun_Seq+Geno	Rungus	Murut-P	YRI	-0.007563184	2.30E-04	32.86519923
Jakun_Seq+Geno	Rungus	JPT	YRI	-0.004845928	2.14E-04	22.61085931
Jakun_Seq+Geno	Rungus	SG_MAS	YRI	-0.003932673	2.09E-04	18.80554165
Jakun_Seq+Geno	Sonsogon	Lingkabau	YRI	-0.011397771	2.48E-04	45.90131204
Jakun_Seq+Geno	Sonsogon	Rungus	YRI	-0.00967999	2.43E-04	39.81071258
Jakun_Seq+Geno	Sonsogon	Murut-P	YRI	-0.007285069	2.37E-04	30.75374118
Jakun_Seq+Geno	Sonsogon	JPT	YRI	-0.004576485	2.26E-04	20.26541635

Jakun_Seq+Geno	Sonsogon	SG_MAS	YRI	-0.00371616	2.19E-04	16.97558
Jakun_Seq+Geno	CHB	CHD	YRI	-0.006413317	2.08E-04	30.81719865
Jakun_Seq+Geno	CHB	CheWong	YRI	0.002222691	2.19E-04	10.15472729
Jakun_Seq+Geno	CHB	Dusun	YRI	-0.002753796	2.19E-04	12.55687381
Jakun_Seq+Geno	CHB	GIH	YRI	-0.000584329	1.72E-04	3.400255826
Jakun_Seq+Geno	CHB	JPT	YRI	-0.007180148	2.06E-04	34.79727165
Jakun_Seq+Geno	CHB	Lingkabau	YRI	-0.00269206	2.21E-04	12.17141392
Jakun_Seq+Geno	CHB	Murut-P	YRI	-0.002462668	2.16E-04	11.40733929
Jakun_Seq+Geno	CHB	Rungus	YRI	-0.002700001	2.18E-04	12.39544431
Jakun_Seq+Geno	CHB	SG_CHS	YRI	-0.006326385	2.13E-04	29.63723029
Jakun_Seq+Geno	CHB	SG_INS	YRI	-0.000529896	1.70E-04	3.110963938
Jakun_Seq+Geno	CHB	SG_MAS	YRI	-0.001804146	1.97E-04	9.160322241
Jakun_Seq+Geno	CHB	Sonsogon	YRI	-0.002558704	2.22E-04	11.52751456
Jakun_Seq+Geno	CHD	CheWong	YRI	0.001420291	2.24E-04	6.340491163
Jakun_Seq+Geno	CHD	Dusun	YRI	-0.003493699	2.19E-04	15.93847491
Jakun_Seq+Geno	CHD	GIH	YRI	-0.000625539	1.73E-04	3.618766508
Jakun_Seq+Geno	CHD	JPT	YRI	-0.006963729	2.04E-04	34.07139299
Jakun_Seq+Geno	CHD	Lingkabau	YRI	-0.003329994	2.21E-04	15.04982927
Jakun_Seq+Geno	CHD	Murut-P	YRI	-0.003343437	2.19E-04	15.29326744
Jakun_Seq+Geno	CHD	Rungus	YRI	-0.003409021	2.20E-04	15.48365891
Jakun_Seq+Geno	CHD	SG_CHS	YRI	-0.006620482	2.13E-04	31.04184821
Jakun_Seq+Geno	CHD	SG_INS	YRI	-0.00063545	1.70E-04	3.727295984
Jakun_Seq+Geno	CHD	SG_MAS	YRI	-0.002426352	1.99E-04	12.16359602
Jakun_Seq+Geno	CHD	Sonsogon	YRI	-0.003357209	2.22E-04	15.10154055
Jakun_Seq+Geno	SG_CHS	CheWong	YRI	0.001407004	2.22E-04	6.333797897
Jakun_Seq+Geno	SG_CHS	Dusun	YRI	-0.003753203	2.22E-04	16.90520432
Jakun_Seq+Geno	SG_CHS	GIH	YRI	-0.000424643	1.70E-04	2.497605721
Jakun_Seq+Geno	SG_CHS	JPT	YRI	-0.006896209	2.05E-04	33.57981977
Jakun_Seq+Geno	SG_CHS	Lingkabau	YRI	-0.003681801	2.24E-04	16.46111802
Jakun_Seq+Geno	SG_CHS	Murut-P	YRI	-0.003459814	2.22E-04	15.61668023
Jakun_Seq+Geno	SG_CHS	Rungus	YRI	-0.003679192	2.22E-04	16.58484937
Jakun_Seq+Geno	SG_CHS	SG_INS	YRI	-0.000477861	1.72E-04	2.773340629
Jakun_Seq+Geno	SG_CHS	SG_MAS	YRI	-0.002644853	2.00E-04	13.24469107
Jakun_Seq+Geno	SG_CHS	Sonsogon	YRI	-0.003633914	2.21E-04	16.4258126
Jakun_Seq+Geno	JPT	Rungus	YRI	-0.001844234	2.27E-04	8.108397575
Jakun_Seq+Geno	JPT	Lingkabau	YRI	-0.001808821	2.36E-04	7.66156306
Jakun_Seq+Geno	JPT	Sonsogon	YRI	-0.0017181	2.36E-04	7.292486987

Jakun_Seq+Geno	JPT	Murut-P	YRI	-0.001661445	2.29E-04	7.262506894
Jakun_Seq+Geno	JPT	SG_MAS	YRI	-0.001033882	2.06E-04	5.009217889
Jakun_Seq+Geno	SG_INS	JPT	YRI	0.013655203	2.63E-04	51.89835213
Jakun_Seq+Geno	SG_INS	Lingkabau	YRI	0.016578395	2.77E-04	59.94431237
Jakun_Seq+Geno	SG_INS	SG_MAS	YRI	0.014389568	2.57E-04	56.09304921
Jakun_Seq+Geno	SG_INS	Murut-P	YRI	0.016595797	2.68E-04	61.86059785
Jakun_Seq+Geno	SG_INS	Rungus	YRI	0.016624171	2.72E-04	61.22451808
Jakun_Seq+Geno	SG_INS	Sonsogon	YRI	0.016597582	2.83E-04	58.58549973
Jakun_Seq+Geno	GIH	JPT	YRI	0.01377153	0.000270444	50.92189749
Jakun_Seq+Geno	GIH	Lingkabau	YRI	0.01660628	0.000282984	58.68278735
Jakun_Seq+Geno	GIH	Murut-P	YRI	0.016662108	0.000275429	60.49507576
Jakun_Seq+Geno	GIH	Rungus	YRI	0.016627327	0.000276586	60.11625575
Jakun_Seq+Geno	GIH	SG_INS	YRI	-0.003768288	0.000222943	16.90246129
Jakun_Seq+Geno	GIH	SG_MAS	YRI	0.014487387	0.000265073	54.65424123
Jakun_Seq+Geno	GIH	Sonsogon	YRI	0.016748818	0.000290777	57.60020189
Jakun_Seq+Geno	CEU	CHB	YRI	0.018164523	3.29E-04	55.18673277
Jakun_Seq+Geno	CEU	CHD	YRI	0.01893759	3.27E-04	57.901898
Jakun_Seq+Geno	CEU	CheWong	YRI	0.019964075	3.38E-04	59.02594541
Jakun_Seq+Geno	CEU	Dusun	YRI	0.020467008	3.37E-04	60.76993267
Jakun_Seq+Geno	CEU	GIH	YRI	-0.00127334	2.65E-04	-4.813175777
Jakun_Seq+Geno	CEU	JPT	YRI	0.017446381	3.24E-04	53.78212737
Jakun_Seq+Geno	CEU	Lingkabau	YRI	0.020485021	3.35E-04	61.23185017
Jakun_Seq+Geno	CEU	Murut-P	YRI	0.020562564	3.30E-04	62.39605648
Jakun_Seq+Geno	CEU	Rungus	YRI	0.020513649	3.29E-04	62.29090621
Jakun_Seq+Geno	CEU	SG_CHS	YRI	0.019275512	3.23E-04	59.59461698
Jakun_Seq+Geno	CEU	SG_INS	YRI	-0.000481285	2.68E-04	-1.795425447
Jakun_Seq+Geno	CEU	SG_MAS	YRI	0.01829026	3.17E-04	57.66598937
Jakun_Seq+Geno	CEU	Sonsogon	YRI	0.02051565	3.38E-04	60.60853253

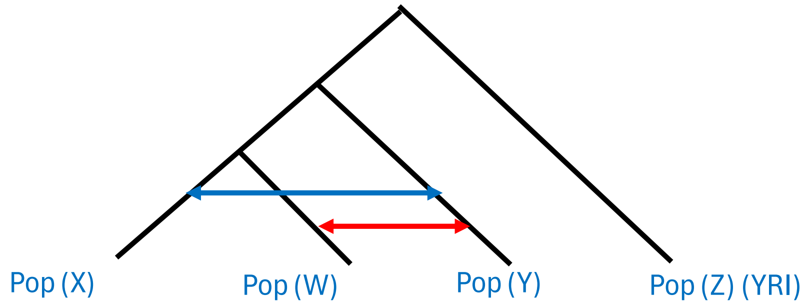

Note:

The F4 statistic detects gene flow between populations based on shared derived alleles.

A non-zero F4 value suggests potential gene flow.

The Z-score, calculated by dividing the F4 value by its standard error, indicates the significance of the F4 value.

An absolute Z-score (typically $|Z| > 2$) denotes statistical significance, implying evidence for gene flow.

Table S3b F3 and F4 statistics of the Jakun_Seq+Geno and its neighbouring populations

(A)	(B)	(C)	F3 Stat	SD	Z-score
Bateq	Cambodia	Jakun_Seq+Geno	0.024464733	3.47E-04	70.53395289
Bateq	CEU	Jakun_Seq+Geno	0.020381745	3.34E-04	61.08335515
Bateq	CHB	Jakun_Seq+Geno	0.024529938	3.45E-04	71.12441525
Bateq	CHD	Jakun_Seq+Geno	0.024730139	3.48E-04	71.04619078
Bateq	CheWong	Jakun_Seq+Geno	0.019840151	3.38E-04	58.68486607
Bateq	Dusun	Jakun_Seq+Geno	0.025405854	3.61E-04	70.31658601
Bateq	GIH	Jakun_Seq+Geno	0.020383358	3.21E-04	63.55925351
Bateq	Jakun_Seq+Geno	YRI	0.02005091	3.55E-04	56.4851733
Bateq	Jakun_Seq+Geno	JPT	0.024153843	3.49E-04	69.2329012
Bateq	Jakun_Seq+Geno	Sonsogon	0.025169156	3.61E-04	69.69313888
Bateq	Jakun_Seq+Geno	Lingkabau	0.025347877	3.63E-04	69.78563198
Bateq	Jakun_Seq+Geno	Murut-P	0.02530143	3.62E-04	69.81804384
Bateq	Jakun_Seq+Geno	Rungus	0.025377553	3.63E-04	69.83018082
Bateq	Jakun_Seq+Geno	SG_MAS	0.02462261	3.40E-04	72.33794325
Bateq	SG_CHS	Jakun_Seq+Geno	0.024871651	3.51E-04	70.92493355
Bateq	SG_INS	Jakun_Seq+Geno	0.020458498	3.21E-04	63.64250011
Cambodia	CEU	Jakun_Seq+Geno	1.54E-04	1.65E-04	0.937487115
Cambodia	CHB	Jakun_Seq+Geno	-0.001139503	1.07E-04	10.65732752
Cambodia	CHD	Jakun_Seq+Geno	-0.001060926	1.04E-04	10.15819275
Cambodia	CheWong	Jakun_Seq+Geno	0.002154789	1.45E-04	14.85144921
Cambodia	Dusun	Jakun_Seq+Geno	6.36E-05	1.16E-04	0.549735452
Cambodia	GIH	Jakun_Seq+Geno	2.65E-04	1.47E-04	1.803374109
Cambodia	Jakun_Seq+Geno	JPT	-0.001114977	1.11E-04	10.01008194
Cambodia	Jakun_Seq+Geno	SG_MAS	-8.46E-05	1.03E-04	0.821581992
Cambodia	Jakun_Seq+Geno	Lingkabau	-1.09E-05	1.20E-04	0.090404653
Cambodia	Jakun_Seq+Geno	Rungus	5.83E-05	1.14E-04	0.513486661
Cambodia	Jakun_Seq+Geno	Sonsogon	9.04E-05	1.22E-04	0.742945837
Cambodia	Jakun_Seq+Geno	Murut-P	9.30E-05	1.15E-04	0.80560072
Cambodia	Jakun_Seq+Geno	YRI	2.89E-04	1.85E-04	1.559967946
Cambodia	Jakun_Seq+Geno	Bateq	0.002537926	1.62E-04	15.65825107
Cambodia	SG_CHS	Jakun_Seq+Geno	-0.001114813	1.03E-04	10.79126906
Cambodia	SG_INS	Jakun_Seq+Geno	2.89E-04	1.43E-04	2.019828986
CEU	CHB	Jakun_Seq+Geno	0.032852819	3.36E-04	97.74537288
CEU	CHD	Jakun_Seq+Geno	0.033625886	3.32E-04	101.3698483
CEU	CheWong	Jakun_Seq+Geno	0.03465237	3.47E-04	99.7980978
CEU	Dusun	Jakun_Seq+Geno	0.035155303	3.45E-04	101.9559905
CEU	GIH	Jakun_Seq+Geno	0.013414955	2.12E-04	63.1946089

CEU	Jakun_Seq+Geno	YRI	0.014688296	2.98E-04	49.36654361
CEU	Jakun_Seq+Geno	Bateq	0.031486601	3.49E-04	90.31667408
CEU	Jakun_Seq+Geno	JPT	0.032134676	3.33E-04	96.50668915
CEU	Jakun_Seq+Geno	Sonsogon	0.035203945	3.50E-04	100.4486932
CEU	Jakun_Seq+Geno	SG_MAS	0.032978555	3.25E-04	101.4067859
CEU	Jakun_Seq+Geno	Rungus	0.035201945	3.47E-04	101.4138201
CEU	Jakun_Seq+Geno	Lingkabau	0.035173316	3.45E-04	101.9251983
CEU	Jakun_Seq+Geno	Cambodia	0.033186132	3.26E-04	101.9392342
CEU	Jakun_Seq+Geno	Murut-P	0.03525086	3.40E-04	103.7103294
CEU	SG_CHS	Jakun_Seq+Geno	0.033963807	3.37E-04	100.8751092
CEU	SG_INS	Jakun_Seq+Geno	0.014207011	2.25E-04	63.03211143
CHB	CHD	Jakun_Seq+Geno	8.09E-04	1.08E-04	7.470343042
CHB	CheWong	Jakun_Seq+Geno	0.009444677	1.82E-04	51.84717458
CHB	Dusun	Jakun_Seq+Geno	0.004468189	1.37E-04	32.4999067
CHB	GIH	Jakun_Seq+Geno	0.006637657	1.75E-04	37.99713231
CHB	Jakun_Seq+Geno	JPT	4.18E-05	1.06E-04	0.396446736
CHB	Jakun_Seq+Geno	Sonsogon	0.004663281	1.53E-04	30.55235865
CHB	Jakun_Seq+Geno	Rungus	0.004521985	1.43E-04	31.58700221
CHB	Jakun_Seq+Geno	Lingkabau	0.004529926	1.43E-04	31.66609045
CHB	Jakun_Seq+Geno	YRI	0.007221986	2.18E-04	33.20002847
CHB	Jakun_Seq+Geno	Murut-P	0.004759318	1.43E-04	33.29433821
CHB	Jakun_Seq+Geno	CEU	0.006527101	1.93E-04	33.8057438
CHB	Jakun_Seq+Geno	SG_MAS	0.005417839	1.29E-04	41.96387728
CHB	Jakun_Seq+Geno	Cambodia	0.005566442	1.31E-04	42.40494029
CHB	Jakun_Seq+Geno	Bateq	0.009309076	2.03E-04	45.90680068
CHB	SG_CHS	Jakun_Seq+Geno	8.96E-04	1.08E-04	8.301249152
CHB	SG_INS	Jakun_Seq+Geno	0.00669209	1.70E-04	39.42366583
CHD	CheWong	Jakun_Seq+Geno	0.008147947	1.78E-04	45.7054345
CHD	Dusun	Jakun_Seq+Geno	0.003233957	1.33E-04	24.40445408
CHD	GIH	Jakun_Seq+Geno	0.006102117	1.72E-04	35.47089266
CHD	Jakun_Seq+Geno	CHB	-4.22E-04	1.04E-04	4.041066607
CHD	Jakun_Seq+Geno	JPT	-2.36E-04	1.10E-04	2.152205739
CHD	Jakun_Seq+Geno	Sonsogon	0.003370447	1.46E-04	23.10279761
CHD	Jakun_Seq+Geno	Lingkabau	0.003397662	1.38E-04	24.53194672
CHD	Jakun_Seq+Geno	Rungus	0.003318635	1.35E-04	24.65657446
CHD	Jakun_Seq+Geno	Murut-P	0.003384219	1.36E-04	24.84116012
CHD	Jakun_Seq+Geno	YRI	0.006727656	2.20E-04	30.54586211
CHD	Jakun_Seq+Geno	CEU	0.006069779	1.93E-04	31.42456863
CHD	Jakun_Seq+Geno	SG_MAS	0.004301304	1.24E-04	34.61483845
CHD	Jakun_Seq+Geno	Cambodia	0.00441463	1.22E-04	36.09066106
CHD	Jakun_Seq+Geno	Bateq	0.008278888	1.94E-04	42.74315428

CHD	SG_CHS	Jakun_Seq+Geno	1.07E-04	1.00E-04	1.071052038
CHD	SG_INS	Jakun_Seq+Geno	0.006092206	1.71E-04	35.52688577
CheWong	Dusun	Jakun_Seq+Geno	0.018622528	2.88E-04	64.73627827
CheWong	GIH	Jakun_Seq+Geno	0.017342772	2.87E-04	60.33254799
CheWong	Jakun_Seq+Geno	Bateq	0.013709216	3.00E-04	45.65501702
CheWong	Jakun_Seq+Geno	YRI	0.01746409	3.14E-04	55.66797826
CheWong	Jakun_Seq+Geno	CEU	0.017416579	3.00E-04	57.96244279
CheWong	Jakun_Seq+Geno	Lingkabau	0.018501347	2.90E-04	63.75961616
CheWong	Jakun_Seq+Geno	Murut-P	0.018471985	2.87E-04	64.32657588
CheWong	Jakun_Seq+Geno	Rungus	0.018584794	2.89E-04	64.35449258
CheWong	Jakun_Seq+Geno	Sonsogon	0.018656463	2.89E-04	64.56239832
CheWong	Jakun_Seq+Geno	JPT	0.018528886	2.87E-04	64.58807031
CheWong	Jakun_Seq+Geno	CHD	0.018468262	2.84E-04	65.0208246
CheWong	Jakun_Seq+Geno	Cambodia	0.017950661	2.75E-04	65.32279767
CheWong	Jakun_Seq+Geno	SG_MAS	0.018098751	2.77E-04	65.3511541
CheWong	Jakun_Seq+Geno	CHB	0.018534604	2.83E-04	65.49048064
CheWong	Jakun_Seq+Geno	SG_CHS	0.01854106	2.81E-04	65.96636154
CheWong	SG_INS	Jakun_Seq+Geno	0.017298375	2.88E-04	60.00872982
Dusun	GIH	Jakun_Seq+Geno	0.014310176	2.28E-04	62.78986106
Dusun	Jakun_Seq+Geno	Sonsogon	0.004456573	1.76E-04	25.27363067
Dusun	Jakun_Seq+Geno	Rungus	0.004544111	1.63E-04	27.91152775
Dusun	Jakun_Seq+Geno	Lingkabau	0.005036434	1.73E-04	29.10535302
Dusun	Jakun_Seq+Geno	Murut-P	0.007323267	1.80E-04	40.79244091
Dusun	Jakun_Seq+Geno	CHD	0.009871492	1.94E-04	51.01231527
Dusun	Jakun_Seq+Geno	SG_CHS	0.009698072	1.89E-04	51.36437341
Dusun	Jakun_Seq+Geno	CHB	0.009875335	1.91E-04	51.58403848
Dusun	Jakun_Seq+Geno	JPT	0.009998718	1.94E-04	51.62607982
Dusun	Jakun_Seq+Geno	YRI	0.014776863	2.68E-04	55.15499797
Dusun	Jakun_Seq+Geno	SG_MAS	0.010992644	1.91E-04	57.46229496
Dusun	Jakun_Seq+Geno	CEU	0.014236732	2.45E-04	58.21840869
Dusun	Jakun_Seq+Geno	Cambodia	0.012176665	2.00E-04	60.97020104
Dusun	Jakun_Seq+Geno	CheWong	0.014939747	2.38E-04	62.72695929
Dusun	Jakun_Seq+Geno	Bateq	0.015592138	2.43E-04	64.22501148
Dusun	SG_INS	Jakun_Seq+Geno	0.014328506	2.31E-04	61.92432229
GIH	Jakun_Seq+Geno	CEU	-2.54E-04	1.57E-04	1.611884517
GIH	Jakun_Seq+Geno	YRI	0.004877437	2.28E-04	21.36097621
GIH	Jakun_Seq+Geno	Bateq	0.017819592	2.55E-04	70.00708417
GIH	Jakun_Seq+Geno	JPT	0.018648967	2.32E-04	80.37547719
GIH	Jakun_Seq+Geno	CheWong	0.020909941	2.52E-04	82.94286047
GIH	Jakun_Seq+Geno	CHB	0.019294752	2.31E-04	83.3854156
GIH	Jakun_Seq+Geno	CHD	0.019989601	2.34E-04	85.30087052

GIH	Jakun_Seq+Geno	Sonsogon	0.021626255	2.51E-04	86.10163022
GIH	Jakun_Seq+Geno	SG_MAS	0.019364824	2.24E-04	86.55879248
GIH	Jakun_Seq+Geno	Lingkabau	0.021483718	2.47E-04	86.98165657
GIH	Jakun_Seq+Geno	SG_CHS	0.020276582	2.32E-04	87.26024353
GIH	Jakun_Seq+Geno	Cambodia	0.019628247	2.23E-04	87.98617346
GIH	Jakun_Seq+Geno	Dusun	0.021560125	2.44E-04	88.24723859
GIH	Jakun_Seq+Geno	Rungus	0.021504765	2.43E-04	88.49136215
GIH	Jakun_Seq+Geno	Murut-P	0.021539545	2.41E-04	89.39549441
GIH	SG_INS	Jakun_Seq+Geno	0.001109149	1.24E-04	8.958774137
Jakun_Seq+Geno	Bateq	Dusun	0.005216927	1.99E-04	26.23194515
Jakun_Seq+Geno	Bateq	SG_CHS	0.00575113	1.82E-04	31.58361381
Jakun_Seq+Geno	Bateq	CHD	0.005892641	1.84E-04	31.98103051
Jakun_Seq+Geno	Bateq	CHB	0.006092842	1.85E-04	32.98893885
Jakun_Seq+Geno	Bateq	Cambodia	0.006158048	1.72E-04	35.74773836
Jakun_Seq+Geno	Bateq	CEU	0.010241035	2.53E-04	40.45210523
Jakun_Seq+Geno	Bateq	CheWong	0.010782629	2.60E-04	41.47193706
Jakun_Seq+Geno	Bateq	SG_INS	0.010164282	2.33E-04	43.71528361
Jakun_Seq+Geno	Bateq	GIH	0.010239422	2.33E-04	44.03515502
Jakun_Seq+Geno	Cambodia	CheWong	0.006541184	1.66E-04	39.34704409
Jakun_Seq+Geno	Cambodia	CEU	0.008541504	1.86E-04	45.91059147
Jakun_Seq+Geno	Cambodia	GIH	0.008430767	1.71E-04	49.38061409
Jakun_Seq+Geno	Cambodia	SG_INS	0.008406935	1.70E-04	49.40569834
Jakun_Seq+Geno	Cambodia	Dusun	0.0086324	1.64E-04	52.47772371
Jakun_Seq+Geno	Cambodia	CHD	0.009756899	1.60E-04	60.82964598
Jakun_Seq+Geno	Cambodia	SG_CHS	0.009810787	1.59E-04	61.5780913
Jakun_Seq+Geno	Cambodia	CHB	0.009835477	1.59E-04	61.74451794
Jakun_Seq+Geno	CEU	Dusun	0.006572333	2.09E-04	31.4501377
Jakun_Seq+Geno	CEU	CheWong	0.007075266	2.22E-04	31.92364512
Jakun_Seq+Geno	CEU	SG_CHS	0.007763829	1.96E-04	39.604712
Jakun_Seq+Geno	CEU	CHD	0.00810175	1.98E-04	40.83214107
Jakun_Seq+Geno	CEU	CHB	0.008874817	2.04E-04	43.4462665
Jakun_Seq+Geno	CEU	SG_INS	0.027520625	2.84E-04	97.01797783
Jakun_Seq+Geno	CEU	GIH	0.02831268	2.88E-04	98.13957379
Jakun_Seq+Geno	CHB	CheWong	0.005957241	1.72E-04	34.71585669
Jakun_Seq+Geno	CHB	GIH	0.008764262	1.90E-04	46.1442671
Jakun_Seq+Geno	CHB	SG_INS	0.008709829	1.87E-04	46.57755481
Jakun_Seq+Geno	CHB	Dusun	0.010933729	1.80E-04	60.5883705
Jakun_Seq+Geno	CHB	CHD	0.014593249	1.87E-04	78.23337071
Jakun_Seq+Geno	CHB	SG_CHS	0.014506318	1.81E-04	80.23456726
Jakun_Seq+Geno	CHD	CheWong	0.006023583	1.74E-04	34.57245925
Jakun_Seq+Geno	CHD	GIH	0.008069413	1.86E-04	43.43974915

Jakun_Seq+Geno	CHD	SG_INS	0.008079324	1.84E-04	44.02333567
Jakun_Seq+Geno	CHD	Dusun	0.010937573	1.83E-04	59.69659376
Jakun_Seq+Geno	CHD	SG_CHS	0.014064356	1.84E-04	76.27368741
Jakun_Seq+Geno	CheWong	Dusun	0.005869317	1.90E-04	30.92547642
Jakun_Seq+Geno	CheWong	GIH	0.007149073	2.05E-04	34.89909694
Jakun_Seq+Geno	CheWong	SG_INS	0.007193469	2.02E-04	35.60112104
Jakun_Seq+Geno	Dusun	SG_INS	0.006480559	1.93E-04	33.57917035
Jakun_Seq+Geno	Dusun	GIH	0.006498889	1.90E-04	34.22635304
Jakun_Seq+Geno	GIH	SG_INS	0.026949865	2.58E-04	104.3947877
Jakun_Seq+Geno	JPT	CheWong	0.005962959	1.82E-04	32.7954061
Jakun_Seq+Geno	JPT	Bateq	0.006468938	1.92E-04	33.6330447
Jakun_Seq+Geno	JPT	YRI	0.008958786	2.36E-04	37.90457055
Jakun_Seq+Geno	JPT	CEU	0.009592959	2.08E-04	46.18812688
Jakun_Seq+Geno	JPT	SG_INS	0.00942146	1.92E-04	49.09564461
Jakun_Seq+Geno	JPT	GIH	0.009410046	1.90E-04	49.40727385
Jakun_Seq+Geno	JPT	Sonsogon	0.010676886	1.91E-04	56.02750508
Jakun_Seq+Geno	JPT	Lingkabau	0.010767608	1.89E-04	56.84950297
Jakun_Seq+Geno	JPT	Murut-P	0.010620231	1.86E-04	57.07559676
Jakun_Seq+Geno	JPT	Dusun	0.010810347	1.89E-04	57.1679423
Jakun_Seq+Geno	JPT	Rungus	0.01080302	1.80E-04	60.03835025
Jakun_Seq+Geno	JPT	Cambodia	0.00981095	1.62E-04	60.71030612
Jakun_Seq+Geno	JPT	SG_MAS	0.009992668	1.63E-04	61.14444095
Jakun_Seq+Geno	JPT	SG_CHS	0.014253997	1.88E-04	75.82632861
Jakun_Seq+Geno	JPT	CHD	0.014407603	1.90E-04	76.00326969
Jakun_Seq+Geno	JPT	CHB	0.015360081	1.89E-04	81.26539271
Jakun_Seq+Geno	Lingkabau	YRI	0.006017921	2.31E-04	25.99971122
Jakun_Seq+Geno	Lingkabau	Bateq	0.005274904	1.93E-04	27.33959065
Jakun_Seq+Geno	Lingkabau	CEU	0.00655432	2.05E-04	31.93595023
Jakun_Seq+Geno	Lingkabau	CheWong	0.005990498	1.85E-04	32.42278172
Jakun_Seq+Geno	Lingkabau	SG_INS	0.006498267	1.86E-04	34.9180583
Jakun_Seq+Geno	Lingkabau	GIH	0.006575296	1.88E-04	34.99846945
Jakun_Seq+Geno	Lingkabau	Cambodia	0.008706842	1.63E-04	53.37726929
Jakun_Seq+Geno	Lingkabau	SG_MAS	0.009855557	1.68E-04	58.56715632
Jakun_Seq+Geno	Lingkabau	CHD	0.010773867	1.83E-04	58.92769179
Jakun_Seq+Geno	Lingkabau	SG_CHS	0.01103959	1.85E-04	59.68345788
Jakun_Seq+Geno	Lingkabau	CHB	0.010871992	1.80E-04	60.31005505
Jakun_Seq+Geno	Lingkabau	Murut-P	0.013303499	2.01E-04	66.10165954
Jakun_Seq+Geno	Lingkabau	Dusun	0.015772631	2.17E-04	72.73310725
Jakun_Seq+Geno	Lingkabau	Rungus	0.01558654	2.09E-04	74.49168365
Jakun_Seq+Geno	Lingkabau	Sonsogon	0.017498172	2.18E-04	80.17002132
Jakun_Seq+Geno	Murut-P	YRI	0.006047252	2.33E-04	25.99272817

Jakun_Seq+Geno	Murut-P	Bateq	0.00532135	1.93E-04	27.60196922
Jakun_Seq+Geno	Murut-P	CEU	0.006476776	2.02E-04	32.07844216
Jakun_Seq+Geno	Murut-P	CheWong	0.00601986	1.83E-04	32.93163717
Jakun_Seq+Geno	Murut-P	GIH	0.006519469	1.90E-04	34.28787614
Jakun_Seq+Geno	Murut-P	SG_INS	0.006480865	1.86E-04	34.8486468
Jakun_Seq+Geno	Murut-P	Cambodia	0.008602932	1.61E-04	53.35717957
Jakun_Seq+Geno	Murut-P	CHB	0.010642601	1.79E-04	59.60904493
Jakun_Seq+Geno	Murut-P	CHD	0.010787311	1.80E-04	59.77651138
Jakun_Seq+Geno	Murut-P	SG_CHS	0.010817603	1.81E-04	59.91112789
Jakun_Seq+Geno	Murut-P	Dusun	0.013485797	1.99E-04	67.61220685
Jakun_Seq+Geno	Murut-P	Sonsogon	0.013385469	1.98E-04	67.70764226
Jakun_Seq+Geno	Murut-P	Rungus	0.013520276	1.94E-04	69.74155845
Jakun_Seq+Geno	Rungus	YRI	0.005957092	2.28E-04	26.12657237
Jakun_Seq+Geno	Rungus	Bateq	0.005245228	1.88E-04	27.8857777
Jakun_Seq+Geno	Rungus	CheWong	0.00590705	1.80E-04	32.8516
Jakun_Seq+Geno	Rungus	CEU	0.006525691	1.95E-04	33.49336647
Jakun_Seq+Geno	Rungus	SG_INS	0.006452491	1.80E-04	35.85139264
Jakun_Seq+Geno	Rungus	GIH	0.006554249	1.80E-04	36.39337473
Jakun_Seq+Geno	Rungus	Cambodia	0.008637662	1.60E-04	54.14321682
Jakun_Seq+Geno	Rungus	CHD	0.010852895	1.77E-04	61.20436756
Jakun_Seq+Geno	Rungus	CHB	0.010879933	1.71E-04	63.65704902
Jakun_Seq+Geno	Rungus	SG_CHS	0.011036981	1.73E-04	63.74298136
Jakun_Seq+Geno	Rungus	Sonsogon	0.01578039	2.11E-04	74.72198204
Jakun_Seq+Geno	Rungus	Dusun	0.016264954	2.11E-04	76.95789248
Jakun_Seq+Geno	SG_CHS	CheWong	0.005950785	1.72E-04	34.61160519
Jakun_Seq+Geno	SG_CHS	GIH	0.007782432	1.83E-04	42.55054627
Jakun_Seq+Geno	SG_CHS	SG_INS	0.00783565	1.80E-04	43.53504044
Jakun_Seq+Geno	SG_CHS	Dusun	0.011110992	1.83E-04	60.854389
Jakun_Seq+Geno	SG_MAS	Bateq	0.006000171	1.77E-04	33.97936798
Jakun_Seq+Geno	SG_MAS	CheWong	0.006393094	1.67E-04	38.28862115
Jakun_Seq+Geno	SG_MAS	YRI	0.008109804	2.05E-04	39.63497152
Jakun_Seq+Geno	SG_MAS	CEU	0.00874908	1.87E-04	46.84756365
Jakun_Seq+Geno	SG_MAS	GIH	0.008694189	1.74E-04	50.01536454
Jakun_Seq+Geno	SG_MAS	SG_INS	0.008687095	1.72E-04	50.44406727
Jakun_Seq+Geno	SG_MAS	Sonsogon	0.00981656	1.69E-04	58.07242114
Jakun_Seq+Geno	SG_MAS	Dusun	0.009816421	1.68E-04	58.26337242
Jakun_Seq+Geno	SG_MAS	Murut-P	0.009807373	1.66E-04	59.18282082
Jakun_Seq+Geno	SG_MAS	Cambodia	0.008780534	1.46E-04	60.15478787
Jakun_Seq+Geno	SG_MAS	CHD	0.009870226	1.61E-04	61.32095808
Jakun_Seq+Geno	SG_MAS	CHB	0.009984079	1.61E-04	61.99081939
Jakun_Seq+Geno	SG_MAS	Rungus	0.009889765	1.59E-04	62.30903851

Jakun_Seq+Geno	SG_MAS	SG_CHS	0.010002642	1.61E-04	62.31470275
Jakun_Seq+Geno	Sonsogon	YRI	0.0061004	2.38E-04	25.59664223
Jakun_Seq+Geno	Sonsogon	Bateq	0.005453624	1.97E-04	27.72680678
Jakun_Seq+Geno	Sonsogon	CEU	0.00652369	2.16E-04	30.18637418
Jakun_Seq+Geno	Sonsogon	CheWong	0.005835382	1.88E-04	31.04961217
Jakun_Seq+Geno	Sonsogon	GIH	0.006432758	1.98E-04	32.47925897
Jakun_Seq+Geno	Sonsogon	SG_INS	0.00647908	1.94E-04	33.32546438
Jakun_Seq+Geno	Sonsogon	Cambodia	0.008605523	1.62E-04	53.11405829
Jakun_Seq+Geno	Sonsogon	CHD	0.010801083	1.82E-04	59.42239495
Jakun_Seq+Geno	Sonsogon	SG_CHS	0.010991702	1.83E-04	60.05773064
Jakun_Seq+Geno	Sonsogon	CHB	0.010738637	1.79E-04	60.08307096
Jakun_Seq+Geno	Sonsogon	Dusun	0.016352491	2.20E-04	74.40661205
Jakun_Seq+Geno	YRI	Dusun	0.006032201	2.37E-04	25.47629545
Jakun_Seq+Geno	YRI	CheWong	0.007027755	2.42E-04	28.99523097
Jakun_Seq+Geno	YRI	SG_CHS	0.007357789	2.25E-04	32.7452521
Jakun_Seq+Geno	YRI	CHD	0.007443873	2.24E-04	33.17230007
Jakun_Seq+Geno	YRI	CHB	0.008179933	2.23E-04	36.6126365
Jakun_Seq+Geno	YRI	Bateq	0.01057187	2.81E-04	37.56874914
Jakun_Seq+Geno	YRI	Cambodia	0.008407408	2.06E-04	40.88063168
Jakun_Seq+Geno	YRI	GIH	0.023181576	2.99E-04	77.55376576
Jakun_Seq+Geno	YRI	CEU	0.02703934	3.48E-04	77.68138052
Jakun_Seq+Geno	YRI	SG_INS	0.023076662	2.93E-04	78.74379508
JPT	Bateq	Jakun_Seq+Geno	0.010812624	2.10E-04	51.59206372
JPT	Cambodia	Jakun_Seq+Geno	0.007470612	1.40E-04	53.27058831
JPT	CEU	Jakun_Seq+Geno	0.007688603	1.95E-04	39.38607548
JPT	CHB	Jakun_Seq+Geno	0.001921481	1.17E-04	16.44313176
JPT	CHD	Jakun_Seq+Geno	0.002873959	1.19E-04	24.07215446
JPT	CheWong	Jakun_Seq+Geno	0.011318603	2.00E-04	56.6979611
JPT	Dusun	Jakun_Seq+Geno	0.006471215	1.51E-04	42.82895618
JPT	GIH	Jakun_Seq+Geno	0.007871516	1.81E-04	43.53836972
JPT	Lingkabau	Jakun_Seq+Geno	0.006513954	1.52E-04	42.75998582
JPT	Murut-P	Jakun_Seq+Geno	0.006661331	1.50E-04	44.41857451
JPT	Rungus	Jakun_Seq+Geno	0.006478542	1.46E-04	44.32952989
JPT	SG_CHS	Jakun_Seq+Geno	0.003027564	1.20E-04	25.13818674
JPT	SG_INS	Jakun_Seq+Geno	0.007860102	1.76E-04	44.69573583
JPT	SG_MAS	Jakun_Seq+Geno	0.007288894	1.40E-04	52.15030786
JPT	Sonsogon	Jakun_Seq+Geno	0.006604676	1.59E-04	41.5744416
JPT	YRI	Jakun_Seq+Geno	0.008322776	2.22E-04	37.48206984
Lingkabau	Bateq	Jakun_Seq+Geno	0.014903817	2.43E-04	61.4085405
Lingkabau	Cambodia	Jakun_Seq+Geno	0.011471878	1.86E-04	61.59891051
Lingkabau	CEU	Jakun_Seq+Geno	0.013624401	2.34E-04	58.11900274

Lingkabau	CHB	Jakun_Seq+Geno	0.009306728	1.82E-04	51.15182687
Lingkabau	CHD	Jakun_Seq+Geno	0.009404853	1.83E-04	51.4797797
Lingkabau	CheWong	Jakun_Seq+Geno	0.014188223	2.33E-04	61.02013888
Lingkabau	Dusun	Jakun_Seq+Geno	0.00440609	1.69E-04	26.01346456
Lingkabau	GIH	Jakun_Seq+Geno	0.013603424	2.24E-04	60.6356904
Lingkabau	Jakun_Seq+Geno	JPT	0.009411113	1.82E-04	51.69277934
Lingkabau	Murut-P	Jakun_Seq+Geno	0.006875221	1.82E-04	37.702451
Lingkabau	Rungus	Jakun_Seq+Geno	0.00459218	1.66E-04	27.62521271
Lingkabau	SG_CHS	Jakun_Seq+Geno	0.009139131	1.76E-04	51.83263302
Lingkabau	SG_INS	Jakun_Seq+Geno	0.013680453	2.23E-04	61.32381982
Lingkabau	SG_MAS	Jakun_Seq+Geno	0.010323164	1.80E-04	57.31217363
Lingkabau	Sonsogon	Jakun_Seq+Geno	0.002680549	1.71E-04	15.70144595
Lingkabau	YRI	Jakun_Seq+Geno	0.014160799	2.60E-04	54.42416172
Murut-P	Bateq	Jakun_Seq+Geno	0.013916758	2.34E-04	59.56355066
Murut-P	Cambodia	Jakun_Seq+Geno	0.010635175	1.79E-04	59.49175582
Murut-P	CEU	Jakun_Seq+Geno	0.012761332	2.30E-04	55.53921782
Murut-P	CHB	Jakun_Seq+Geno	0.008595507	1.77E-04	48.66290308
Murut-P	CHD	Jakun_Seq+Geno	0.008450797	1.75E-04	48.42439814
Murut-P	CheWong	Jakun_Seq+Geno	0.013218248	2.22E-04	59.42148206
Murut-P	Dusun	Jakun_Seq+Geno	0.005752311	1.66E-04	34.74930831
Murut-P	GIH	Jakun_Seq+Geno	0.012718639	2.21E-04	57.66512457
Murut-P	Jakun_Seq+Geno	Lingkabau	0.005934608	1.65E-04	36.06339529
Murut-P	Jakun_Seq+Geno	JPT	0.008617877	1.79E-04	48.02148485
Murut-P	Jakun_Seq+Geno	SG_MAS	0.009430735	1.75E-04	53.83939421
Murut-P	Rungus	Jakun_Seq+Geno	0.005717832	1.59E-04	36.05389093
Murut-P	SG_CHS	Jakun_Seq+Geno	0.008420505	1.75E-04	48.16021353
Murut-P	SG_INS	Jakun_Seq+Geno	0.012757242	2.20E-04	58.00501119
Murut-P	Sonsogon	Jakun_Seq+Geno	0.005852638	1.72E-04	34.03232402
Murut-P	YRI	Jakun_Seq+Geno	0.013190856	2.56E-04	51.44426746
Rungus	Bateq	Jakun_Seq+Geno	0.011955702	2.31E-04	51.79464428
Rungus	Cambodia	Jakun_Seq+Geno	0.008563267	1.67E-04	51.14080247
Rungus	CEU	Jakun_Seq+Geno	0.010675239	2.24E-04	47.62048329
Rungus	CHB	Jakun_Seq+Geno	0.006320996	1.66E-04	38.04628231
Rungus	CHD	Jakun_Seq+Geno	0.006348035	1.65E-04	38.54911444
Rungus	CheWong	Jakun_Seq+Geno	0.011293879	2.15E-04	52.56615899
Rungus	Dusun	Jakun_Seq+Geno	9.36E-04	1.37E-04	6.85237283
Rungus	GIH	Jakun_Seq+Geno	0.01064668	2.13E-04	49.9992701
Rungus	Jakun_Seq+Geno	Lingkabau	0.001614389	1.37E-04	11.78528737
Rungus	Jakun_Seq+Geno	Murut-P	0.003680653	1.51E-04	24.36212445
Rungus	Jakun_Seq+Geno	JPT	0.006397909	1.68E-04	38.04167796
Rungus	Jakun_Seq+Geno	SG_MAS	0.007311165	1.64E-04	44.70416719

Rungus	SG_CHS	Jakun_Seq+Geno	0.006163949	1.60E-04	38.4542536
Rungus	SG_INS	Jakun_Seq+Geno	0.010748438	2.09E-04	51.36981016
Rungus	Sonsogon	Jakun_Seq+Geno	0.001420539	1.44E-04	9.859079998
Rungus	YRI	Jakun_Seq+Geno	0.011243838	2.56E-04	43.98239304
SG_CHS	CheWong	Jakun_Seq+Geno	0.008177866	1.81E-04	45.26562087
SG_CHS	Dusun	Jakun_Seq+Geno	0.003017659	1.37E-04	22.00904028
SG_CHS	GIH	Jakun_Seq+Geno	0.006346219	1.72E-04	36.96484379
SG_CHS	Jakun_Seq+Geno	CHB	-3.78E-04	1.03E-04	3.666406396
SG_CHS	Jakun_Seq+Geno	JPT	-1.25E-04	1.07E-04	-1.166575722
SG_CHS	Jakun_Seq+Geno	CHD	6.43E-05	1.01E-04	0.635723878
SG_CHS	Jakun_Seq+Geno	Sonsogon	0.003136949	1.43E-04	21.99439173
SG_CHS	Jakun_Seq+Geno	Lingkabau	0.003089061	1.36E-04	22.71264355
SG_CHS	Jakun_Seq+Geno	Rungus	0.00309167	1.31E-04	23.6058754
SG_CHS	Jakun_Seq+Geno	Murut-P	0.003311048	1.39E-04	23.83086923
SG_CHS	Jakun_Seq+Geno	YRI	0.006770863	2.20E-04	30.74000405
SG_CHS	Jakun_Seq+Geno	CEU	0.006364823	1.91E-04	33.36450337
SG_CHS	Jakun_Seq+Geno	SG_MAS	0.004126009	1.19E-04	34.60807048
SG_CHS	Jakun_Seq+Geno	Cambodia	0.004317865	1.24E-04	34.88476849
SG_CHS	Jakun_Seq+Geno	Bateq	0.008377521	1.93E-04	43.40225393
SG_CHS	SG_INS	Jakun_Seq+Geno	0.006293001	1.72E-04	36.50667301
SG_INS	Jakun_Seq+Geno	Bateq	0.016617287	2.47E-04	67.15717643
SG_INS	Jakun_Seq+Geno	Cambodia	0.018374634	2.19E-04	83.89520989
SG_INS	Jakun_Seq+Geno	CEU	-7.39E-04	1.56E-04	4.73439735
SG_INS	Jakun_Seq+Geno	CHB	0.018071741	2.27E-04	79.61801318
SG_INS	Jakun_Seq+Geno	CHD	0.018702246	2.31E-04	81.11459427
SG_INS	Jakun_Seq+Geno	CheWong	0.0195881	2.51E-04	78.09213509
SG_INS	Jakun_Seq+Geno	Dusun	0.020301011	2.43E-04	83.60834019
SG_INS	Jakun_Seq+Geno	GIH	-1.68E-04	1.19E-04	-1.416889297
SG_INS	Jakun_Seq+Geno	JPT	0.01736011	2.25E-04	77.22339219
SG_INS	Jakun_Seq+Geno	Lingkabau	0.020283302	2.45E-04	82.79958842
SG_INS	Jakun_Seq+Geno	Murut-P	0.020300704	2.35E-04	86.50954519
SG_INS	Jakun_Seq+Geno	Rungus	0.020329078	2.37E-04	85.63785839
SG_INS	Jakun_Seq+Geno	SG_CHS	0.01894592	2.28E-04	82.95692391
SG_INS	Jakun_Seq+Geno	SG_MAS	0.018094475	2.18E-04	83.12250866
SG_INS	Jakun_Seq+Geno	Sonsogon	0.02030249	2.47E-04	82.20973349
SG_INS	Jakun_Seq+Geno	YRI	0.003704907	2.22E-04	16.67421441
SG_MAS	Bateq	Jakun_Seq+Geno	0.003210134	1.62E-04	19.76375735
SG_MAS	Cambodia	Jakun_Seq+Geno	4.30E-04	9.96E-05	4.3133481
SG_MAS	CEU	Jakun_Seq+Geno	4.61E-04	1.61E-04	2.869549278
SG_MAS	CHB	Jakun_Seq+Geno	-7.74E-04	1.04E-04	7.476007716
SG_MAS	CHD	Jakun_Seq+Geno	-6.60E-04	1.02E-04	6.449118329

SG_MAS	CheWong	Jakun_Seq+Geno	0.00281721	1.49E-04	18.96880145
SG_MAS	Dusun	Jakun_Seq+Geno	-6.06E-04	1.17E-04	5.180886076
SG_MAS	GIH	Jakun_Seq+Geno	5.16E-04	1.45E-04	3.569949612
SG_MAS	Jakun_Seq+Geno	JPT	-7.82E-04	1.09E-04	7.180410021
SG_MAS	Jakun_Seq+Geno	Lingkabau	-6.45E-04	1.14E-04	5.668622975
SG_MAS	Murut-P	Jakun_Seq+Geno	-5.97E-04	1.13E-04	5.271713929
SG_MAS	Rungus	Jakun_Seq+Geno	-6.79E-04	1.11E-04	6.112852583
SG_MAS	SG_CHS	Jakun_Seq+Geno	-7.92E-04	1.02E-04	7.733999543
SG_MAS	SG_INS	Jakun_Seq+Geno	5.23E-04	1.44E-04	3.643232367
SG_MAS	Sonsogon	Jakun_Seq+Geno	-6.06E-04	1.20E-04	5.031998469
SG_MAS	YRI	Jakun_Seq+Geno	0.001100501	1.89E-04	5.811403919
Sonsogon	Bateq	Jakun_Seq+Geno	0.017972217	2.64E-04	68.06038274
Sonsogon	Cambodia	Jakun_Seq+Geno	0.014820318	2.18E-04	68.05831251
Sonsogon	CEU	Jakun_Seq+Geno	0.016902151	2.60E-04	64.99594815
Sonsogon	CHB	Jakun_Seq+Geno	0.012687204	2.12E-04	59.82513756
Sonsogon	CHD	Jakun_Seq+Geno	0.012624758	2.10E-04	60.17477466
Sonsogon	CheWong	Jakun_Seq+Geno	0.017590459	2.58E-04	68.30555445
Sonsogon	Dusun	Jakun_Seq+Geno	0.00707335	1.98E-04	35.65714442
Sonsogon	GIH	Jakun_Seq+Geno	0.016993083	2.48E-04	68.42235728
Sonsogon	Jakun_Seq+Geno	Lingkabau	0.005927669	1.84E-04	32.22790356
Sonsogon	Jakun_Seq+Geno	Rungus	0.007645451	1.94E-04	39.37689994
Sonsogon	Jakun_Seq+Geno	Murut-P	0.010040372	2.03E-04	49.44792244
Sonsogon	Jakun_Seq+Geno	JPT	0.012748955	2.14E-04	59.53228419
Sonsogon	Jakun_Seq+Geno	SG_MAS	0.013609281	2.12E-04	64.08738867
Sonsogon	SG_CHS	Jakun_Seq+Geno	0.012434139	2.09E-04	59.59573132
Sonsogon	SG_INS	Jakun_Seq+Geno	0.016946761	2.47E-04	68.60409152
Sonsogon	YRI	Jakun_Seq+Geno	0.017325441	2.75E-04	62.90181777
YRI	Bateq	Jakun_Seq+Geno	0.062488583	5.01E-04	124.701781
YRI	Cambodia	Jakun_Seq+Geno	0.064653044	4.87E-04	132.6378551
YRI	CEU	Jakun_Seq+Geno	0.046021113	4.29E-04	107.1920689
YRI	CHB	Jakun_Seq+Geno	0.06488052	5.03E-04	129.0947048
YRI	CHD	Jakun_Seq+Geno	0.065616579	4.99E-04	131.3741668
YRI	CheWong	Jakun_Seq+Geno	0.066032698	5.14E-04	128.3722861
YRI	Dusun	Jakun_Seq+Geno	0.067028251	5.14E-04	130.5192346
YRI	GIH	Jakun_Seq+Geno	0.049878876	4.24E-04	117.6596595
YRI	Jakun_Seq+Geno	JPT	0.064101667	4.98E-04	128.7071728
YRI	Jakun_Seq+Geno	Sonsogon	0.066960052	5.20E-04	128.8230839
YRI	Jakun_Seq+Geno	Lingkabau	0.067042532	5.15E-04	130.2315483
YRI	Jakun_Seq+Geno	Murut-P	0.067013201	5.11E-04	131.2368186
YRI	Jakun_Seq+Geno	Rungus	0.067103361	5.11E-04	131.3392457
YRI	Jakun_Seq+Geno	SG_MAS	0.064950649	4.87E-04	133.3156625

YRI	SG_CHS	Jakun_Seq+Geno	0.065702664	5.06E-04	129.801015
YRI	SG_INS	Jakun_Seq+Geno	0.049983791	4.25E-04	117.4827445

Note:
A negative F3 value is an indication of admixture in the (A) population from the two source populations [(B) and (C)].
The more negative the F3 value, the stronger the evidence for admixture.
A highly negative Z-score (typically less than -2) is taken as evidence against the null hypothesis and suggests that the (A) population is admixed from the two sou
