# Supplementary material for: High-coverage whole-genome sequencing of a Jakun individual from the “Orang Asli” Proto-Malay subtribe from Peninsular Malaysia
Source: Hum Genome Var. 2025 Jan 8;12:4. doi: 10.1038/s41439-024-00308-6 (PMC11707147; doi:10.1038/s41439-024-00308-6)
Supplement: Supplementary file 10 — Table S4 [file 41439_2024_308_MOESM10_ESM.pdf]

Table S4 Potentially damaging and deleterious variants as predicted by computational tools SIFT, Polyphen-2 and CADD (Phred score ≥ 15)

Predicted tool	Class predicted	Number of unique variants	Number of genes related to unique variants
SIFT	Damaging	1683	1362
Polyphen-2	Possibly & Probably Damaging	1313	1086
CADD	Phred Score ≥ 15	3727	2818
Common between SIFT, Polyphen-2 & CADD	-	825	720
