# Supplementary material for: High-coverage whole-genome sequencing of a Jakun individual from the “Orang Asli” Proto-Malay subtribe from Peninsular Malaysia
Source: Hum Genome Var. 2025 Jan 8;12:4. doi: 10.1038/s41439-024-00308-6 (PMC11707147; doi:10.1038/s41439-024-00308-6)
Supplement: Supplementary file 11 — Table S5 [file 41439_2024_308_MOESM11_ESM.pdf]

CHR_ID	CHR_POS	MAPPED_GENE	DOWNSTREAM_GENE_DISTANCE	DISEASE/TRAIT	INITIAL SAMPLE SIZE	STRONGEST SNP-RISK ALLELE	Jakun Risk Allele	RISK ALLELE FREQUENCY	P-VALUE	95% CI (TEXT)	CNV	MAPPED_TRAIT	STUDY ACCESSION
1	1.59E+08	OR10Z1		Reticulocyte production index	39,566 European ancestry individuals	rs857685-C	A > C	0.2545	2.00E-15	[0.048-0.079] SD units decrease	N	erythrocyte measurement	GCST90281221
1	1.8E+08	TDRD5		Age-related cognitive decline (memory) (slope of z-scores)	1,145 European ancestry individuals	rs61310274-C	G > C	0.34	2.00E-06	unit decrease	N	cognitive decline measurement	GCST009441
1	2.03E+08	CHIT1		Chitotriosidase-1 level in Chronic kidney disease with hypertension and no diabetes (10460_1)	466 African American individuals	rs2297950-T	C > T	0.26	1.00E-24	[0.56-0.84] unit increase	N	chitotriosidase-1 measurement	GCST90232881
1	24964519	RUNX3		Eczema	20,016 European ancestry cases, 380,433 European ancestry controls	rs6672420-T	A > T	0.4793	5.00E-12		N	Eczematoid dermatitis	GCST90044763
1	24964519	RUNX3		Psoriasis vulgaris	5,072 European ancestry cases, 478,102 European ancestry controls, 206 East Asian ancestry cases, 172,289 East Asian ancestry controls	rs6672420-T	A > T	NR	7.00E-10	[0.084-0.163] unit increase	N	psoriasis vulgaris	GCST90018907
1	24964519	RUNX3		Psoriasis or type 2 diabetes (trans-disease meta-analysis)(opposite effect)	11,024 European ancestry psoriasis cases, 74,124 European ancestry diabetes cases, 840,342 European ancestry controls	rs6672420-T	A > T	NR	3.00E-12		N	psoriasis, type 2 diabetes mellitus	GCST011990
1	53247055	LRP8		General risk tolerance (MTAG)	975,353 European ancestry individuals	rs5174-T	C > T	0.405	1.00E-08	[0.0045-0.0093] unit decrease	N	risk-taking behaviour	GCST007325
1	53247055	LRP8		Cortical surface area	35,657 White British ancestry individuals	rs5174-T	C > T	0.4164	2.00E-12		N	cortical surface area	GCST90091060
1	53247055	LRP8		Cortical thickness	35,657 White British ancestry individuals	rs5174-T	C > T	0.4164	1.00E-17		N	cortical thickness	GCST90091061
1	53247055	LRP8		Vertex-wise sulcal depth	33,748 European ancestry individuals	rs5174-T	C > T	0.4	3.00E-26	z score increase	N	brain measurement	GCST90095129
1	53247055	LRP8		Vertex-wise cortical surface area	33,748 European ancestry individuals	rs5174-T	C > T	0.4	4.00E-12	z score increase	N	cortical surface area	GCST90095130
1	53247055	LRP8		Vertex-wise cortical thickness	33,748 European ancestry individuals	rs5174-T	C > T	0.4	1.00E-28	z score increase	N	cortical thickness	GCST90095131
1	55043912	PCSK9		LDL cholesterol levels	12,685 Chinese ancestry individuals	rs151193009-T	C > T	0.013	8.00E-32	[0.54-0.74] unit decrease	N	LDL cholesterol change	GCST009917
1	55043912	PCSK9		Total cholesterol levels	12,685 Chinese ancestry individuals	rs151193009-T	C > T	0.013	5.00E-25	[0.46-0.66] unit decrease	N	total cholesterol measurement	GCST009919
1	55043912	PCSK9		Low density lipoprotein cholesterol levels	6,949 Korean ancestry individuals	rs151193009-T	C > T	0.013	6.00E-17	[0.14-0.23] unit decrease	N	low density lipoprotein cholesterol measurement	GCST007442
1	55043912	PCSK9		Total cholesterol levels	6,949 Korean ancestry individuals	rs151193009-T	C > T	0.013	8.00E-14	[14.33-24.48] unit decrease	N	total cholesterol measurement	GCST007441
1	55043912	PCSK9		Total cholesterol levels	135,808 East Asian ancestry individuals	rs151193009-T	C > T	0.009536	8.00E-90	[0.33-0.41] unit decrease	N	total cholesterol measurement	GCST90018754
1	55043912	PCSK9		Medication use (antithrombotic agents)	54,220 East Asian ancestry cases, 124,506 East Asian ancestry controls	rs151193009-T	C > T	0.0098259	2.00E-08	[0.15-0.31] unit decrease	N	Antithrombotic agent use measurement	GCST90018762

1	55043912	PCSK9		Medication use (salicylic acid and derivatives)	41,461 East Asian ancestry cases, 137,265 East Asian ancestry controls	rs151193009-T	C > T	0.0098259	7.00E-11	[0.2-0.37] unit decrease	N	aspirin use measurement	GCST90018775
1	55043912	PCSK9		Medication use (vasodilators used in cardiac diseases)	17,050 East Asian ancestry cases, 161,676 East Asian ancestry controls	rs151193009-T	C > T	0.0098259	4.00E-09	[0.25-0.5] unit decrease	N	Vasodilators used in cardiac diseases use	GCST90018763
1	55043912	PCSK9		Myocardial infarction	14,992 East Asian ancestry cases, 146,214 East Asian ancestry controls	rs151193009-T	C > T	0.01038088	9.00E-10	[0.3-0.57] unit decrease	N	myocardial infarction	GCST90018657
1	55043912	PCSK9		Total cholesterol levels	9,947 Han Chinese ancestry individuals, 294	rs151193009-T	C > T	NR	5.00E-15	[0.38-0.62] unit decrease	N	total cholesterol measurement	GCST90319529
1	55043912	PCSK9		LDL cholesterol levels	9,947 Han Chinese ancestry individuals, 294 individuals	rs151193009-T	C > T	NR	9.00E-18	[0.43-0.67] unit decrease	N	low density lipoprotein cholesterol measurement	GCST90319533
1	55043912	PCSK9		Low density lipoprotein cholesterol levels	146,492 East Asian ancestry individuals	rs151193009-T	C > T	0.00888823	2.00E-44	[0.42-0.55] unit decrease	N	low density lipoprotein cholesterol measurement	GCST90239657
1	55043912	PCSK9		Medication use (salicylic acid and derivatives)	61,583 European ancestry cases, 50,427 European ancestry controls, 41,461 East Asian ancestry cases, 137,265 East Asian ancestry controls	rs151193009-T	C > T	NR	7.00E-11	[0.2-0.37] unit decrease	N	aspirin use measurement	GCST90018995
1	55043912	PCSK9		Medication use (vasodilators used in cardiac diseases)	5,546 European ancestry cases, 237,113 European ancestry controls, 17,050 East Asian ancestry cases, 161,676 East Asian ancestry controls	rs151193009-T	C > T	NR	4.00E-09	[0.25-0.5] unit decrease	N	Vasodilators used in cardiac diseases use measurement	GCST90018983
1	55043912	PCSK9		Medication use (antithrombotic agents)	67,653 European ancestry cases, 85,986 European ancestry controls, 54,220 East Asian ancestry cases, 124,506 East Asian ancestry controls	rs151193009-T	C > T	NR	2.00E-08	[0.15-0.31] unit decrease	N	Antithrombotic agent use measurement	GCST90018982
1	55043912	PCSK9		Non-HDL cholesterol levels	146,492 East Asian ancestry individuals	rs151193009-T	C > T	0.00904818	7.00E-39	[0.4-0.53] unit decrease	N	non-high density lipoprotein cholesterol measurement	GCST90239669
1	55043912	PCSK9		Total cholesterol levels	58,701 Korean ancestry individuals	rs151193009-T	C > T	NR	2.00E-67	[12.86-16.14] unit decrease	N	total cholesterol measurement	GCST90134518
1	55043912	PCSK9		LDL cholesterol levels	288,127 East Asian ancestry individuals	rs151193009-T	C > T	0.0153	##########	[0.5-0.57] unit decrease	N	low density lipoprotein cholesterol measurement	GCST90244006
1	55043912	PCSK9		Total cholesterol levels	288,127 East Asian ancestry individuals	rs151193009-T	C > T	0.0153	##########	[0.44-0.5] unit decrease	N	total cholesterol measurement	GCST90244008
1	55043912	PCSK9		LDL cholesterol levels	58,701 Korean ancestry individuals	rs151193009-T	C > T	NR	2.00E-81	[13.01-15.97] unit decrease	N	low density lipoprotein cholesterol measurement	GCST90134520
1	64177594	ROR1		Cortical surface area	35,657 White British ancestry individuals	rs7527017-T	C > T	0.3236	7.00E-14		N	cortical surface area	GCST90091060
1	64177594	ROR1		Cortical thickness	35,657 White British ancestry individuals	rs7527017-T	C > T	0.3236	3.00E-10		N	cortical thickness	GCST90091061

1	64177594	ROR1		Vertex-wise cortical surface area	33,748 European ancestry individuals	rs7527017-T	C > T	0.35	8.00E-15	z score increase	N	cortical surface area	GCST90095130
1	64177594	ROR1		Vertex-wise cortical thickness	33,748 European ancestry individuals	rs7527017-T	C > T	0.35	3.00E-10	z score increase	N	cortical thickness	GCST90095131
1	64177594	ROR1		Height	455,180 Hispanic or Latin American individuals	rs7527017-T	C > T	0.3119	9.00E-12	[0.011-0.02] unit decrease	N	body height	GCST90245844
1	78891675	ADGRL4		Lymphocyte count	524,923 European ancestry individuals	rs2275902-C	G > C	0.297647	5.00E-15	[0.012-0.02] SD unit decrease	N	lymphocyte count	GCST90002316
1	78891675	ADGRL4		Lymphocyte count	643,370 African American or Afro-Caribbean, African ancestry, European ancestry, East Asian ancestry, Hispanic or Latin American and South Asian ancestry individuals	rs2275902-C	G > C	0.28737	4.00E-13		N	lymphocyte count	GCST90002320
1	78891675	ADGRL4		Diastolic blood pressure	1,028,980 European ancestry individuals	rs2275902-C	G > C	NR	3.00E-08	[0.058-0.122] unit decrease	N	diastolic blood pressure	GCST90310295
1	8957145	CA6		Serum levels of protein CA6	5,367 Icelandic ancestry individuals	rs2274333-G	A > G	0.3378	##########	[0.37-0.45] unit increase	N	blood protein measurement	GCST90088335
1	8957145	CA6		Median relapse-independent longitudinal age-related multiple sclerosis severity score in relapse-onset multiple sclerosis	1,813 European ancestry individuals	rs2274333-G	A > G	0.29	5.00E-06	unit decrease	N	multiple sclerosis symptom measurement	GCST90274850
1	99896369			Plasma metabolome feature (hilic_7309)	7 Asian ancestry children, 36 Black or African American children, 352 European ancestry children, 1 Native Hawaiian or other Pacific Islander child, 45 children	chr1:99896369-A	G > A	NR	2.00E-08	[0.46-0.95] unit decrease	N	blood metabolite measurement	GCST90280763
2	21008652	APOB		Fasting triglycerides levels in very small VLDL	4,734 individuals	rs676210-A	G > A	0.21369104	5.00E-08	[0.087-0.185] mmol/l decrease	N	triglyceride measurement, very low density lipoprotein cholesterol measurement	GCST90091641
2	21008652	APOB		HDL cholesterol	215,551 European ancestry individuals, 57,332 African American individuals, 24,743 Hispanic individuals	rs676210-A	G > A	0.2052	5.00E-63	[0.048-0.061] mg dl-1 increase	N	high density lipoprotein cholesterol measurement	GCST006611
2	21008652	APOB		Triglycerides to total lipids ratio in small VLDL	4,435 East Asian ancestry individuals, 11,340 South Asian ancestry individuals, 120,241 European ancestry individuals	rs676210-A	G > A	0.2713	5.00E-49	[0.06-0.078] unit decrease	N	triglycerides:total lipids ratio, very low density lipoprotein cholesterol measurement	GCST90302115
2	21008652	APOB		Estimated degree of unsaturation	4,435 East Asian ancestry individuals, 11,340 South Asian ancestry individuals, 120,241 European ancestry individuals	rs676210-A	G > A	0.2716	6.00E-15	[0.028-0.046] unit increase	N	fatty acid measurement	GCST90302121
2	21008652	APOB		Mean diameter of HDL particles	4,435 East Asian ancestry individuals, 11,340 South Asian ancestry individuals, 120,241 European ancestry individuals	rs676210-A	G > A	0.2697	2.00E-33	[0.047-0.065] unit increase	N	high density lipoprotein particle size measurement	GCST90301972

2	21008652	APOB		Total cholesterol in large HDL	4,435 East Asian ancestry individuals, 11,340 South Asian ancestry individuals, 120,241 European ancestry individuals	rs676210-A	**G > A**	0.2698	4.00E-44	[0.055-0.073] unit increase	N	total cholesterol change measurement, high density lipoprotein cholesterol measurement	GCST90301995
2	21008652	APOB		Cholesterol esters in large HDL	4,435 East Asian ancestry individuals, 11,340 South Asian ancestry individuals, 120,241 European ancestry individuals	rs676210-A	**G > A**	0.2698	8.00E-44	[0.055-0.073] unit increase	N	cholesterol esters in large HDL measurement	GCST90301997
2	21008652	APOB		Free cholesterol in large HDL	4,435 East Asian ancestry individuals, 11,340 South Asian ancestry individuals, 120,241 European ancestry individuals	rs676210-A	**G > A**	0.2698	3.00E-43	[0.055-0.073] unit increase	N	free cholesterol in large HDL measurement	GCST90301999
2	21008652	APOB		Free cholesterol to total lipids ratio in large HDL	4,435 East Asian ancestry individuals, 11,340 South Asian ancestry individuals, 120,241 European ancestry individuals	rs676210-A	**G > A**	0.2674	3.00E-25	[0.039-0.057] unit increase	N	free cholesterol:total lipids ratio, high density lipoprotein cholesterol	GCST90302000
2	21008652	APOB		Total lipids in large HDL	4,435 East Asian ancestry individuals, 11,340 South Asian ancestry individuals, 120,241 European ancestry individuals	rs676210-A	**G > A**	0.2698	1.00E-39	[0.052-0.07] unit increase	N	total lipids in large HDL	GCST90302001
2	21008652	APOB		Concentration of large HDL particles	4,435 East Asian ancestry individuals, 11,340 South Asian ancestry individuals, 120,241 European ancestry individuals	rs676210-A	**G > A**	0.2698	1.00E-38	[0.051-0.069] unit increase	N	high density lipoprotein cholesterol measurement	GCST90302002
2	21008652	APOB		Phospholipids in large HDL	4,435 East Asian ancestry individuals, 11,340 South Asian ancestry individuals, 120,241 European ancestry individuals	rs676210-A	**G > A**	0.2699	8.00E-39	[0.051-0.069] unit increase	N	phospholipids in large HDL measurement	GCST90302003
2	21008652	APOB		Ceramide_42:2_[M-H]1- levels	13,814 British ancestry individuals	rs676210-A	**G > A**	NR	3.00E-12	[0.061-0.109] unit decrease	N	phosphatidylethanolamine	GCST90060212
2	21008652	APOB		High density lipoprotein cholesterol levels	48,057 Hispanic individuals	rs676210-A	**G > A**	0.245812	3.00E-14	[0.044-0.074] unit increase	N	high density lipoprotein cholesterol measurement	GCST90239653
2	21008652	APOB		High density lipoprotein cholesterol levels	1,320,016 European ancestry individuals	rs676210-A	**G > A**	0.214148	##########	[0.057-0.064] unit increase	N	high density lipoprotein cholesterol measurement	GCST90239652
2	21008652	APOB		High density lipoprotein cholesterol levels	99,432 Admixed African or African ancestry individuals	rs676210-A	**G > A**	0.151084	6.00E-16	[0.04-0.065] unit increase	N	high density lipoprotein cholesterol measurement	GCST90239650

2	21008652	APOB		Total cholesterol to total lipids ratio in medium HDL	4,435 East Asian ancestry individuals, 11,340 South Asian ancestry individuals, 120,241 European ancestry individuals	rs676210-A	**G > A**	0.2681	2.00E-33	[0.046-0.064] unit increase	N	esterified cholesterol measurement, high density lipoprotein	GCST90302032
2	21008652	APOB		Phospholipids to total lipids ratio in very large HDL	4,435 East Asian ancestry individuals, 11,340 South Asian ancestry individuals, 120,241 European ancestry individuals	rs676210-A	**G > A**	0.268	1.00E-12	[0.024-0.042] unit increase	N	phospholipids: total lipids ratio, high density lipoprotein	GCST90302135
2	21008652	APOB		Triglyceride levels	1,320,016 European ancestry individuals	rs676210-A	**G > A**	0.214007	##########	[0.067-0.074] unit decrease	N	triglyceride measurement	GCST90239664
2	21008652	APOB		HDL cholesterol levels	22,000 Pakistani and Bangladeshi ancestry individuals	rs676210-A	**G > A**	0.54	8.00E-06	[0.03-0.07] unit increase	N	high density lipoprotein cholesterol measurement	GCST90140946
2	21008652	APOB		Triglyceride levels	48,057 Hispanic individuals	rs676210-A	**G > A**	0.245749	4.00E-13	[0.04-0.069] unit decrease	N	triglyceride measurement	GCST90239665
2	21008652	APOB		Total lipids in VLDL (UKB data field 23424)	88,329 European ancestry individuals	rs676210-A	**G > A**	0.205238	2.00E-91	[0.1-0.13] unit decrease	N	total lipids in VLDL	GCST90269521
2	21008652	APOB		Total lipids in HDL (UKB data field 23426)	88,329 European ancestry individuals	rs676210-A	**G > A**	0.205238	7.00E-33	[0.053-0.073] unit increase	N	total lipids in HDL	GCST90269523
2	21008652	APOB		Total lipids in chylomicrons and extremely large VLDL (UKB data field 23482)	88,329 European ancestry individuals	rs676210-A	**G > A**	0.205238	2.00E-55	[0.078-0.1] unit decrease	N	total lipids in chylomicrons and extremely large VLDL	GCST90269579
2	21008652	APOB		Concentration of chylomicrons and extremely large VLDL particles (UKB data field 23481)	88,329 European ancestry individuals	rs676210-A	**G > A**	0.205238	2.00E-61	[0.082-0.104] unit decrease	N	concentration of chylomicrons and extremely large VLDL	GCST90269578
2	21008652	APOB		Concentration of large VLDL particles (UKB data field 23495)	88,329 European ancestry individuals	rs676210-A	**G > A**	0.205238	3.00E-90	[0.1-0.13] unit decrease	N	very low density lipoprotein cholesterol measurement	GCST90269592
2	21008652	APOB		Total lipids in large VLDL (UKB data field 23496)	88,329 European ancestry individuals	rs676210-A	**G > A**	0.205238	3.00E-89	[0.1-0.12] unit decrease	N	total lipids in large VLDL	GCST90269593
2	21008652	APOB		Concentration of medium HDL particles (UKB data field 23565)	88,329 European ancestry individuals	rs676210-A	**G > A**	0.205238	3.00E-33	[0.054-0.075] unit increase	N	high density lipoprotein cholesterol measurement	GCST90269662
2	21008652	APOB		Phospholipids in medium HDL (UKB data field 23567)	88,329 European ancestry individuals	rs676210-A	**G > A**	0.205238	2.00E-27	[0.049-0.07] unit increase	N	phospholipids in medium HDL measurement	GCST90269664
2	21008652	APOB		Cholesterol in medium HDL (UKB data field 23568)	88,329 European ancestry individuals	rs676210-A	**G > A**	0.205238	7.00E-48	[0.068-0.089] unit increase	N	cholesterol in medium HDL measurement	GCST90269665
2	21008652	APOB		Total lipids in medium HDL (UKB data field 23566)	88,329 European ancestry individuals	rs676210-A	**G > A**	0.205238	4.00E-32	[0.053-0.075] unit increase	N	total lipids in medium HDL measurement	GCST90269663
2	21008652	APOB		Phospholipids to total lipids in very small VLDL percentage (UKB data field 23604)	88,329 European ancestry individuals	rs676210-A	**G > A**	0.205238	3.00E-56	[0.079-0.101] unit decrease	N	phospholipids: total lipids ratio	GCST90269701
2	21008652	APOB		Cholesteryl esters in medium HDL (UKB data field 23569)	88,329 European ancestry individuals	rs676210-A	**G > A**	0.205238	5.00E-50	[0.07-0.091] unit increase	N	cholesteryl ester measurement	GCST90269666

2	21008652	APOB		Phospholipids to total lipids in very large HDL percentage (UKB data field 23629)	88,307 European ancestry individuals	rs676210-A	**G > A**	0.20525	1.00E-12	[0.028-0.049] unit increase	N	phospholipids:total lipids ratio	GCST90269726
2	21008652	APOB		Triglycerides in VLDL (UKB data field 23408)	88,329 European ancestry individuals	rs676210-A	**G > A**	0.205238	5.00E-80	[0.096-0.118] unit decrease	N	triglycerides in VLDL	GCST90269505
2	21008652	APOB		Cholesteryl esters in HDL (UKB data field 23418)	88,329 European ancestry individuals	rs676210-A	**G > A**	0.205238	7.00E-47	[0.066-0.087] unit increase	N	cholesteryl ester measurement	GCST90269515
2	21008652	APOB		Triglycerides to phosphoglycerides ratio (UKB data field 23435)	88,268 European ancestry individuals	rs676210-A	**G > A**	0.205256	4.00E-91	[0.1-0.121] unit decrease	N	triglycerides to phosphoglycerides ratio	GCST90269532
2	21008652	APOB		Docosahexaenoic acid to total fatty acids percentage (UKB data field 23457)	88,268 European ancestry individuals	rs676210-A	**G > A**	0.205256	5.00E-15	[0.033-0.055] unit increase	N	docosahexaenoic acid to total fatty acids	GCST90269554
2	21008652	APOB		Cholesterol in large VLDL (UKB data field 23498)	88,329 European ancestry individuals	rs676210-A	**G > A**	0.205238	##########	[0.11-0.14] unit decrease	N	cholesterol in large VLDL	GCST90269595
2	21008652	APOB		Phospholipids in large VLDL (UKB data field 23497)	88,329 European ancestry individuals	rs676210-A	**G > A**	0.205238	2.00E-93	[0.1-0.13] unit decrease	N	phospholipids in large VLDL measurement	GCST90269594
2	21008652	APOB		Phospholipids in large HDL (UKB data field 23560)	88,329 European ancestry individuals	rs676210-A	**G > A**	0.205238	1.00E-42	[0.061-0.082] unit increase	N	phospholipids in large HDL measurement	GCST90269657
2	21008652	APOB		HDL cholesterol levels (UKB data field 23406)	88,329 European ancestry individuals	rs676210-A	**G > A**	0.205238	1.00E-44	[0.064-0.084] unit increase	N	high density lipoprotein cholesterol measurement	GCST90269503
2	21008652	APOB		Phospholipids in HDL (UKB data field 23414)	88,329 European ancestry individuals	rs676210-A	**G > A**	0.205238	7.00E-32	[0.052-0.073] unit increase	N	phospholipids in HDL	GCST90269511
2	21008652	APOB		Concentration of very large VLDL particles (UKB data field 23488)	88,329 European ancestry individuals	rs676210-A	**G > A**	0.205238	2.00E-78	[0.095-0.117] unit decrease	N	very low density lipoprotein cholesterol measurement	GCST90269585
2	21008652	APOB		Total lipids in very large VLDL (UKB data field 23489)	88,329 European ancestry individuals	rs676210-A	**G > A**	0.205238	2.00E-76	[0.093-0.115] unit decrease	N	total lipids in very large VLDL measurement	GCST90269586
2	21008652	APOB		Phospholipids in very large VLDL (UKB data field 23490)	88,329 European ancestry individuals	rs676210-A	**G > A**	0.205238	1.00E-83	[0.098-0.121] unit decrease	N	phospholipids in very large VLDL	GCST90269587
2	21008652	APOB		Phospholipids in VLDL (UKB data field 23412)	88,329 European ancestry individuals	rs676210-A	**G > A**	0.205238	2.00E-92	[0.11-0.13] unit decrease	N	phospholipids in VLDL	GCST90269509
2	21008652	APOB		Triglycerides in large VLDL (UKB data field 23501)	88,329 European ancestry individuals	rs676210-A	**G > A**	0.205238	4.00E-72	[0.09-0.112] unit decrease	N	triglycerides in large VLDL	GCST90269598
2	21008652	APOB		Average diameter for VLDL particles (UKB data field 23431)	88,329 European ancestry individuals	rs676210-A	**G > A**	0.205238	1.00E-67	[0.085-0.106] unit decrease	N	very low density lipoprotein particle size	GCST90269528
2	21008652	APOB		Concentration of HDL particles (UKB data field 23430)	88,329 European ancestry individuals	rs676210-A	**G > A**	0.205238	5.00E-22	[0.042-0.064] unit increase	N	high density lipoprotein cholesterol measurement	GCST90269527
2	21008652	APOB		Cholesteryl esters in very large VLDL (UKB data field 23492)	88,329 European ancestry individuals	rs676210-A	**G > A**	0.205238	4.00E-96	[0.11-0.13] unit decrease	N	cholesteryl ester measurement	GCST90269589
2	21008652	APOB		Free cholesterol in very large VLDL (UKB data field 23493)	88,329 European ancestry individuals	rs676210-A	**G > A**	0.205238	2.00E-91	[0.1-0.13] unit decrease	N	free cholesterol in very large VLDL	GCST90269590

2	21008652	APOB		Cholesterol in very large VLDL (UKB data field 23491)	88,329 European ancestry individuals	rs676210-A	G > A	0.205238	4.00E-96	[0.11-0.13] unit decrease	N	cholesterol in very large VLDL measurement	GCST90269588
2	21008652	APOB		Triglycerides in very large VLDL (UKB data field 23494)	88,329 European ancestry individuals	rs676210-A	G > A	0.205238	2.00E-63	[0.083-0.105] unit decrease	N	triglycerides in very large VLDL measurement	GCST90269591
2	21008652	APOB		Triglycerides in medium VLDL (UKB data field 23508)	88,329 European ancestry individuals	rs676210-A	G > A	0.205238	3.00E-94	[0.11-0.13] unit decrease	N	triglycerides in medium VLDL measurement	GCST90269605
2	21008652	APOB		Free cholesterol in HDL (UKB data field 23422)	88,329 European ancestry individuals	rs676210-A	G > A	0.205238	2.00E-34	[0.054-0.074] unit increase	N	free cholesterol in HDL	GCST90269519
2	21008652	APOB		Apolipoprotein A1 levels (UKB data field 23440)	88,329 European ancestry individuals	rs676210-A	G > A	0.205238	1.00E-28	[0.049-0.07] unit increase	N	apolipoprotein A 1	GCST90269537
2	21008652	APOB		Cholesteryl esters in chylomicrons and extremely large VLDL (UKB data field	88,329 European ancestry individuals	rs676210-A	G > A	0.205238	8.00E-87	[0.1-0.12] unit decrease	N	cholesteryl ester measurement	GCST90269582
2	21008652	APOB		Cholesterol in chylomicrons and extremely large VLDL (UKB data field 23484)	88,329 European ancestry individuals	rs676210-A	G > A	0.205238	4.00E-82	[0.098-0.12] unit decrease	N	cholesterol in chylomicrons and extremely large VLDL	GCST90269581
2	21008652	APOB		Triglycerides in chylomicrons and extremely large VLDL (UKB data field 23487)	88,329 European ancestry individuals	rs676210-A	G > A	0.205238	1.00E-41	[0.065-0.088] unit decrease	N	triglycerides in chylomicrons and extremely large VLDL	GCST90269584
2	21008652	APOB		Free cholesterol in chylomicrons and extremely large VLDL (UKB data field 23486)	88,329 European ancestry individuals	rs676210-A	G > A	0.205238	3.00E-74	[0.092-0.114] unit decrease	N	free cholesterol in chylomicrons and extremely	GCST90269583
2	21008652	APOB		Cholesteryl esters in large VLDL (UKB data field 23499)	88,329 European ancestry individuals	rs676210-A	G > A	0.205238	##########	[0.12-0.14] unit decrease	N	cholesteryl ester measurement	GCST90269596
2	21008652	APOB		Free cholesterol in large VLDL (UKB data field 23500)	88,329 European ancestry individuals	rs676210-A	G > A	0.205238	1.00E-94	[0.11-0.13] unit decrease	N	free cholesterol in large VLDL measurement	GCST90269597
2	21008652	APOB		Free cholesterol in medium HDL (UKB data field 23570)	88,329 European ancestry individuals	rs676210-A	G > A	0.205238	1.00E-37	[0.058-0.079] unit increase	N	free cholesterol in medium HDL measurement	GCST90269667
2	21029662	APOB		LDL cholesterol levels	12,685 Chinese ancestry individuals	rs13306194-A	G > A	0.128	1.00E-12	[0.091-0.169] unit decrease	N	LDL cholesterol change	GCST009917
2	21029662	APOB		Total cholesterol levels	12,685 Chinese ancestry individuals	rs13306194-A	G > A	0.128	6.00E-12	[0.091-0.169] unit decrease	N	total cholesterol measurement	GCST009919
2	21029662	APOB		Total cholesterol levels	6,949 Korean ancestry individuals	rs13306194-A	G > A	0.124	4.00E-06	[2.41-5.97] unit decrease	N	total cholesterol measurement	GCST007441
2	21029662	APOB		Apolipoprotein C-III levels	9,947 Han Chinese ancestry individuals, 294 individuals	rs13306194-A	G > A	NR	7.00E-09	[0.091-0.169] unit decrease	N	level of apolipoprotein C-III in blood	GCST90319526
2	21029662	APOB		Triglyceride levels	9,947 Han Chinese ancestry individuals, 294	rs13306194-A	G > A	NR	2.00E-09	[0.1-0.18] unit decrease	N	triglyceride measurement	GCST90319535
2	21029662	APOB		Low density lipoprotein cholesterol levels	146,492 East Asian ancestry individuals	rs13306194-A	G > A	0.114019	9.00E-51	[0.11-0.14] unit decrease	N	low density lipoprotein cholesterol measurement	GCST90239657
2	21029662	APOB		Triglyceride levels	146,492 East Asian ancestry individuals	rs13306194-A	G > A	0.113994	3.00E-18	[0.056-0.087] unit decrease	N	triglyceride measurement	GCST90239663
2	21029662	APOB		Triglyceride levels	58,701 Korean ancestry individuals	rs13306194-A	G > A	NR	1.00E-13	[3.33-5.71] unit decrease	N	triglyceride measurement	GCST90134521

2	2.32E+08	ALPP		Refractive error	51,624 European ancestry individuals	rs1130335-T	C > T	NR	2.00E-10	[0.048-0.092] unit increase	N	refractive error	GCST90104407
3	38362981	XYLB		Protein quantitative trait loci (liver)	172 European ancestry individuals, 29 Black individuals, 86 individuals	rs17118-A	C > A	0.3118	1.00E-11	NR unit decrease	N	protein measurement	GCST011427
3	43056273	GASK1A		Height	253,288 European ancestry individuals	rs3732858-A	G > A	0.17614388	7.00E-07		N	body height	GCST005908
3	43056273	GASK1A		Height	5,314,291 European ancestry, Hispanic or Latin American, East Asian ancestry, African ancestry, South Asian ancestry	rs3732858-A	G > A	0.1654	##########	[0.019-0.022] unit decrease	N	body height	GCST90245848
3	47240813	KIF9-AS1, KIF9		Adiponectin levels	up to 60,465 European ancestry individuals, up to 2,568 East Asian ancestry individuals, up to 3,271 African American individuals, up to 1,435 Hispanic individuals	rs2276853-A	G > A	0.59433962	2.00E-07	unit decrease	N	adiponectin measurement	GCST010050
3	47240813	KIF9-AS1, KIF9		Type 2 diabetes (adjusted for BMI)	48,286 European ancestry cases, 250,671 European ancestry controls, 33,126 African American, East Asian, Hispanic/Latino or South Asian cases, 120,161 African American, East Asian, Hispanic/Latino or South Asian controls	rs2276853-A	G > A	0.588	5.00E-08	[1.02-1.05]	N	type 2 diabetes mellitus	GCST007516
3	47240813	KIF9-AS1, KIF9		Waist-to-hip ratio adjusted for BMI (additive genetic model)	288,492 European ancestry individuals, up to 15,687 African American individuals, up to 29,315 South Asian ancestry individuals, up to 6,800 East Asian ancestry individuals, up to 4,075 Hispanic ancestry	rs2276853-A	G > A	0.5899	3.00E-07	[0.0082-0.018] unit increase	N	BMI-adjusted waist-hip ratio	GCST007483
4	1.25E+08	FAT4		Primary open angle glaucoma (multi-trait analysis)	at least 29,241 European ancestry cases, at least 350,181 European ancestry controls (MTAG boosted by vertical cup-to-disc ratio and intraocular pressure samples), 6,935 Asian ancestry cases, 39,588 Asian ancestry controls, 3,281 African ancestry cases, 2,791 African	rs1567047-A	G > A	NR	1.00E-09	[0.035-0.068] unit decrease	N	open-angle glaucoma	GCST90310211
5	1.41E+08	WDR55		Systolic blood pressure	1,164,961 European ancestry individuals	rs2286394-T	C > T	0.2329	3.00E-08	[0.09-0.194] mmHg increase	N	systolic blood pressure	GCST90292477
5	1.41E+08	WDR55		Systolic blood pressure	810,865 European ancestry individuals	rs2286394-T	C > T	0.2295	3.00E-08	[0.12-0.24] mmHg increase	N	systolic blood pressure	GCST90000062
5	1.41E+08	WDR55		Pulse pressure	1,164,961 European ancestry individuals	rs2286394-T	C > T	0.2333	7.00E-09	[0.069-0.142] mmHg increase	N	pulse pressure measurement	GCST90292476

5	1.41E+08	WDR55		Pulse pressure	810,865 European ancestry individuals	rs2286394-T	C > T	0.2296	9.00E-08	[0.075-0.163] mmHg increase	N	pulse pressure measurement	GCST90000061
5	35965868	UGT3A1		Urine GlcNAc sulfate conjugate of C21H34O2 steroid** levels in chronic kidney disease	4,609 European ancestry individuals	rs3756669-C	A > C	0.12	##########	[0.78-0.92] unit decrease	N	metabolite measurement	GCST90265175
5	35965868	UGT3A1		Urine N-acetylglucosamine conjugate of C24H38O4 bile acid** levels in chronic kidney	3,793 European ancestry individuals	rs3756669-C	A > C	0.11	2.00E-42	[0.45-0.61] unit decrease	N	metabolite measurement	GCST90265562
7	12367363	VWDE		Early-onset ischemic stroke	330 African American cases, 393 European ancestry cases, 298 African American controls, 428 European ancestry controls	rs6460939-G	C > G	0.55	9.00E-06		N	Ischemic stroke	GCST010652
7	1.3E+08	ZC3HC1, UBE2H-DT		Eosinophil counts	583,850 African American or Afro-Caribbean, African ancestry, European ancestry, East Asian ancestry, Hispanic or Latin American and South Asian	rs11556924-T	C > T	0.324113	1.00E-15		N	eosinophil count	GCST90002302
7	1.3E+08	ZC3HC1, UBE2H-DT		Hematocrit	737,823 African American or Afro-Caribbean, African ancestry, European ancestry, East Asian ancestry, Hispanic or Latin American and South Asian	rs11556924-T	C > T	0.304231	9.00E-12		N	hematocrit	GCST90002308
7	1.3E+08	ZC3HC1, UBE2H-DT		Hemoglobin concentration	563,946 European ancestry individuals	rs11556924-T	C > T	0.384498	2.00E-16	[0.012-0.02] SD unit decrease	N	hemoglobin measurement	GCST90002310
7	1.3E+08	ZC3HC1, UBE2H-DT		Hematocrit	408,112 British individuals	rs11556924-T	C > T	0.388975	2.00E-11	[0.011-0.02] unit decrease	N	hematocrit	GCST90002383
7	1.3E+08	ZC3HC1, UBE2H-DT		Hemoglobin concentration	746,431 African American or Afro-Caribbean, African ancestry, European ancestry, East Asian ancestry, Hispanic or Latin American and South Asian	rs11556924-T	C > T	0.302057	2.00E-15		N	hemoglobin measurement	GCST90002314
7	1.3E+08	ZC3HC1, UBE2H-DT		Hemoglobin	408,112 British individuals	rs11556924-T	C > T	0.389006	2.00E-14	[0.013-0.022] unit decrease	N	hemoglobin measurement	GCST90002384
7	1.3E+08	ZC3HC1, UBE2H-DT		Neutrophil count	519,288 European ancestry individuals	rs11556924-T	C > T	0.385273	2.00E-14	[0.011-0.019] SD unit increase	N	neutrophil count	GCST90002351
7	1.3E+08	ZC3HC1, UBE2H-DT		Parental lifespan	at least 500,193 European ancestry offspring	rs11556924-T	C > T	0.386	4.00E-07	[0.012-0.028] unit increase	N	parental longevity	GCST009890
7	1.3E+08	ZC3HC1, UBE2H-DT		Hemoglobin levels	684,122 European ancestry individuals	rs11556924-T	C > T	NR	8.00E-12	[NR] unit decrease	N	hemoglobin measurement	GCST010083
7	1.3E+08	ZC3HC1, UBE2H-DT		Diastolic blood pressure	up to 201,529 European ancestry individuals	rs11556924-T	C > T	0.384	8.00E-15	[0.16-0.27] mm Hg decrease	N	diastolic blood pressure	GCST006258
7	1.3E+08	ZC3HC1, UBE2H-DT		Systolic blood pressure	up to 201,529 European ancestry individuals	rs11556924-T	C > T	0.384	8.00E-09	[0.18-0.36] mm Hg decrease	N	systolic blood pressure	GCST006259
7	1.3E+08	ZC3HC1, UBE2H-DT		Plateletcrit	164,339 European ancestry individuals	rs11556924-T	C > T	0.3885	4.00E-09	[0.015-0.029] unit increase	N	platelet crit	GCST004607
7	1.3E+08	ZC3HC1, UBE2H-DT		White blood cell count (basophil)	171,846 European ancestry individuals	rs11556924-T	C > T	0.3882	1.00E-11	[0.017-0.031] unit increase	N	basophil count	GCST004618
7	1.3E+08	ZC3HC1, UBE2H-DT		Systolic blood pressure	150,134 European ancestry individuals	rs11556924-T	C > T	0.357	6.00E-06	[0.2-0.51] unit decrease	N	systolic blood pressure	GCST004776

7	1.3E+08	ZC3HC1, UBE2H-DT		Neutrophil count	408,112 British individuals	rs11556924-T	C > T	0.388961	1.00E-12	[0.012-0.021] unit increase	N	neutrophil count	GCST90002398
7	1.3E+08	ZC3HC1, UBE2H-DT		White blood cell count	562,243 European ancestry individuals	rs11556924-T	C > T	0.384493	3.00E-20	[0.014-0.021] SD unit increase	N	myeloid white cell count	GCST90002374
7	1.3E+08	ZC3HC1, UBE2H-DT		Platelet count	166,066 European ancestry individuals	rs11556924-T	C > T	0.3886	2.00E-12	[0.019-0.034] unit increase	N	platelet count	GCST004603
7	1.3E+08	ZC3HC1, UBE2H-DT		White blood cell count	408,112 British individuals	rs11556924-T	C > T	0.388957	4.00E-14	[0.013-0.022] unit increase	N	leukocyte count	GCST90002407
7	1.3E+08	ZC3HC1, UBE2H-DT		Total testosterone levels	425,097 European ancestry individuals	rs11556924-T	C > T	0.389328	1.00E-09	[0.0056-0.0108] unit increase	N	testosterone measurement	GCST90012114
7	1.3E+08	ZC3HC1, UBE2H-DT		Sex hormone-binding globulin levels adjusted for BMI	368,929 European ancestry individuals	rs11556924-T	C > T	0.389294	5.00E-18	[0.006-0.0094] unit increase	N	sex hormone-binding globulin	GCST90012110
7	1.3E+08	ZC3HC1, UBE2H-DT		Sex hormone-binding globulin levels	370,125 European ancestry individuals	rs11556924-T	C > T	0.389294	2.00E-19	[0.0069-0.0107] unit increase	N	sex hormone-binding globulin	GCST90012111
7	1.3E+08	ZC3HC1, UBE2H-DT		Red cell distribution width	408,112 British individuals	rs11556924-T	C > T	0.389025	1.00E-13	[0.012-0.021] unit increase	N	obsolete_red blood cell distribution	GCST90002404
7	1.3E+08	ZC3HC1, UBE2H-DT		Coronary artery disease	up to 122,733 cases, up to 424,528 controls	rs11556924-T	C > T	0.3624	1.00E-23	[0.044-0.066] unit decrease	N	coronary artery disease	GCST005195
7	1.3E+08	ZC3HC1, UBE2H-DT		Coronary artery disease	88,192 cases, 162,544 controls	rs11556924-T	C > T	0.3761	2.00E-18	[0.045-0.07] unit decrease	N	coronary artery disease	GCST005196
7	1.3E+08	ZC3HC1, UBE2H-DT		Coronary artery disease	88,192 cases, 162,544 controls	rs11556924-T	C > T	0.36	1.00E-24	[0.05-0.074] unit decrease	N	coronary artery disease	GCST005196
7	1.3E+08	ZC3HC1, UBE2H-DT		Coronary artery disease	34,541 cases, 261,984 controls	rs11556924-T	C > T	0.36	1.00E-24	[0.05-0.074] unit decrease	N	coronary artery disease	GCST005194
7	1.3E+08	ZC3HC1, UBE2H-DT		White blood cell count	746,667 African American or Afro-Caribbean, African ancestry, European ancestry, East Asian ancestry, Hispanic or Latin American and South Asian [illegible]	rs11556924-T	C > T	0.301138	2.00E-18		N	myeloid white cell count	GCST90002378
7	1.3E+08	ZC3HC1, UBE2H-DT		Sex hormone-binding globulin levels	196,901 European ancestry individuals	rs11556924-T	C > T	0.375757	1.00E-12	[0.017-0.03] unit increase	N	sex hormone-binding globulin	GCST90104273
7	1.3E+08	ZC3HC1, UBE2H-DT		Sex hormone-binding globulin levels in postmenopausal women	92,911 European ancestry individuals	rs11556924-T	C > T	0.375757	2.00E-07	[0.015-0.034] unit increase	N	sex hormone-binding globulin	GCST90104277
7	1.3E+08	ZC3HC1, UBE2H-DT		Sex hormone-binding globulin levels adjusted for BMI	188,908 European ancestry women	rs11556924-T	C > T	0.389097	3.00E-18	[0.0088-0.0138] unit increase	N	sex hormone-binding globulin	GCST90012106
7	1.3E+08	ZC3HC1, UBE2H-DT		Sex hormone-binding globulin levels	189,473 European ancestry women	rs11556924-T	C > T	0.389097	6.00E-16	[0.0093-0.015] unit increase	N	sex hormone-binding globulin	GCST90012107
7	1.3E+08	ZC3HC1, UBE2H-DT		Medication use (calcium channel blockers)	31,904 European ancestry cases, 172,474 European ancestry controls	rs11556924-T	C > T	0.389692	5.00E-09	[0.032-0.064] unit decrease	N	Calcium channel blocker use	GCST007929
7	1.3E+08	ZC3HC1, UBE2H-DT		Medication use (agents acting on the renin-angiotensin system)	62,752 European ancestry cases, 174,778 European ancestry controls	rs11556924-T	C > T	0.389132	3.00E-09	[0.024-0.049] unit decrease	N	Agents acting on the renin-angiotensin system use [illegible]	GCST007930
7	1.3E+08	ZC3HC1, UBE2H-DT		Cortical thickness	35,657 White British ancestry individuals	rs11556924-T	C > T	0.3866	4.00E-08		N	cortical thickness	GCST90091061

7	1.3E+08	ZC3HC1, UBE2H-DT		Estimated glomerular filtration rate (creatinine)	1,205,871 European ancestry individuals, 168,300 East Asian ancestry individuals, 63,553 African ancestry individuals, 23,509 Hispanic or Latin American individuals, 22,103 African American individuals, 21,791 Central Asian or South Asian ancestry individuals, 1,502 Middle Eastern ancestry individuals, 939 other admixed ancestry individuals, 602 Native American ancestry	rs11556924-T	C > T	0.3609	9.00E-20	z score increase	N	glomerular filtration rate	GCST90100220
7	1.3E+08	ZC3HC1, UBE2H-DT		Diastolic blood pressure	340,162 European ancestry individuals, 145,515 East Asian ancestry individuals	rs11556924-T	C > T	NR	7.00E-13	[0.012-0.022] unit decrease	N	diastolic blood pressure	GCST90018952
7	1.3E+08	ZC3HC1, UBE2H-DT		Systolic blood pressure	1,028,980 European ancestry individuals	rs11556924-T	C > T	NR	3.00E-12	[0.13-0.22] unit decrease	N	systolic blood pressure	GCST90310294
7	1.3E+08	ZC3HC1, UBE2H-DT		Vertex-wise sulcal depth	33,748 European ancestry individuals	rs11556924-T	C > T	0.38	1.00E-12	z score increase	N	brain measurement	GCST90095129
7	1.3E+08	ZC3HC1, UBE2H-DT		Hemoglobin	350,474 European ancestry individuals, 152,447 East Asian ancestry individuals	rs11556924-T	C > T	NR	5.00E-13	[0.01-0.018] unit decrease	N	hemoglobin measurement	GCST90018957
7	1.3E+08	ZC3HC1, UBE2H-DT		Diastolic blood pressure	1,028,980 European ancestry individuals	rs11556924-T	C > T	NR	2.00E-27	[0.14-0.2] unit decrease	N	diastolic blood pressure	GCST90310295
7	1.3E+08	ZC3HC1, UBE2H-DT		Diastolic blood pressure	526,001 European ancestry individuals	rs11556924-T	C > T	NR	7.00E-15	[0.13-0.21] unit decrease	N	diastolic blood pressure	GCST90132904
7	1.3E+08	ZC3HC1, UBE2H-DT		Systolic blood pressure	526,001 European ancestry individuals	rs11556924-T	C > T	NR	3.00E-09	[0.15-0.3] unit decrease	N	systolic blood pressure	GCST90132903
7	1.3E+08	ZC3HC1, UBE2H-DT		Hematocrit	350,475 European ancestry individuals, 153,015 East Asian ancestry individuals	rs11556924-T	C > T	NR	1.00E-11	[0.0094-0.0172] unit decrease	N	hematocrit	GCST90018960
7	1.3E+08	ZC3HC1, UBE2H-DT		Coronary artery disease	181,522 European ancestry, unknown cases, 984,168 European ancestry, unknown [illegible]	rs11556924-T	C > T	0.355	4.00E-21	[0.942-0.961]	N	coronary artery disease	GCST90132314
7	1.3E+08	ZC3HC1, UBE2H-DT		Smoking initiation	2,669,029 European ancestry individuals	rs11556924-T	C > T	0.381	3.00E-11	[0.0042-0.0077] unit decrease	N	smoking initiation	GCST90243968
7	1.3E+08	ZC3HC1, UBE2H-DT		Smoking initiation	3,382,012 European ancestry, East Asian ancestry, Hispanic or Latin American, African ancestry [illegible]	rs11556924-T	C > T	0.332	1.00E-13	[0.0044-0.0077] unit decrease	N	smoking initiation	GCST90243985
7	1.3E+08	ZC3HC1, UBE2H-DT		Hemoglobin concentration	52,141 European ancestry females	rs11556924-T	C > T	0.3892	1.00E-11	unit decrease	N	hemoglobin measurement	GCST90258653
7	1.3E+08	ZC3HC1, UBE2H-DT		Height	5,314,291 European ancestry, Hispanic or Latin American, East Asian ancestry, African ancestry, South Asian ancestry [illegible]	rs11556924-T	C > T	0.3322	1.00E-59	[0.0096-0.0124] unit increase	N	body height	GCST90245848
7	36399100	ANLN		Total cholesterol levels	1,320,016 European ancestry individuals	rs3735400-G	C > G	0.117031	5.00E-12	[0.013-0.021] unit decrease	N	total cholesterol measurement	GCST90239676

8	17868560	FGL1		Lactotransferrin levels	2,935 Qatari ancestry individuals	rs2653414-A	C > A	0.0408859	1.00E-11	[illegible]	[0.33-0.6] unit increase	N	lactotransferrin measurement	GCST90161477
8	17868560	FGL1		Preeclampsia or eclampsia	3,223 Asian ancestry cases, 96,119 Asian ancestry controls, 20 African ancestry cases, 1,406 African ancestry controls, 14 Hispanic or Latin American cases, 2,457 Hispanic or Latin American controls, 1,389 European ancestry cases, 351,259 European ancestry controls	rs2653414-A	C > A	NR	3.00E-11	[illegible]		N	preeclampsia	GCST90271316
8	17868560	FGL1		Fibrinogen-like protein 1 levels	10,708 European ancestry individuals	rs2653414-A	C > A	0.01	#########	[illegible]	[1.8-2.01] unit decrease	N	protein measurement	GCST90247601
8	97931370	MATN2		Height	5,314,291 European ancestry, Hispanic or Latin American, East Asian ancestry, African ancestry, South Asian ancestry individuals	rs2290472-T	C > T	0.1677	7.00E-40	[illegible]	[0.0093-0.0125] unit decrease	N	body height	GCST90245848
9	1.05E+08	NIPSNAP 3B		Metabolite levels (allo-isoleucine	1,684 European, Hispanic or African American individuals	rs10761084-C	G > C	0.1609	4.00E-21	[illegible]	[0.36-0.55] unit decrease	N	level of L-alloisoleucine in blood	GCST90299904
9	1.3E+08	TOR1A		Smoking initiation	3,382,012 European ancestry, East Asian ancestry, Hispanic or Latin American, African ancestry individuals	rs1801968-G	C > G	0.128	2.00E-12	[illegible]	[0.0058-0.0103] unit decrease	N	smoking initiation	GCST90243985
9	1.3E+08	TOR1A		Height	5,314,291 European ancestry, Hispanic or Latin American, East Asian ancestry, African ancestry, South Asian ancestry individuals	rs1801968-G	C > G	0.1259	3.00E-12	[illegible]	[0.0048-0.0084] unit increase	N	body height	GCST90245848
9	34107507	DCAF12		Monocyte count	521,594 European ancestry individuals	rs11557154-T	C > T	0.127576	3.00E-30	[illegible]	[0.027-0.037] SD unit increase	N	monocyte count	GCST90002340
9	34107507	DCAF12		Monocyte percentage of white cell	408,112 British individuals	rs11557154-T	C > T	0.127343	4.00E-22	[illegible]	[0.026-0.039] unit increase	N	monocyte percentage of	GCST90002394
9	34107507	DCAF12		Monocyte count	639,696 African American or Afro-Caribbean, African ancestry, European ancestry, East Asian ancestry, Hispanic or Latin American and South Asian ancestry individuals	rs11557154-T	C > T	0.185547	8.00E-32	[illegible]		N	monocyte count	GCST90002344
9	34107507	DCAF12		Monocyte count	408,112 British individuals	rs11557154-T	C > T	0.12733	5.00E-24	[illegible]	[0.027-0.04] unit increase	N	monocyte count	GCST90002393
9	34107507	DCAF12		Neutrophil percentage of white cel	171,542 European ancestry individuals	rs11557154-T	C > T	0.1277	2.00E-09	[illegible]	[0.021-0.042] unit decrease	N	neutrophil percentage of	GCST004633
9	34107507	DCAF12		Alanine aminotransferase levels	390,812 European ancestry individuals	rs11557154-T	C > T	0.13	3.00E-09	[illegible]	z-score increase	N	serum alanine aminotransferase measurement	GCST90011898
9	34107507	DCAF12		Estimated glomerular filtration rat	342,376 European ancestry individuals, 6,016 African ancestry individuals, 7,339 South Asian ancestry individuals	rs11557154-T	C > T	NR	2.00E-10	[illegible]	[0.016-0.03] unit increase	N	glomerular filtration rate	GCST90019506

9	34107507	DCAF12	Creatinine levels	342,376 European ancestry individuals, 6,016 African ancestry individuals, 7,339 South Asian ancestry individuals	rs11557154-T	C > T	NR	1.00E-10	[0.016-0.03] unit decrease	N	creatinine measurement	GCST90019502
9	34107507	DCAF12	Serum alkaline phosphatase levels	342,535 European ancestry individuals, 6,019 African ancestry individuals, 7,337 South Asian ancestry individuals	rs11557154-T	C > T	NR	1.00E-12	[0.018-0.032] unit decrease	N	alkaline phosphatase measurement	GCST90019494
9	34107507	DCAF12	Aspartate aminotransferase to alanine aminotra	341,165 European ancestry individuals, 5,981 African ancestry individuals, 7,309 South Asian ancestry individuals	rs11557154-T	C > T	NR	5.00E-21	[0.027-0.041] unit decrease	N	aspartate aminotransferase to alanine aminotransferase ratio	GCST90019498
9	34107507	DCAF12	Alanine aminotransferase levels	342,387 European ancestry individuals, 6,017 African ancestry individuals, 7,325 South Asian ancestry individuals	rs11557154-T	C > T	NR	1.00E-09	[0.015-0.029] unit increase	N	serum alanine aminotransferase measurement	GCST90019492
9	34107507	DCAF12	Serum total protein levels	313,032 European ancestry individuals, 5,573 African ancestry individuals, 6,687 South Asian ancestry individuals	rs11557154-T	C > T	NR	5.00E-10	[0.016-0.03] unit decrease	N	blood protein measurement	GCST90019522
9	34107507	DCAF12	Insulin-like growth factor 1 levels	340,567 European ancestry individuals, 5,974 African ancestry individuals, 7,283 South Asian ancestry individuals	rs11557154-T	C > T	NR	4.00E-10	[0.015-0.029] unit increase	N	IGF-1 measurement	GCST90019511
9	34107507	DCAF12	Non-albumin protein levels	313,032 European ancestry individuals, 5,573 African ancestry individuals, 6,687 South Asian ancestry individuals	rs11557154-T	C > T	NR	1.00E-12	[0.019-0.034] unit decrease	N	serum non-albumin protein measurement	GCST90019515
9	34107507	DCAF12	Serum creatinine levels	344,104 European ancestry individuals, 150,266 East Asian ancestry individuals	rs11557154-T	C > T	NR	3.00E-14	[0.012-0.019] unit decrease	N	creatinine measurement	GCST90018979
9	34107507	DCAF12	Monocyte count	349,856 European ancestry individuals, 95,119 East Asian ancestry individuals	rs11557154-T	C > T	NR	5.00E-21	[0.02-0.031] unit increase	N	monocyte count	GCST90018967
9	34107507	DCAF12	Mean corpuscular hemoglobin	350,472 European ancestry individuals, 128,028 East Asian ancestry individuals	rs11557154-T	C > T	NR	2.00E-17	[0.017-0.027] unit increase	N	mean corpuscular hemoglobin	GCST90018964
9	34107507	DCAF12	Colorectal cancer	21,731 European ancestry cases, 47,444 European ancestry controls, 78,473 East Asian ancestry cases, 107,143 East Asian ancestry controls	rs11557154-T	C > T	NR	6.00E-10	[0.036-0.069] unit increase	N	colorectal cancer	GCST90129505
9	34107507	DCAF12	Aldo-keto reductase family 1 member C	10,708 European ancestry individuals	rs11557154-T	C > T	0.13	2.00E-28	[0.18-0.26] unit increase	N	protein measurement	GCST90246471

9	34107507	DCAF12		Height	5,314,291 European ancestry, Hispanic or Latin American, East Asian ancestry, African ancestry, South Asian ancestry	rs11557154-T	C > T	0.1604	2.00E-25	[0.0077-0.0113] unit decrease	N	body height	GCST90245848
9	95928855	ERCC6L2		Height	5,314,291 European ancestry, Hispanic or Latin American, East Asian ancestry, African ancestry, South Asian ancestry individuals	rs2274654-C	T > C	0.1709	2.00E-27	[0.0073-0.0105] unit increase	N	body height	GCST90245848
10	1.22E+08	ARMS2		Age-related macular degeneration	893 European ancestry cases, 2,199 European ancestry controls	rs10490924-T	G > T	0.21	3.00E-72	[2.64-3.40]	N	age-related macular degeneration	GCST001571
10	1.22E+08	ARMS2		Age-related macular degeneration	6,713 European ancestry cases, 48,402 European ancestry contols, 110 Southern Indian ancestry cases, 119 Southern Indian ancestry controls, 827 Japanese ancestry cases,	rs10490924-T	G > T	0.3	4E-540	[2.72-2.80]	N	age-related macular degeneration	GCST001884
10	1.22E+08	ARMS2		Age-related macular degeneration (choroidal neovascularisation)	1,775 European ancestry choroidal neovascularization cases, 4,134 European ancestry [illegible]	rs10490924-T	G > T	0.206	##########	[3.33-4.05]	N	wet macular degeneration	GCST001579
10	1.22E+08	ARMS2		Age-related macular degeneration (geographic atrophy)	819 European ancestry geographic atrophy cases, 4,134 European ancestry controls	rs10490924-T	G > T	0.206	7.00E-47	[2.24-2.83]	N	atrophic macular degeneration	GCST001578
10	1.22E+08	ARMS2		Age-related macular degeneration (CNV vs. GA)	1,775 European ancestry choroidal neovascularization cases, 819 European ancestry [illegible]	rs10490924-T	G > T	0.438	7.00E-14	[NR]	N	atrophic macular degeneration, wet macular [illegible]	GCST001577
10	1.22E+08	ARMS2		Age-related macular degeneration	684 European ancestry cases, 188 European	rs10490924-T	G > T	NR	1.00E-60	[NR]	N	age-related macular	GCST000806
10	1.22E+08	ARMS2		Age-related macular degeneration	2,594 European ancestry cases, 4,134 European ancestry controls	rs10490924-T	G > T	0.21	4E-322	[NR]	N	age-related macular degeneration	GCST001100
10	1.22E+08	ARMS2		Refractive error	51,624 European ancestry individuals	rs10490924-T	G > T	NR	4.00E-08	[0.026-0.054] unit decrease	N	refractive error	GCST90104407
10	1.22E+08	ARMS2		Age-related macular degeneration	339 Han Chinese ancestry cases, 3,390 Han Chinese ancestry controls	rs10490924-T	G > T	NR	9.00E-12		N	age-related macular degeneration	GCST90271581
10	1.25E+08	CTBP2		Body mass index	up to 449,889 European ancestry individuals, up to 29,398 South Asian ancestry individuals, up to 27,610 African American individuals, up to 8,839 East Asian individuals, up to 10,772 Hispanic ancestry individuals	rs2946994-G	G > C	0.508	2.00E-08	[0.009-0.0184] unit increase	N	body mass index	GCST008129
10	20868692	NEBL		Atrial fibrillation	8,180 Japanese ancestry cases, 28,612 Japanese ancestry controls	rs2296610-T	G > T	0.125	2.00E-14	[1.141-1.251]	N	atrial fibrillation	GCST004373

10	20868692	NEBL		Arrhythmia	17,861 Japanese ancestry cases, 194,592 Japanese ancestry controls	rs2296610-T	G > T	0.12324611	1.00E-09	[1.073316908-1.147880301]	N	cardiac arrhythmia	GCST90013707
10	20868692	NEBL		Atrial fibrillation/atrial flutter	29,212 European ancestry cases, 400,539 European ancestry controls, 4,150 East Asian ancestry cases, 155,540 East Asian	rs2296610-T	G > T	NR	1.00E-08	[0.1-0.21] unit increase	N	atrial fibrillation	GCST90018796
10	73647154	SYNPO2L		Mean arterial pressure	120,473 European ancestry individuals, 21,503 African American individuals, 4,586	rs34163229-T	G > T	0.15198632	6.00E-06	[0.18-0.45] unit increase	N	mean arterial pressure	GCST006231
10	73647154	SYNPO2L		Heart failure	90,653 European ancestry cases, 1,188,957 European ancestry controls	rs34163229-T	G > T	NR	7.00E-13	[0.04-0.072] unit decrease	N	heart failure	GCST90274223
10	73647154	SYNPO2L		Left ventricular end diastole inferior wall thickness	42,122 European ancestry individuals	rs34163229-T	G > T	NR	1.00E-09	[0.04-0.08] unit increase	N	left ventricular structural measurement	GCST90278515
10	98257696	LOXL4		Height	253,288 European ancestry individuals	rs1983864-G	T > G	0.34012166	6.00E-06		N	body height	GCST005908
10	98257696	LOXL4		Diastolic blood pressure	1,028,980 European ancestry individuals	rs1983864-G	T > G	NR	1.00E-06	[0.046-0.107] unit increase	N	diastolic blood pressure	GCST90310295
10	98257696	LOXL4		Height	455,180 Hispanic or Latin American individuals	rs1983864-G	T > G	0.4238	1.00E-15	[0.013-0.022] unit increase	N	body height	GCST90245844
10	98257696	LOXL4		Pulse pressure	1,028,980 European ancestry individuals	rs1983864-G	T > G	NR	5.00E-10	[0.077-0.147] unit decrease	N	pulse pressure measurement	GCST90310296
10	98257696	LOXL4		Body mass index	1,122,049 European ancestry individuals	rs1983864-G	T > G	NR	3.00E-29	[0.013-0.018] unit decrease	N	body mass index	GCST90255621
10	98257696	LOXL4		Body mass index	342,566 European ancestry individuals	rs1983864-G	T > G	NR	7.00E-12	[0.012-0.022] unit decrease	N	body mass index	GCST90428119
10	98257696	LOXL4		Height	5,314,291 European ancestry, Hispanic or Latin American, East Asian ancestry, African ancestry, South Asian ancestry	rs1983864-G	T > G	0.3521	##########	[0.015-0.018] unit increase	N	body height	GCST90245848
11	1.18E+08	JAML		Junctional adhesion molecule-like levels (AMICA1.8232.90.3)	3,301 European ancestry individuals	rs17121881-T	A > T	0.583	6E-667	[0.96-1.04] unit increase	N	junctional adhesion molecule-like	GCST90241666
11	8640969	TRIM66		Obesity	93,015 European ancestry overweight individuals, 32,858 European ancestry class I obese individuals, 9,889 European ancestry class II obese individuals, 2,896 European ancestry class III obese individuals, up to 65,840 European ancestry controls	rs11042023-C	T > C	0.65	1.00E-11	[NR]	N	obesity	GCST001953

11	8640969	TRIM66		Body mass index	up to 449,889 European ancestry individuals, up to 29,398 South Asian ancestry individuals, up to 27,610 African American individuals, up to 8,839 East Asian individuals, up to 10,772 Hispanic ancestry individuals	rs11042023-C	T > C	0.6395	1.00E-16	[0.012-0.019] unit increase	N	body mass index	GCST008129
12	1.08E+08	WSCD2		Predicted visceral adipose tissue	325,153 British ancestry individuals	rs3764002-T	C > T	0.261	2.00E-09	[0.011-0.022] unit decrease	N	visceral adipose tissue	GCST008744
12	1.08E+08	WSCD2		Appendicular lean mass	181,862 European ancestry elderly individuals	rs3764002-T	C > T	0.262461	3.00E-21	[0.058-0.088] unit increase	N	appendicular lean mass	GCST009577
12	1.08E+08	WSCD2		Waist-to-hip ratio adjusted for BM	335,660 European ancestry individuals, 69,909 individuals	rs3764002-T	C > T	NR	5.00E-11	[0.011-0.023] unit decrease	N	BMI-adjusted waist-hip ratio	GCST010926
12	1.08E+08	WSCD2		Appendicular lean mass	450,243 European ancestry individuals	rs3764002-T	C > T	0.2617	4.00E-39	[0.024-0.032] unit increase	N	appendicular lean mass	GCST90000025
12	1.08E+08	WSCD2		Appendicular lean mass	244,730 European ancestry women	rs3764002-T	C > T	0.262	1.00E-23	[0.024-0.036] unit increase	N	appendicular lean mass	GCST90000027
12	1.08E+08	WSCD2		Appendicular lean mass	205,513 European ancestry men	rs3764002-T	C > T	0.262	7.00E-19	[0.024-0.036] unit increase	N	appendicular lean mass	GCST90000026
12	1.08E+08	WSCD2		A body shape index	219,872 British ancestry women	rs3764002-T	C > T	NR	8.00E-11	[0.015-0.027] unit decrease	N	BMI-adjusted waist	GCST90020024
12	1.08E+08	WSCD2		Waist-to-hip ratio adjusted for BMI	219,872 British ancestry women	rs3764002-T	C > T	NR	5.00E-09	[0.012-0.025] unit decrease	N	BMI-adjusted waist-hip ratio	GCST90020025
12	1.08E+08	WSCD2		Risk-taking tendency (4-domain principal component model)	315,894 European ancestry individuals	rs3764002-T	C > T	0.2616	7.00E-09	[0.011-0.023] unit decrease	N	risk-taking behaviour	GCST007323
12	1.08E+08	WSCD2		General risk tolerance (MTAG)	975,353 European ancestry individuals	rs3764002-T	C > T	0.261	2.00E-17	[0.009-0.014] unit decrease	N	risk-taking behaviour	GCST007325
12	1.08E+08	WSCD2		Waist-hip index	219,872 British ancestry women	rs3764002-T	C > T	NR	6.00E-09	[0.012-0.025] unit decrease	N	BMI-adjusted waist-hip ratio	GCST90020027
12	1.08E+08	WSCD2		Medication use (drugs used in diabetes)	15,272 European ancestry cases, 290,641 European ancestry controls, 30,515 East Asian ancestry cases, 148,211 East Asian ancestry controls	rs3764002-T	C > T	NR	4.00E-08	[0.028-0.059] unit decrease	N	Drugs used in diabetes use measurement	GCST90018981
12	1.08E+08	WSCD2		Waist circumference adjusted for body mass index	219,872 British ancestry women	rs3764002-T	C > T	NR	6.00E-09	[0.012-0.025] unit decrease	N	BMI-adjusted waist	GCST90020029
12	1.08E+08	WSCD2		Body mass index or osteoarthritis (pleiotropy)	434,794 European ancestry BMI females, 90,838 European ancestry osteoarthritis female cases, 192,697 European ancestry female controls	rs3764002-T	C > T	NR	6.00E-12		N	body mass index, osteoarthritis	GCST90271769
12	14434367	ATF7IP		Prostate-specific antigen levels	3,192 European ancestry individuals	rs3213764-G	A > G	0.467	2.00E-09	% increase	N	obsolete_prostate specific antigen	GCST001799
13	28050157	FLT3		Diastolic blood pressure	1,028,980 European ancestry individuals	rs1933437-A	G > A	NR	8.00E-06	[0.039-0.099] unit increase	N	diastolic blood pressure	GCST90310295
13	28050157	FLT3		Body mass index (MTAG)	694,649 European ancestry individuals	rs1933437-A	G > A	NR	7.00E-17	[0.011-0.018] unit decrease	N	body mass index	GCST90179150
13	28050157	FLT3		Diastolic blood pressure	1,164,961 European ancestry individuals	rs1933437-A	G > A	0.6193	3.00E-09	[0.056-0.113] mmHg increase	N	diastolic blood pressure	GCST90292474
13	28050157	FLT3		Diastolic blood pressure	810,865 European ancestry individuals	rs1933437-A	G > A	0.62	2.00E-08	[0.061-0.128] mmHg increase	N	diastolic blood pressure	GCST90000059

13	28050157	FLT3		Systolic blood pressure	1,028,980 European ancestry individuals	rs1933437-A	**G > A**	NR	1.00E-06	[illegible]	[0.071-0.167] unit increase	N	systolic blood pressure	GCST90310294
14	1.04E+08	ASPG		Serum metabolite levels	3,926 Hispanic/Latino individuals	rs8012505-G	**C > G**	0.0994463	5.00E-69	[illegible]	[0.6-0.76] unit increase	N	serum metabolite	GCST012020
14	1.04E+08	ASPG		Serum metabolite levels	3,926 Hispanic/Latino individuals	rs8012505-G	**C > G**	0.0994463	3.00E-15	[illegible]	[0.23-0.38] unit increase	N	serum metabolite	GCST012020
14	1.04E+08	ASPG		Asparagine levels	2,466 Black/admixed ancestry individuals	rs8012505-G	**C > G**	NR	4.00E-29	[illegible]	[0.46-0.66] unit increase	N	asparagine measurement	GCST90176156
14	1.04E+08	ASPG		Asparagine levels	6,136 Finnish ancestry individuals	rs8012505-G	**C > G**	NR	##########	[illegible]	unit increase	N	asparagine measurement	GCST90139606
14	1.04E+08	ASPG		X-25422 levels	6,136 Finnish ancestry individuals	rs8012505-G	**C > G**	NR	3.00E-11	[illegible]	unit decrease	N	X-25422 measurement	GCST90140640
14	1.04E+08	ASPG		X-23739 levels	14,296 European ancestry individuals	rs8012505-C	**C > G**	0.8573	##########	[illegible]	[0.29-0.35] unit decrease	N	X-23739 measurement	GCST90245730
14	1.04E+08	ASPG		Asparagine levels	14,296 European ancestry individuals	rs8012505-C	**C > G**	0.858	##########	[illegible]	[0.41-0.47] unit decrease	N	asparagine measurement	GCST90245117
14	1.04E+08	ASPG		Asparagine levels	8,245 European ancestry individuals	rs8012505-G	**C > G**	0.128388	##########	[illegible]	[0.59-0.67] unit increase	N	asparagine measurement	GCST90200452
14	1.04E+08	ASPG		N-acetylasparagine levels	7,935 European ancestry individuals	rs8012505-G	**C > G**	0.131248	1.00E-30	[illegible]	[0.22-0.31] unit increase	N	blood N-acetylasparagine measurement	GCST90200678
14	1.04E+08	ASPG		Metabolite levels (N-acetylasparagin	6,183 European, Hispanic or African American individuals	rs8012505-G	**C > G**	0.1098	3.00E-25	[illegible]	[0.24-0.36] unit increase	N	blood N-acetylasparagine measurement	GCST90300389
14	1.04E+08	ASPG		Metabolite levels (asparagine)	10,665 European, Hispanic or African American	rs8012505-G	**C > G**	0.1147	##########	[illegible]	[0.46-0.55] unit increase	N	asparagine measurement	GCST90299545
14	1.05E+08	AHNAK2		Circulating levels of total-tau	953 African American individuals	rs2396457-A	**G > A**	0.38	3.00E-07	[illegible]	[0.21-0.49] unit decrease	N	t-tau measurement	GCST90095139
14	20117883	OR4K17		F-healthy breakfast food liking (derived food-liking factor)	143,879 European ancestry individuals	rs8005245-C	**G > C**	0.4116	8.00E-11	[illegible]	[0.061-0.113] unit decrease	N	taste liking measurement	GCST90094780
14	20117883	OR4K17		Porridge liking	159,122 European ancestry individuals	rs8005245-C	**G > C**	0.4115	2.00E-10	[illegible]	[0.016-0.03] unit decrease	N	taste liking measurement	GCST90094815
