# Supplementary material for: High-coverage whole-genome sequencing of a Jakun individual from the “Orang Asli” Proto-Malay subtribe from Peninsular Malaysia
Source: Hum Genome Var. 2025 Jan 8;12:4. doi: 10.1038/s41439-024-00308-6 (PMC11707147; doi:10.1038/s41439-024-00308-6)
Supplement: Supplementary file 12 — Table S6 [file 41439_2024_308_MOESM12_ESM.pdf]

Table S6 Position of 17 novel nsSNVs in the Jakun genome. The Jakun genome was annotated using db151, but no rsID was found

Position	rsID	REF	ALT	Gene Identified
X:48434982	.	T	C	RBM3
X:30714172	.	G	C	GK
1:186031717	.	A	C	HMCN1
1:247769747	.	C	G	OR2G3
4:56212663	.	A	G	SRD5A3
6:56371562	.	A	C	DST
7:111970151	.	G	T	ZNF277
13:37679023	.	A	G	CSNK1A1L
15:85384002	.	C	T	ALPK3
16:46934663	.	G	T	GPT2
18:28611017	.	T	A	DSC3
19:4323125	.	C	A	FSD1
19:9083985	.	C	A	MUC16
19:18375480	.	C	A	KIAA1683
20:32000162	.	A	C	SNTA1
20:3652792	.	A	G	ADAM33
21:27277359	.	T	C	APP
