# Supplementary material for: High-coverage whole-genome sequencing of a Jakun individual from the “Orang Asli” Proto-Malay subtribe from Peninsular Malaysia
Source: Hum Genome Var. 2025 Jan 8;12:4. doi: 10.1038/s41439-024-00308-6 (PMC11707147; doi:10.1038/s41439-024-00308-6)
Supplement: Supplementary file 13 — Table S7 [file 41439_2024_308_MOESM13_ESM.pdf]

Table S7 23 overlapping nsSNVs from Jakun_Seq, CAM and SG_MAS that showed damaging and deleterious effects, consensus from SIFT, PolyPhen2 and CADD (Phred score ≥ 15).

Position	rsID	REF	ALT	Gene Identified	1000 Genomes (alternate allele frequency)		
					Europe	African	East Asian
11:114393652	rs10891692	C	T	NXPE1	0.3370	0.2534	0.6687
1:118565953	rs10923472	G	A	SPAG17	0.4314	0.1551	0.1567
12:29617550	rs11050243	G	A	OVCH1	0.1352	0.3585	0.0853
11:76954833	rs11237146	G	A	GDPD4	0.3608	0.0257	0.2560
17:7761512	rs12453250	C	A	CYB5D1	0.0934	0.3472	0.2728
17:41879074	rs17742683	T	C	MPP3	0.1133	0.0061	0.0367
19:15839365	rs1806931	C	T	OR10H2	0.0527	0.0257	0.6081
1:21795388	rs1827293	A	G	NPBF3	0.5417	0.1445	0.5804
22:39134207	rs2072797	C	T	SUN2	0.1312	0.0068	0.1677
22:36123083	rs2076672	C	T	APOL5	0.2545	0.0923	0.0804
10:44052903	rs2230661	A	C	ZNF239	0.5308	0.4917	0.5506
1:186273994	rs2273779	C	T	PRG4	0.3300	0.1490	0.1290
1:111861974	rs2275254	C	T	CHIA	0.4046	0.6846	0.6200
2:179458591	rs2288569	C	T	TTN	0.1402	0.0666	0.4544
19:372661	rs2303810	C	A	THEG	0.0537	0.0144	0.4623
14:20528528	rs2775254	G	A	OR4L1	0.3221	0.3623	0.5347
8:87235273	rs2976189	C	T	SLC7A13	0.1491	0.1286	0.0179
2:179397561	rs3829747	C	T	TTN	0.1392	0.0666	0.4484
9:286593	rs529208	C	A	DOCK8	0.4841	0.3918	0.7212
11:60291413	rs6591595	C	T	MS4A13	0.8787	0.6059	0.7242
11:5842356	rs8181512	A	G	OR52N2	0.5368	0.3411	0.4692
13:76395620	rs9593132	C	T	LMO7	0.0517	0.0628	0.1210
19:52376207	rs9807842	G	A	ZNF577	0.0358	0.2943	0.0437
