# Supplementary material for: High-coverage whole-genome sequencing of a Jakun individual from the “Orang Asli” Proto-Malay subtribe from Peninsular Malaysia
Source: Hum Genome Var. 2025 Jan 8;12:4. doi: 10.1038/s41439-024-00308-6 (PMC11707147; doi:10.1038/s41439-024-00308-6)
Supplement: Supplementary file 14 — Table S8 [file 41439_2024_308_MOESM14_ESM.pdf]

Table S8 Variants of core ADME genes in the Jakun genome. In total 3,310 variants related to 32 core ADME genes were identified in this genome. Out of 3,310 variants, 193 were relevant to variants found at PharmGKB, in which 21 of those were nsSNVs (in red).

Core ADME Gene	*Gene Length (bp)	**Variant (rsID) found at PharmGKB	Variant (rsID) found at Jakun	***Mut. Burden (%)	Variant in Jakun Relevant to PharmGKB	
					Number	rsID#
ABCB1	210,278	59	139	0.0661	21	rs10234411 (T > A); rs10248420 (A > G); rs10264990 (C > T); rs10267099 (G > A); rs10276036 (C > T); rs1045642 (A > G); rs1186746 (T > C); rs1202170 (C > T); rs1202171 (T > A); rs1922242 (A > T); rs2032582 (A > C); (rs2235048 (G > A); rs2373586 (A > C); rs2520464 (C > T); rs4148737 (T > C); rs4148738 (C > T); rs6949448 (T > C); rs6961419 (T > C); rs6961665 (C > A); rs7787082 (G > A); rs868755 (T >G)
ABCC2	69,954	35	95	0.1358	6	rs1885301 (A > G); rs2002042 (C > T); rs3740074 (C > T); rs4148386 (G > A); rs4148396 (T > C); rs4148398 (A > G)
ABCG2	141,362	52	272	0.1924	21	rs10011796 (T > C); rs1481012 (A > G); rs1564481 (C > T); rs17731799 (G > T); rs2199936 (A > G); rs2231142 (G > T); rs2622604 (T > C); rs2622621 (C > G); rs2622628 (A > C); rs2725252 (C > A); rs2725256 (A > G); rs2725263 (A > C); rs2725264 (C > T); rs3109823 (C > T); rs3114018 (A > C); rs3114020 (T > C); rs4148155 (A > G); (rs4148157 (G > A); rs45499402 (G > C); rs6857600 (C > T); rs76979899 (C > T)
CYP1A1	5,986	7	7	0.1169	4	rs1048943 (T > C); rs3826041 (A > C); rs4646421 (G > A); rs4646903 (A > G)
CYP1A2	7,762	24	14	0.1803	2	rs2069514 (G > A); rs762551 (C > A)
CYP2A6	6,906	46	21	0.3041	7	rs1137115 (T > C); rs56113850 (T > C); rs57837628 (A > G); rs7259706 (C > T); rs7260629 (T > G); rs8192725 (A > G); rs8192733 (G > C)
CYP2B6	27,116	64	59	0.2176	11	rs2279342 (A > T); rs2279344 (G > A); rs2279345 (T > C); rs3181842 (C > T); rs3760657 (A > G); rs4802101 (T > C); rs4803419 (C > T); rs707265 (A > G); rs7246465 (T > C); rs7254579 (T > C); rs8100458 (T > C)

CYP2C19	92,866	80	160	0.1723	6	rs11188082 (A > T); rs11528090 (T > G); rs11568732 (T > G); rs12768009 (G > A); rs3814637 (C > T); rs4986893 (G > A)
CYP2C8	32,725	20	63	0.1925	7	rs1058932 (G > A); rs10882521 (G > T); rs1113129 (G > C); rs1934951 (C > T); rs1934980 (A > G); rs2275622 (T > C); rs7910936 (C > T)
CYP2C9	51,433	78	64	0.1244	8	rs1934968 (A > G); rs1934969 (A > T); rs2860905 (G > A); rs4086116 (C > T); rs4917639 (A > C); rs4918758 (T > C); rs9332096 (C > T); rs9332127 (G > C)
CYP2D6	4,311	35	29	0.6727	7	rs1058164 (G > C); rs1080983 (T > C); rs1080985 (C > G); rs1135840 (G > C); rs16947 (A > G); rs28371699 (A > C); rs28371713 (A > G)
CYP2E1	11,760	8	47	0.3996	3	rs2070673 (A > T); rs2070676 (G > C); rs2515641 (T > C)
CYP3A4	27,217	23	41	0.1506	9	rs12333983 (T > A); rs1851426 (A > G); rs2242480 (C > T); rs2246709 (A > G); rs2687116 (C > A); rs2740574 (C > T); rs3735451 (T > C); rs4646437 (G > A); rs4646440 (G > A)
CYP3A5	31,802	14	61	0.1918	4	rs15524 (A > G); rs4646450 (G > A); rs4646453 (C > A); rs776746 (C > T)
DPYD	843,316	204	874	0.1036	13	rs1042482 (C > T); rs12022243 (C > T); rs17116806 (C > A); rs1760217 (A > G); rs1801159 (T > C); rs1801265 (G > A); rs2786783 (G > A); rs2811178 (T > C); rs291592 (C > T); rs291593 (G > A); rs4970722 (A > T); rs72728438 (T > C); rs7548189 (C > A)
GSTM1	5,928	1	2	0.0337	0	-
GSTP1	2,841	4	10	0.3520	1	rs4147581 (C > G)
GSTT1	8,178	3	0	0.0000	0	-
NAT1	53,222	1	180	0.3382	0	-
NAT2	9,936	11	39	0.3925	5	rs1041983 (C > T); rs1208 (G > A); rs1799931 (G > A); rs4271002 (G > C); rs4646267 (A > G)

SLC15A2	49,787	3	154	0.3093	3	rs1143671 (C > T); rs1143672 (G > A); rs2257212 (C > T)
SLC22A1	36,903	16	51	0.1382	6	rs1867351 (T > C); rs594709 (G > A); rs622342 (C > A); rs628031 (A > G); rs683369 (G > C); rs6935207 (G > A)
SLC22A2	42,066	7	97	0.2306	4	rs315978 (T > C); rs316003 (C > T); rs316009 (T > C); rs316019 (A > C)
SLC22A6	8,370	4	3	0.0358	0	-
SLCO1B1	108,602	43	223	0.2053	16	rs11045879 (T > C); rs2291073 (T > G); rs2291075 (C > T); rs2306283 (A > G); rs4149014 (T > G); rs4149026 (A > C); rs4149032 (C > T); rs4149034 (G > A); rs4149035 (T > C); rs4149036 (C > A); rs4149044 (A > T); rs4149045 (G > A); rs4149081 (G > A); rs4149087 (T > G); rs4363657 (T > C); rs58310495 (C >T)
SLCO1B3	106,206	12	379	0.3568	6	rs11045585 (A > G); rs2053098 (A > G); rs2417940 (T > C); rs4149117 (T > G); rs7311358 (G > A); rs7977213 (G > C)
SULT1A1	18,117	12	32	0.1766	3	rs1801030 (C > T); rs28374453 (A > G); rs3760091 (C > G)
TPMT	26,858	15	46	0.1713	5	rs12529220 (T > A); rs2518463 (A > G); rs2842934 (G > A); rs2842949 (C > A); rs4449636 (G > A)
UGT1A1	13,030	24	4	0.0307	0	-
UGT2B15	24,055	3	12	0.0540	1	rs4148269 (T > G)
UGT2B17	39,149	1	43	0.1098	0	-
UGT2B7	61,612	30	89	0.1445	14	rs10006452 (T > C); rs11940316 (T > C); rs28375964 (C > T); rs4292394 (C > G); rs4455491 (A > G); rs4554144 (C > T); rs6600879 (C > G); rs6600880 (T > A); rs6600893 (T > C); rs6851533 (T > C); rs7438135 (G > A); rs7439366 (T > C); rs7662029 (A > G); rs7668258 (T > C)

*Gene length (GRCh37) was obtained from NCBI (https://www.ncbi.nlm.nih.gov/gene) [Accessed 9 Oct 2024]

**Variant (rsID) found at PharmGKB (https://www.pharmgkb.org/) [Accessed 9 Oct 2024]

***Mutation burden was calculated based on [No of variant (rsID) found at Jakun / Gene Length (bp)] X 100%
