# Supplementary material for: High-coverage whole-genome sequencing of a Jakun individual from the “Orang Asli” Proto-Malay subtribe from Peninsular Malaysia
Source: Hum Genome Var. 2025 Jan 8;12:4. doi: 10.1038/s41439-024-00308-6 (PMC11707147; doi:10.1038/s41439-024-00308-6)
Supplement: Supplementary file 15 — Supplementary figure and table legend [file 41439_2024_308_MOESM15_ESM.docx]

**Supplementary Information**

**Supplementary Note** Detailed protocol for SNV and indel identification

**Figure S1** Flow chart of dataset merging.

**Figure S2** Percentage of novel SNVs in each chromosome (autosome).

**Figure S3** Distribution of SNVs found in the Jakun individual.

**Figure S4** Cross-validation plot for the global populations.

**Figure S5** Maximum-likelihood tree generated using TreeMix with 100 bootstraps, assuming 1–10 gene flow events.

**Table S1** Biological materials (sequencing and genotyping data) of the populations used in this study.

**Table S2** Percentage of Admixture components from global populations.

**Table S3** F3 and F4 statistics of the Jakun_Seq+Geno and its neighboring populations.

**Table S4** Potentially damaging and deleterious variants as predicted by the computational tools SIFT, Polyphen-2 and CADD (Phred score ≥ 15).

**Table S5** Reported traits for 72 nsSNVs in the Jakun individual from the GWAS catalog.

**Table S6** Positions of 17 novel nsSNVs in the Jakun genome. The Jakun genome was annotated using db151, but no rsID was found.

**Table S7** Twenty-three overlapping nsSNVs from Jakun_Seq, CAM and SG_MAS that showed damaging and deleterious effects, with a consensus from SIFT, PolyPhen2 and CADD (Phred score ≥ 15).

**Table S8** Variants of core ADME genes in the Jakun genome.
